# Cumulative Evidence for Associations Between Genetic Variants in Interleukin 6 Receptor Gene and Human Diseases and Phenotypes

**DOI:** 10.3389/fimmu.2022.860703

**Published:** 2022-04-14

**Authors:** Min Zhang, Ye Bai, Yutong Wang, Huijie Cui, Mingshuang Tang, Lanbing Wang, Xin Wang, Dongqing Gu

**Affiliations:** ^1^ School of Public Health and Management, Chongqing Medical University, Chongqing, China; ^2^ Department of Epidemiology and Medicine, West China School of Public Health and West China Fourth Hospital, Sichuan University, Chengdu, China; ^3^ Division of Medical Affairs, The First Affiliated Hospital of Army Military Medical University, Chongqing, China; ^4^ Department of Infectious Diseases, First Affiliated Hospital, Army Medical University, Chongqing, China

**Keywords:** interleukin 6 receptor, variant, cardiovascular diseases, inflammatory diseases, phenotypes

## Abstract

**Background:**

Genetic studies have linked polymorphisms in the interleukin 6 receptor (*IL6R*) gene to the risk of multiple human diseases and phenotypes, yet have reported inconsistent results. We aimed to synthesize current knowledge of variants in the *IL6R* gene on the risk of diseases and phenotypes.

**Methods:**

We searched the Medline and Embase databases to identify relevant publications. Meta-analysis was performed utilizing DerSimonian and Laird random-effects model. We also graded cumulative evidence for significant associations. Furthermore, phenome-wide analyses and functional annotations were performed for variants with strong evidence.

**Results:**

We included 155 studies for evaluating the associations between 80 polymorphisms in the *IL6R* gene and the risk of 102 human diseases and 98 phenotypes. We conducted 58 main meta-analyses, and 41 significant associations were identified. Strong evidence was assigned to 29 associations that investigated ten variants (rs2228145, rs4129267, rs7529229, rs4537545, rs7518199, rs4845625, rs4553185, rs4845618, rs4845371, and rs6667434) related to the risk of four cardiovascular diseases (coronary heart disease, coronary artery disease, atherosclerosis, and abdominal aortic aneurysms), four inflammatory diseases (rheumatoid arthritis, Crohn’s disease, dermatitis, and asthma), and concentration of four phenotypes (C-reactive protein, fibrinogen, IL-6, and sIL-6R). Furthermore, phenome-wide analysis verified that rs2228145 associated with asthma and dermatitis risk. Functional analyses indicated that these polymorphisms fall within exon, enhancer regions.

**Conclusions:**

Our study comprehensively summarizes current data on the genetic architecture of the *IL6R* gene and highlights the pharmacological targeting potential of IL-6R on cardiovascular and inflammatory diseases.

## 1 Introduction

Interleukin (IL)-6 receptor (*IL6R*) gene is located in chromosome 1q21.3 (Gene ID: 3570) (1). Its gene production, IL-6R, could specifically bind to its ligand (IL-6) and subsequently recruit two molecules of glycoprotein 130 (gp130) to initiate intercellular signaling (2, 3). IL-6/IL-6R/gp130 signaling is implicated in the development of inflammatory and autoimmune diseases, including novel coronavirus disease 2019 (COVID-19) (4).

Genetic studies have indicated that variants in the *IL6R* gene are associated with a range of common diseases and phenotypes. In 2003, Kim et al. firstly identified seven single-nucleotide polymorphisms (SNPs) in the *IL6R* gene in the Korean population (5). One of these variants, Asp358Ala (rs2228145, previously rs8192284), a non-synonymous located in exon 9, has been shown to be associated with the index of obesity in Indians (6) in the same year. Subsequent studies have implicated that Asp358Ala was associated with a series of inflammatory/autoimmune diseases, including inflammatory bowel disease (7), rheumatoid arthritis (8), cardiovascular diseases (CVDs) (9, 10), and type 2 diabetes (11). In 2012, rs2228145 was implicated to play a protective effect in the etiology of CVD diseases, including coronary heart disease (CHD), atrial fibrillation (AF), and abdominal aortic aneurysms (AAA) (12). This effect was verified by findings from a large phenome-wide association analysis among populations from the Million Veteran Program, which showed that rs2228145 was significantly associated with 22 diseases (13). In addition to CVDs, rs2228145 was found to decrease the risk of ulcerative colitis (UC) or Crohn’s disease (CD) by a genome-wide meta-analysis of 38,197 patients with IBD and more than 40,000 controls (7). A genome-wide association study (GWAS) of European ancestry identified that rs2228145 also decreased risk of rheumatoid arthritis (8), and this association has been replicated by subsequent studies (14). Additionally, rs2228145 plays a protective effect in the etiology of type I diabetes (T1D) (12) while positively associated with dermatitis and renal function disorder (13).

Other polymorphisms in the *IL6R* gene, such as rs4129267 (T allele), rs4845625 (C allele), or rs7529229 (C allele), were also reported to be associated with the risk of many diseases, such as asthma (15) and CVDs (16). A GWAS in Australia identified that rs4129267 (T allele) significantly increased risk of asthma (OR = 1.09, combined *p* = 2.4×10^−8^) (15). Then, another GWAS in the Caucasian population indicated that this variant was also associated with an increased risk of AAA. Other large-scale genetic analyses identified that rs4845625 (C allele) and rs7529229 (C allele) were implicated in risk of CVDs, such as atrial fibrillation (17), coronary artery disease (CAD) (16), AAA, and CHD (10).

In addition to diseases, variants in the *IL6R* gene were also suggested to be related to hematological inflammatory biomarkers and some conventional CVD risk factors. Several publications showed that the C allele of rs2228145 increased circulating soluble IL-6R (sIL-6R) and IL-6 concentrations by 34% and 15%, respectively (7, 9). Furthermore, rs4845371, rs6667434, or rs7529229 was associated with lower levels of circulation of C-reactive protein (CRP), fibrinogen, troponin I, creatine kinase-MB (CK-MB), pro-B-type natriuretic peptide, and systolic blood pressure, which were consistent with a reduced risk of CVDs (9, 13). With the increasing number of genetic studies published since 2003, several meta-analyses have been performed to investigate the role of polymorphisms in *IL6R* gene on the risk of human diseases and phenotypes. However, existing meta-analyses were limited to a single variant or illness, and the results might not be adequate partly due to the small sample size (18–21). These drawbacks may limit the implementation of the evidence in the clinical practice and disease prevention strategies. Therefore, we aimed to provide a systematic synopsis on the associations between polymorphisms in the *IL6R* gene and human diseases and phenotypes. Firstly, we conducted a systematic review and meta-analysis to evaluate the epidemiological associations between variants in the *IL6R* gene and human diseases and phenotypes. Secondly, we graded levels of cumulative evidence for significant associations combining Venice criteria and false-positive report probability (FPRP) tests. Thirdly, we performed a phenome-wide analysis for independent variants with strong evidence using data from UK Biobank to uncover new relationships. Fourthly, we performed functional annotations for the variants in the *IL6R* gene using data from the Encyclopedia of DNA Elements Project (ENCODE), the 1000 Genomes Project, the Genotype-Tissue Expression (GTEx) Project, and other sources to provide clues for the pathogenesis of these associations.

## 2 Materials and Methods

The methods used in the present study were based on guidelines proposed by the Human Genome Epidemiology Network (HuGENet) for a systematic review of genetic association studies and the Preferred Reporting Items for Systematic Reviews and Meta-Analyses (PRISMA) (22, 23). This study has been registered to the International Prospective Register of Systematic Reviews (PROSPERO; registration ID: CRD42020201735).

### 2.1 Literature Search Strategy, Study Eligibility, and Inclusion Criteria

We systematically searched PubMed and Embase using keywords (“interleukin 6 receptor” OR “IL-6R” OR “IL6R” OR “interleukin-6 receptor” OR “IL-6 receptor” OR “rs2228145” OR “Asp358Ala” OR “D358A”) to identify the original genetic association studies of *IL6R* published in English up to August 1, 2021. We also retrieved published reviews, meta-analyses, pooled analyses, and the included studies to identify additional publications.

Articles were eligible to be included if they met the following inclusion criteria: (i) they were observational studies conducted in humans; (ii) they investigated associations of genetic variants in *IL6R* gene and human diseases or phenotypes; (iii) they directly provided risk estimates such as odds ratio (OR), relative risk (RR), hazard ratio (HR), and 95% confidence interval (CI), coefficient *β* and standard error (SE), or provided valid data to calculate these risk estimates under additive genetic model; (iv) or for publications investigating continuous quantitative phenotypes, they should provide mean and standard deviation (SD). When multiple studies use the same population, the most recent or the one with the largest sample size was selected. Studies without full text or sufficient information to calculate effect size were excluded. Two investigators (MZ and YB) independently assessed the eligibility of each publication, and disagreement was settled through discussion with the principal author (DG).

### 2.2 Data Extraction, Preparation, and Management

Three authors (MZ, YW, and XW) independently extracted the data using a predesigned collection sheet including PMID, first author, publishing year, variants, major and minor alleles, source of population, ethnicity of the study population, study design, sample size of cases and controls, genotype and allele counts, matched information, effects of associations and the corresponding 95% CIs, regression coefficients, SE or *p*-value (for studies using multiple adjusted models, the most fully adjusted estimates were extracted), mean concentrations of quantitative traits and SD for different genotypes, Hardy–Weinberg equilibrium (HWE) among controls, and genotyping methods.

### 2.3 Statistical Analysis

In our study, the primary outcome was the risk of disease or categorical phenotypes, measured by OR/RR/HR and 95% CI under an additive genetic model. Due to the relatively low incidence of the events mentioned above, the effect size of OR, HR, and RR was deemed identical in this meta-analysis (24). For continuous quantitative phenotypes, the primary outcome was the mean deviation, measured by standardized mean difference (SMD) and 95% CI after pooling mean concentration and SD under a co-dominant model (mutant-type homozygote vs. wild-type homozygote, heterozygote vs. wild-type homozygote).

Meta-analysis was conducted for variants with at least three independent studies or datasets. We estimated the effects of minor alleles (in Caucasians) on the outcome for each variant. We adopted the methods used in original studies if data were extracted from GWASs or large GWAS meta-analyses from collaborative studies. For heterogeneity assessment, we performed the Cochran’s *Q* test, and the *I* ^2^ statistic was used to evaluate and quantify heterogeneity between studies (25, 26). A random-effects model was performed if heterogeneity was evident (*I* ^2^ ≥ 50%), while the fixed effects model was used if *I* ^2^ < 50%. For variants that showed significant associations with relevant diseases or phenotypes, sensitivity analyses were conducted to examine whether the associations would be lost when a single study was excluded or when the first positive report or studies with deviation from HWE in controls were excluded. Begg’s test (27) and Egger’s test (28) were conducted to evaluate potential publication bias and small-study bias, respectively.

Statistical analysis was done using STATA, version 15 (Stata, College Station, TX). Tests of heterogeneity and bias were two-tailed, and a *p-*value of less than 0.10 was considered significant as recommended. All the other statistical tests were two-tailed, and a *p-*value of < 0.05 was considered statistically significant unless otherwise stated.

### 2.4 Assessment of Cumulative Evidence

Venice criteria were applied to evaluate the epidemiological credibility of significant associations identified by primary meta-analyses (see **Supplementary Notes**) (29). Briefly, credibility was determined by three criteria: (i) amount of evidence, (ii) replication, and (iii) protection from bias. Based on the grade of A, B, or C in these criteria, epidemiological credibility was defined as strong when all three grades were A, moderate when all three grades were A or B, and weak when any grade was C.

We also estimated the noteworthiness of the significant associations by calculating FPRP (30). A prior probability was set at 0.05 to estimate the FPRP value based on the OR obtained from a meta-analysis, and we adopted an FPRP cutoff value of 0.20. Briefly, FPRP value < 0.05, 0.05 < FPRP value < 0.20, and FPRP value > 0.20 were defined as strong, moderate, and weak evidence of true association, respectively. The cumulative epidemiological evidence was adjusted based on the FPRP results: the evidence may be upgraded if FPRP evidence was strong and downgraded if the FPRP result was weak.

### 2.5 Phenome-Wide Analysis

We performed linkage disequilibrium (LD) analysis for variants significantly associated with risk of diseases and phenotypes in the present meta-analyses, and *r* ^2^ > 0.80 was considered as significant using data from the 1000 Genomes Project (31). We then conducted a phenome-wide analysis to estimate associations of the independent variants with 778 phenotypes from UK Biobank in GeneATLAS through querying summary data (32). We considered *p*-values < 6.43 × 10^-5^ (0.05/778) as significant, adjusted for multiple comparisons of variants and 778 phenotypes.

### 2.6 Functional Annotations

We conducted analyses to investigate the potential regulatory effect of polymorphisms significantly associated with relative diseases or phenotypes using the Encyclopedia of DNA Elements (ENCODE) tool HaploReg v4.1 (33) and the University of California, Santa Cruz (UCSC) Genome Browser (34). We examined genome-wide *cis*-eQTL data in multiple tissues from the Blood eQTL browser (35), and the GTEx Project (36). The significance threshold for these analyses was set to *p*-values of less than 0.005 accounting for 10 tests. In addition, we searched the published literature for the *IL6R* gene in relation to diseases from PubMed.

## 3 Results

### 3.1 Characteristics of the Included Studies

**Figure 1:**
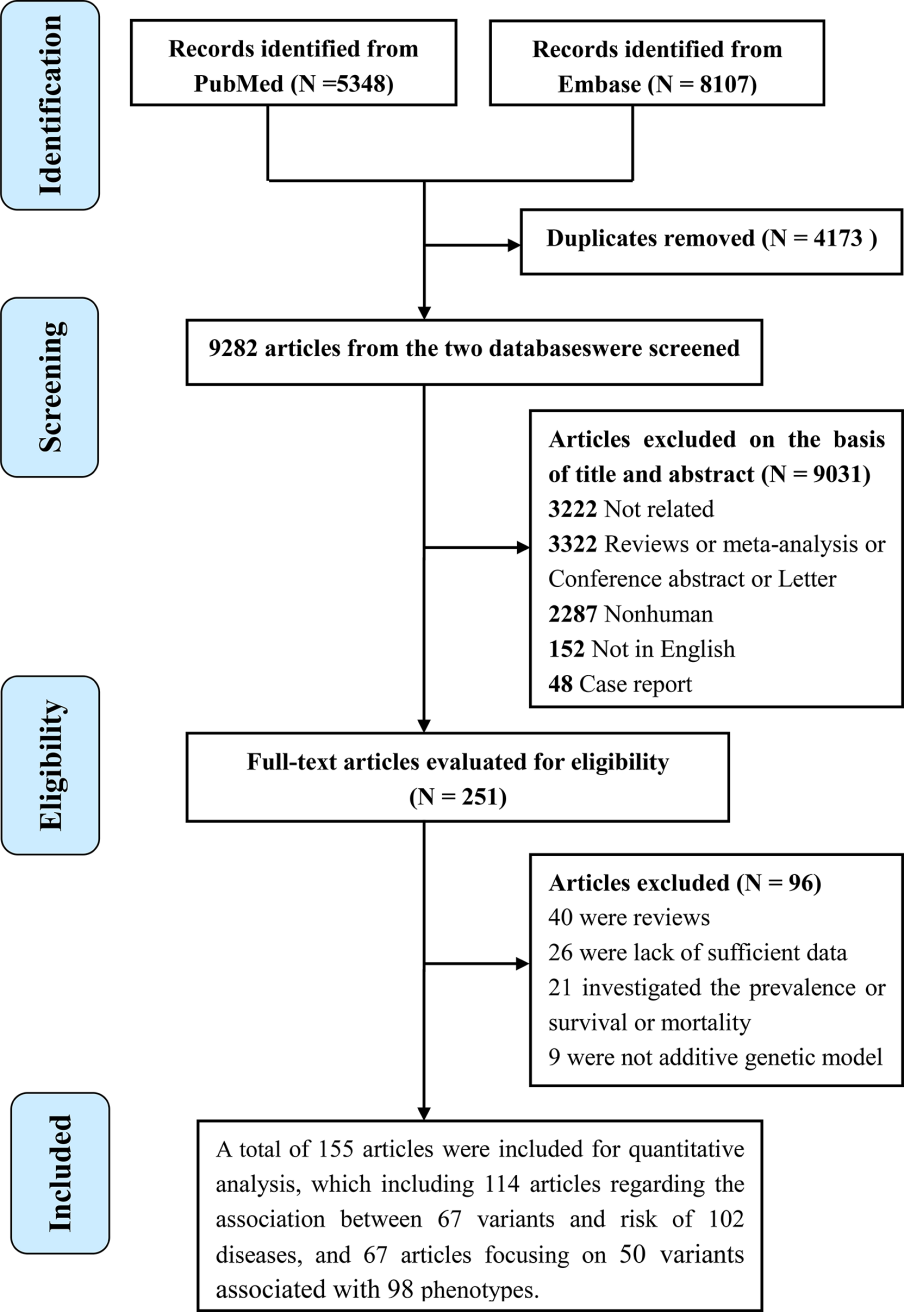
Flowchart of the study selection in the present study.

The literature search strategy is shown in **Figure 1**. A total of 155 studies were included for evaluating the associations between 80 polymorphisms in the *IL6R* gene and the risk of human diseases and phenotypes. Of the 155 publications, 114 regarding the association between 67 variants in the *IL6R* gene and 102 diseases (**Supplementary Table 1**) and 67 regarding 50 variants associated with 98 phenotypes (**Supplementary Tables 2, 3**) were included. A total of 131 articles were cross-sectional or case–control studies, and 24 articles were longitudinal studies. As for the study population, 94 were conducted in populations of European ancestry, 34 were in Asians, four were in the African population, four were in the population of mixed ancestry, and 19 were on other ancestries. The mean number of cases and controls in these articles was 3,855 (range, 17 to 51,441) and 33,415 (range, 20 to 897,488), respectively. Of the 155 studies, 134 were published since 2010 (86.345%).

### 3.2 Relationships Between Variants in *IL6R* Gene and Human Diseases/Phenotypes by the Meta-Analyses

#### 3.2.1 Relationships Between Variants in *IL6R* Gene and Human Diseases

We conducted 27 main meta-analyses to estimate associations of six variants in *IL6R* gene with risk of 20 kinds of diseases under an additive model. Summary findings of meta-analyses are shown in **Table 1**. Of the 27 main analyses, 21 associations between six variants and 14 diseases indicated statistical significance (*p* < 0.05). Specifically, the coronary heart disease risk was negatively associated with minor allele of rs7529229 (OR = 0.954, 95% CI: 0.932–0.977), rs4537545 (OR = 0.940, 95% CI: 0.903–0.978), and rs2228145 (OR = 0.957, 95% CI: 0.948–0.967). Atrial fibrillation risk was inversely associated with minor allele of rs7529229 (OR = 0.900, 95% CI: 0.850–0.960), rs4537545 (OR = 0.901, 95% CI: 0.847–0.952), and rs28638007 (OR = 0.900, 95% CI: 0.850–0.950), but positively associated with rs4845625 (OR = 1.179, 95% CI: 1.044–1.330). The abdominal aortic aneurysms risk was negatively associated with rs7529229 (OR = 0.841, 95% CI: 0.800–0.884), but it was positively associated with rs4129267 (OR = 1.141, 95% CI: 1.101–1.183). As for asthma, rs4129267 (OR = 1.073, 95% CI: 1.035–1.112) and rs2228145 (OR = 1.053, 95% CI: 1.023–1.084) were associated with increased risk of asthma. In addition, the CAD risk was positively associated with rs4845625 (OR = 1.060, 95% CI: 1.042–1.079).

**Table 1:** Variants in the *IL6R* gene associated with risk of human diseases in meta-analysis.

Variant	Allele^a^	MAF	Diseases	Datasets	Cases	Controls	Risk of human diseases		Heterogeneity		Venice criteria grade	FPRP	Cumulative evidence of association
							OR (95%CI)	*p*	*І* ^2^	*p*			
rs7529229	T/C	0.3535	CHD	36	26,310	101,971	0.954 (0.932, 0.977)	1.07×10^-4^	0.0%	0.686	AAA	0.002	Strong
			AAA	5	4,529	15,734	0.841 (0.800, 0.884)	1.38×10^-11^	8.6%	0.322	AAA	<0.001	Strong
			Atrial fibrillation	5	2,728	16,702	0.900 (0.850, 0.960)	0.001	NA		B-C	0.026	Weak
rs4845625	C/T	0.4495	CAD	47	63,434	110,256	1.060 (1.042, 1.079)	1.12×10^-10^	15.7%	0.180	AAA	<0.001	Strong
			Atrial fibrillation	7	2,991	20,101	1.179 (1.044, 1.330)	0.007	52.4%	0.122	ACA	0.187	Weak
rs4537545	C/T	0.3535	CHD	8	13,370	30,425	0.940 (0.903, 0.978)	0.002	27.7%	0.207	ABC	0.040	Moderate
			Atrial fibrillation	5	2,728	16,702	0.901 (0.847, 0.952)	2.32×10^-4^	NA		B-C	0.004	Moderate
rs4129267	C/T	0.3485	AAA	7	10,204	107,766	1.141 (1.101, 1.830)	4.83×10^-13^	0.0%	0.655	AAA	<0.001	Strong
			Asthma	7	62,596	389,460	1.073 (1.035, 1.112)	1.18×10^-4^	61.1%	0.017	AAA	<0.001	Strong
rs28638007	T/C	0.3956	Atrial fibrillation	5	2,728	16,702	0.900 (0.850, 0.950)	1.07×10^-4^	NA		B-C	0.003	Moderate
rs2228145	A/C	0.3485	Aneurysm	5	27,819	352,035	0.881 (0.851, 0.912)	8.68×10^-13^	71.4%	0.007	AAA	<0.001	Strong
			Atherosclerosis	4	91,520	327,474	0.929 (0.908, 0.951)	6.02×10^-10^	63.9%	0.040	AAA	<0.001	Strong
			CHD	5	204,050	1,033,873	0.957 (0.948, 0.967)	3.17×10^-17^	0.0%	0.212	AAA	<0.001	Strong
			Crohn’s disease	3	32,880	70,025	0.946 (0.927, 0.964)	2.14×10^-8^	0.0%	0.950	AAA	<0.001	Strong
			Dermatitis	5	141,581	326,743	1.048 (1.026, 1.071)	2.31×10^-5^	77.0%	0.002	AAA	<0.001	Strong
			Asthma	5	28,762	105,138	1.053 (1.023, 1.084)	4.86×10^-4^	2.9%	0.390	AAA	0.009	Strong
			Ulcerative colitis	3	30,076	75,562	0.977 (0.957, 0.996)	0.018	0.0%	0.672	AAA	0.254	Moderate
			RA	6	18,830	51,755	0.790 (0.693, 0.901)	4.48×10^-4^	88.0%	0.000	AAA	0.004	Strong
			Type 1 diabetes	4	38,522	780,577	0.940 (0.898, 0.984)	0.009	70.1%	0.018	AAA	0.234	Moderate
			Type 2 diabetes	5	276,906	1,441,968	0.975 (0.952, 0.998)	0.033	34.1%	0.194	AAA	0.388	Moderate
			CVD	4	2,335	35,900	0.726 (0.535, 0.983)	0.040	80.2%	0.002	BCC	0.796	Weak
			Obesity	3	1,007	402	1.084 (0.796, 1.476)	0.609	52.0%	0.124			
			Dengue	4	680	850	0.837 (0.494, 1.417)	0.507	73.4%	0.010			
			Mental disorder	7	1,845	8,481	1.051 (0.908, 1.216)	0.505	38.1%	0.138			
			Multiple myeloma	4	958	433	1.129 (0.791, 1.610)	0.505	64.8%	0.036			
			COPD	6	7,519	35,653	1.019 (0.965, 1.076)	0.497	0.0%	0.850			
			ACL	3	406	411	0.937 (0.735, 1.194)	0.597	21.9%	0.278			

a Major allele/Minor allele. Venice criteria grade was determined by amount of evidence, replication, and protection from bias. CHD, coronary heart disease; AAA, abdominal aortic aneurysms; CAD, coronary artery disease; RA, rheumatoid arthritis; ACL, anterior cruciate ligament injury; COPD, chronic obstructive pulmonary disease; CVD, cardiovascular disease; NA, Not Available.

Furthermore, rs2228145 (C allele) was associated with decreased risk of aneurysm (OR = 0.881, 95% CI: 0.851–0.912), atherosclerosis (OR = 0.929, 95% CI: 0.908–0.951), rheumatoid arthritis (OR = 0.790, 95% CI: 0.693–0.901), type 1 diabetes (OR = 0.940, 95% CI: 0.898–0.984), CVD (OR = 0.726, 95% CI: 0.535–0.983), type 2 diabetes (OR = 0.975, 95% CI: 0.952–0.998), Crohn’s disease (OR = 0.946, 95% CI: 0.927–0.96), and ulcerative colitis (OR = 0.977, 95% CI: 0.957–0.996) while it was positively associated with dermatitis (OR = 1.048, 95% CI: 1.026–1.071). However, we did not find significant associations between rs2228145 and risk of obesity, dengue, mental disorder, multiple myeloma, chronic obstructive pulmonary disease (COPD), and anterior cruciate ligament injury (*p* > 0.05).

#### 3.2.2 Relationships Between Variants in *IL6R* Gene and Phenotypes

As shown in **Table 2**, we performed 31 meta-analyses for associations between ten variants in *IL6R* gene and 13 kinds of categorical phenotypes under an additive model. Twenty significant associations were identified, which investigated ten variants associated with five kinds of phenotypes (*p* < 0.05) (**Table 2**). Specifically, the minor allele of six variants, rs7529229 (OR = 0.913, 95% CI: 0.904–0.921), rs6667434 (OR = 0.921, 95% CI: 0.897–0.946), rs4845371 (OR = 0.921, 95% CI: 0.897–0.946), rs4537545 (OR = 0.916, 95% CI: 0.882–0.952), rs4129267 (OR = 0.915, 95% CI: 0.903–0.927), and rs2228145 (OR = 0.909, 95% CI: 0.893–0.925), was significantly associated with decreased CRP level while rs4845625 (OR = 1.088, 95% CI: 1.058–1.117) was positively associated with CRP level. Similarly, the minor allele of four variants, rs7518199 (OR = 0.953, 95% CI: 0.936–0.971), rs4537545 (OR = 0.949, 95% CI: 0.933–0.966), rs4129267 (OR = 0.951, 95% CI: 0.933–0.969), and rs2228145 (OR = 0.960, 95% CI: 0.931–0.99), was also significantly associated with decreased fibrinogen level. Furthermore, the minor allele of five polymorphisms, rs7529229 (OR = 1.089, 95% CI: 1.076–1.102), rs7518199 (OR = 1.090, 95% CI: 1.061–1.121), rs4537545 (OR = 1.098, 95% CI: 1.071–1.126), rs4129267 (OR = 1.093, 95% CI: 1.074–1.113), and rs2228145 (OR = 1.130, 95% CI: 1.088–1.174), was associated with increased IL-6 level, whereas two variants rs4845618 (OR = 0.941, 95% CI: 0.927–0.955) and rs4553185 (OR = 0.936, 95% CI: 0.919–0.953) were associated with decreased IL-6 level. In addition, the minor allele of rs4129267 (OR = 1.271, 95% CI: 1.061–1.523) was positively associated with IL-6R level and rs2228145 (OR = 1.346, 95% CI: 1.318–1.374) significantly increased sIL-6R level.

**Table 2:** Variants in the *IL6R* gene associated with relevant categorical phenotypes in meta-analysis.

Variant	Allele^a^	MAF	Phenotypes	Datasets	Numbers	Risk of categorical phenotypes		Heterogeneity		Venice criteria grade	FPRP	Cumulative evidence of association
						OR (95% CI)	*p*	*І* ^2^	*p*			
rs7529229	T/C	0.3535	CRP level	5	86,998	0.913 (0.904, 0.921)	2.58×10^-81^	0.0%	0.779	AAA	<0.001	Strong
			IL-6 level	3	38,194	1.089 (1.076, 1.102)	3.88×10^-46^	39.7%	0.191	AAA	<0.001	Strong
			Fibrinogen level	8	82,958	0.976 (0.947, 1.006)	0.121	90.1%	<0.001			
rs7518199	A/C	0.3600	Fibrinogen level	6	23,634	0.953 (0.936, 0.971)	2.75×10^-7^	35.5%	–	AAA	<0.001	Strong
			IL-6 level	3	12,546	1.090 (1.061, 1.121)	8.79×10^-10^	80.0%	0.007	AAA	<0.001	Strong
rs6667434	G/A	0.4293	CRP level	4	10,471	0.921 (0.897, 0.946)	1.06×10^-9^	0.0%	0.967	AAA	<0.001	Strong
rs4845371	C/T	0.4293	CRP level	4	10,471	0.921 (0.897, 0.946)	1.06×10^-9^	0.0%	0.967	AAA	<0.001	Strong
rs4845625	C/T	0.4495	CRP level	3	8,724	1.088 (1.058, 1.117)	1.45×10^-9^	0.0%	0.371	AAC	<0.001	Moderate
rs4845618	T/G	0.4394	IL-6 level	3	12,546	0.941 (0.927, 0.955)	8.98×10^-16^	31.4%	0.233	AAA	<0.001	Strong
rs4553185	T/C	0.4343	IL-6 level	3	12,546	0.936 (0.919, 0.953)	6.04×10^-4^	52.5%	0.122	AAA	<0.001	Strong
rs4537545	C/T	0.3535	CRP level	8	103,289	0.916 (0.882, 0.952)	9.00×10^-6^	91.8%	<0.001	AAA	<0.001	Strong
			Fibrinogen level	7	41,320	0.949 (0.933, 0.966)	3.03×10^-9^	0.0%	0.409	AAA	<0.001	Strong
			IL-6 level	3	12,546	1.098 (1.071, 1.126)	3.10×10^-13^	76.0%	0.015	AAA	<0.001	Strong
			LDL cholesterol level	4	16,251	1.080 (0.728, 1.603)	0.702	0.0%	0.862			
			Total cholesterol level	4	16,251	0.977 (0.694, 1.375)	0.893	0.0%	0.887			
			Triglyceride level	3	15,256	0.973 (0.879, 1.077)	0.601	28.1%	0.249			
rs4129267	C/T	0.3485	CRP level	14	358,529	0.915 (0.903, 0.927)	6.71×10^-43^	59.1%	0.003	AAA	<0.001	Strong
			Fibrinogen level	7	41,320	0.951 (0.933, 0.969)	1.70×10^-7^	6.1%	0.302	AAA	<0.001	Strong
			IL-6 level	3	14,271	1.093 (1.074, 1.113)	7.97×10^-18^	48.3%	0.145	ABA	<0.001	Strong
			IL-6R level	3	911	1.271 (1.061, 1.523)	0.009	95.0%	<0.001	BCC	0.4000	Weak
rs2228145	A/C	0.3485	CRP level	14	483,500	0.909 (0.893, 0.925)	3.32×10^-26^	68.6%	<0.001	AAA	<0.001	Strong
			sIL-6R level	6	8,149	1.346 (1.318, 1.374)	4.66×10^-171^	54.6%	0.051	ABA	<0.001	Strong
			IL-6 level	5	42,267	1.130 (1.088, 1.174)	3.17×10^-10^	89.6%	<0.001	AAA	<0.001	Strong
			Fibrinogen level	9	98,330	0.960 (0.931, 0.990)	0.009	87.8%	<0.001	AAA	0.150	Moderate
			LDL cholesterol level	3	81,202	1.000 (0.996, 1.003)	0.857	0.0%	0.708			
			HDL cholesterol level	5	97,754	1.076 (0.860, 1.347)	0.520	62.1%	0.032			
			Triglyceride level	5	96,257	1.067 (0.855, 1.332)	0.568	59.0%	0.045			
			Systolic blood pressure	3	100,502	0.894 (0.713, 1.120)	0.329	44.6%	0.165			
			Fasting glucose	4	185,044	1.000 (0.998, 1.003)	0.799	0.0%	0.668			
			Waist circumference	3	69,772	1.002 (0.998, 1.005)	0.283	46.7%	0.131			
			BMI	4	895,213	1.001 (0.998, 1.004)	0.383	40.7%	0.168			

Venice criteria grade was determined by the amount of evidence, replication, and protection from bias. ^a^Major allele/Minor allele. CRP, C-reactive protein; IL-6, interleukin 6; IL-6R, interleukin 6 receptor; sIL-6R, soluble IL-6R.

We also conducted meta-analyses for rs2228145 and 12 continuous phenotypes under a co-dominant model (**Supplementary Table 4**). The results showed that rs2228145 was significantly associated with the levels of IL-6 and CRP.

#### 3.2.3 Heterogeneity, Sensitivity Analysis, and Bias

As shown in **Tables 1** and **2**, 24 associations revealed no or little heterogeneity (*I* ^2^ < 25%), ten associations revealed moderate heterogeneity (25% > *I* ^2^ <50%) and 24 associations revealed strong heterogeneity (*I* ^2^ > 50%). The results indicated that publication bias was found in three associations (*p* < 0.10), and a small study bias was found in nine associations (*p* < 0.10) (**Supplementary Table 5**). In addition, sensitivity analysis suggested that most associations did not significantly change when a single or first positive study, small studies, or studies with deviation from HWE in controls were excluded (data not shown).

### 3.3 Cumulative Evidence of Significant Associations

Using Venice criteria and FPRP tests, we graded cumulative evidence level for the 41 significant associations, which evaluated associations between 11 variants in the *IL6R* gene and 14 diseases and five phenotypes (**Tables 1**, **2** and **Supplementary Table 5**). According to Venice criteria, strong, moderate, and weak evidence were assigned to 29, four, and 12 associations, respectively. The FPRP test suggested that 34 of them had an FPRP value less than 0.05, two associations had an FPRP value between 0.05 and 0.20, and five associations had an FPRP value greater than 0.20. Combining Venice Criteria and FPRP results, strong, moderate, and weak evidence were finally assigned to 29, 8, and four associations, respectively. The 29 strong associations evaluated ten polymorphisms related to nine diseases and four phenotypes.

### 3.4 Phenome-Wide Profile

Linkage disequilibrium analysis (**Supplementary Table 6**) showed that five variants (rs2228145, rs7518199, rs4537545, rs7529229, and rs4129267) were highly correlated with each other, while another five SNPs (rs4845625, rs4845618, rs4845371, rs6667434, and rs4553185) were also highly correlated with each other according to *r* ^2^ (*r* ^2^ > 0.8) in the European population.

**Figure 2:**
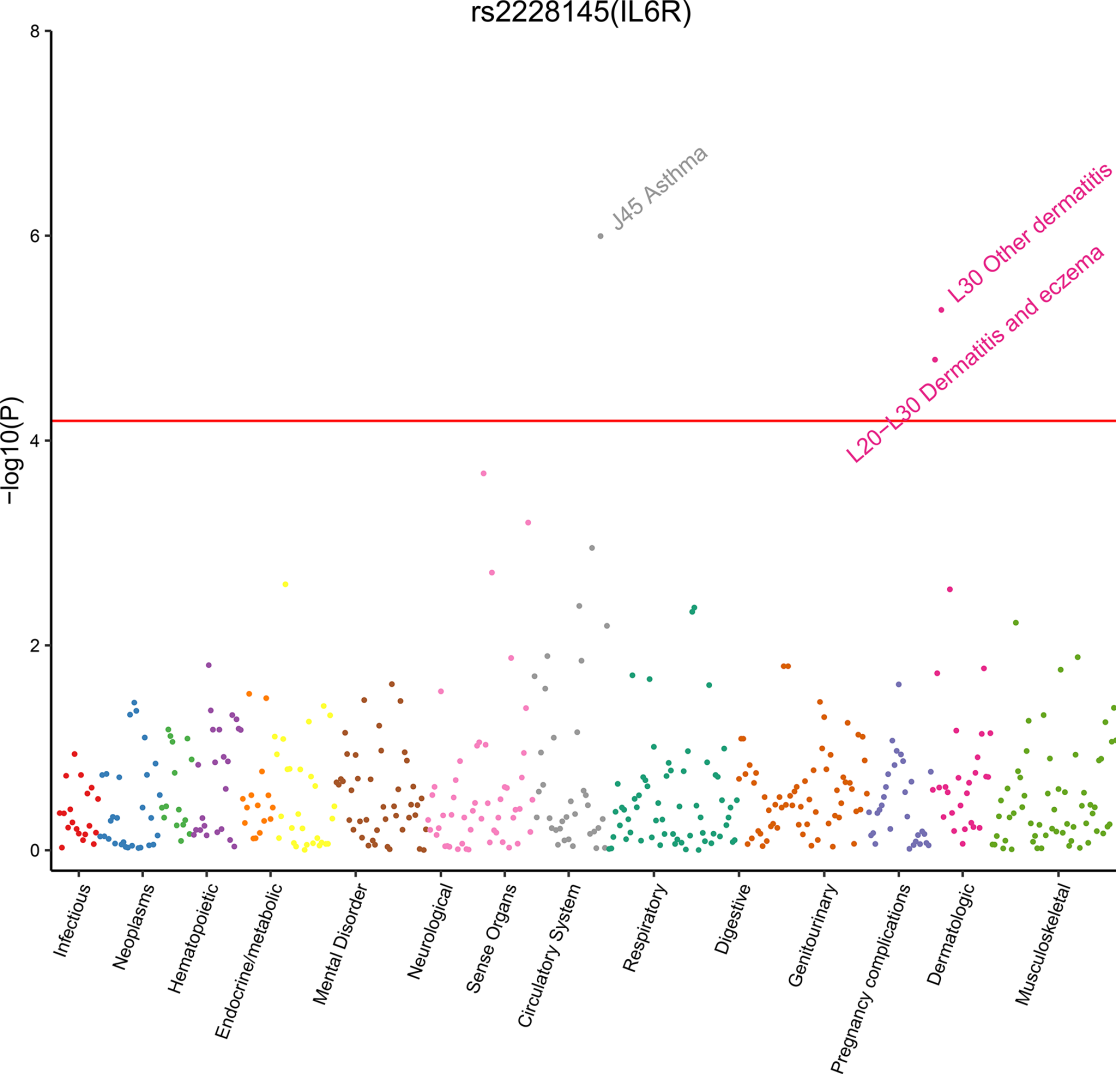
Plot of phenome-wide analysis for rs2228145 using data from UB Biobank. We estimated rs2228145 associated with 778 phenotypes in UK Biobank cohort. Phenotypes are grouped along the *x*-axis by categorization within the PheWAS code hierarchy. We considered a *p*-value < 6.43 × 10^−5^ (the red line) as significant.

Therefore, phenome-wide association analysis was just conducted for two SNPs (rs2228145 and rs4845625) using data from UK Biobank. The results are shown in **Table 3** and **Figure 2**. Variant rs2228145 and its highly correlated SNPs were found to be significantly associated with three binary phenotypes (asthma, eczema/dermatitis, and other dermatitis) and 11 non-binary phenotypes (monocyte percentage, monocyte count, mean corpuscular hemoglobin, mean corpuscular volume, mean platelet volume, platelet count, red blood cell distribution width, hemoglobin concentration, platelet distribution width, lymphocyte count, and mean sphered cell volume) at *p* < 6.43 × 10^-6^. Variant rs4845625 and its highly correlated SNPs were significantly associated with four non-binary phenotypes of mean platelet (thrombocyte) volume, platelet count, mean corpuscular hemoglobin, and monocyte count.

**Table 3:** Significant associations in phenome-wide analysis of the two independent variants using data from UK Biobank.

SNP	Position	Allele^a^	MAF	Phenotype (ICD10 code)	UK Biobank (*n* = 452,264)		
					Cases	OR (95% CI)	*p*
rs2228145	154426970	A/C	0.4094	Asthma (J45)	28,628	1.003 (1.002, 1.004)	1.29×10^-6^
				Eczema/dermatitis	11,552	1.002 (1.001, 1.003)	7.96×10^-9^
				Other dermatitis (L30)	1,654	1.001 (1.000, 1.001)	5.26×10^-6^
				Monocyte percentage	14,307	1.040 (1.032, 1.048)	1.09×10^-24^
				Monocyte count	14,322	1.003 (1.002, 1.003)	2.66×10^-19^
				Mean corpuscular hemoglobin	13,433	1.024 (1.018, 1.030)	2.19×10^-16^
				Mean corpuscular volume	13,219	1.060 (1.045, 1.075)	1.07×10^-15^
				Mean platelet (thrombocyte) volume	13,220	0.988 (0.985, 0.991)	9.81×10^-14^
				Platelet count	13,258	1.761 (1.467, 2.113)	1.21×10^-9^
				Red blood cell (erythrocyte) distribution width	13,429	0.991 (0.988, 0.994)	1.30×10^-7^
				Hemoglobin concentration	13,226	1.009 (1.005, 1.012)	4.85×10^-7^
				Platelet distribution width	13,220	0.996 (0.994, 0.998)	2.10×10^-6^
				Lymphocyte count	14,209	0.995 (0.993, 0.997)	4.39×10^-6^
				Mean sphered cell volume	20,355	0.961 (0.945, 0.978)	6.01×10^-6^
rs4845625	154422067	C/T	0.4221	Mean platelet (thrombocyte) volume	13,220	1.011 (1.008, 1.015)	2.79×10^-12^
				Platelet count	13,258	0.570 (0.475, 0.685)	1.62×10^-9^
				Mean corpuscular hemoglobin	13,433	0.986 (0.980, 0.992)	1.63×10^-6^
				Monocyte count	14,322	0.999 (0.998, 0.999)	6.08×10^-6^

MAF in UB Biobank. ^a^Major allele/Minor allele.

### 3.5 Functional Annotation of Variants With Strong Evidence

Bioinformatics analyses suggested that the ten variants with strong evidence and variants highly correlated with them might fall within an exon, a DNase I hypersensitivity site, a strong prompter, and an enhancer activity region (**Table 4** and **Supplementary Figure 1**). Of those SNPs, rs2228145 is a nonsynonymous variant located in exon 9 of the *IL6R* gene. In variant rs2228145, A to C transition is observed at position 358 in the *IL6R* gene, resulting in the alteration of amino acid from alanine to aspartate or valine. The C allele of rs2228145 is associated with inflammatory/autoimmune diseases as well as quantitative traits (7, 9), and the mechanisms might be involved in regulating the balance of transmembrane IL-6R (mIL-6R) and sIL-6R concentrations and responding to IL-6 (12). Additionally, rs2228145 is in LD with four intronic SNPs (rs4129267, rs4537545, rs7529229, and rs7518199) in Europeans (*r* ^2^ > 0.85 for all tests), indicating similar functional mechanisms in this ethnic group. According to LD analysis in the European population, cis-eQTL analysis was conducted for rs2228145 and rs4845625 (**Supplementary Table 7**). The findings indicate that rs2228145 and rs4845625 are associated with a decrease in IL-6R in an artery or heart tissue. In addition, rs2228145 is an eQTL for *SHE*, *TDRD10*, and *UBE2Q1* genes, and rs4845625 is an eQTL for *PSMD8P1*, *TDRD10*, *UBAP2L*, and *AQP10* genes.

**Table 4:** Functional annotation of the ten variants showing strong evidence using data from the ENCODE project.

SNP	Position^a^	Allele^b^	RAF in CEU/ASN/AFR	Annotation	Promoter histone marks^c^	Enhancer histone marks^d^	DNase^e^	Proteins bound^f^	Motifs changed^g^
rs2228145	154454494	A/C,T	0.36/0.36/0.09	Missense	5 tissues	16 tissues			
rs4129267	154453788	C/T	0.36/0.36/0.09	Intronic	8 tissues	20 tissues	24 tissues	STAT3, KAP1	EBF, Elf3, Pou1f1
rs4537545	154446403	C/T	0.37/0.35/0.68	Intronic	4 tissues	16 tissues	18 tissues	MAFK	
rs7529229	154448302	T/C	0.37/0.36/0.69	Intronic	GI	10 tissues	MUS		ZEB1
rs4553185	154438479	C/T	0.54/0.46/0.46	Intronic	BLD, SKIN, GI	12 tissues			Pax-6, Pou3f4, RORalpha1
rs4845371	154435864	T/C	0.55/0.46/0.68	Intronic	4 tissues	17 tissues	MUS		4 altered motifs
rs4845618	154427539	G/T	0.53/0.48/0.48	Intronic	8 tissues	23 tissues	5 tissues		HDAC2, Irf, STAT
rs4845625	154449591	T/C	0.54/0.47/0.72	Intronic		8 tissues	PLCNT		Pax-2, Pax-5
rs6667434	154436624	A/G	0.55/0.46/0.67	Intronic	ESDR, SKIN, GI	13 tissues	4 tissues		BCL, NRSF
rs7518199	154434943	A/C	0.36/0.37/0.2	Intronic	10 tissues	19 tissues	10 tissues		NRSF

a The chromosome position (bp) is based on NCBI, Build 38.

b Reference allele/Risk allele.

c Evidence of local H3K4Me1 and H3K27Ac modification (cell lines/types: if >3, only the number is included).

d Evidence of local H3K4Me3 modification (cell lines/types: if >3, only the number is included).

e Evidence of chromatin hypersensitivity to DNase (cell lines/types: if >3, only the number is included).

f ChIP-seq experiments indicate alteration in binding of transcription factor (if >3, only the number is included).

g Evidence of alteration in regulatory motif (if >3, only the number is included).

RAF, risk allele frequency; CEU, Utah residents with Northern and Western European ancestry from the CEPH collection; ASN, Asian; AFR, African.

## 4 Discussion

Our study summarizes the published data thus far on relationships between variants in the *IL6R* gene and the risk of human diseases and phenotypes. The meta-analyses results show that 12 variants in the *IL6R* gene are significantly associated with risk of CVDs (CAD, coronary heart disease, atrial fibrillation, aneurysms, and atherosclerosis), inflammatory or autoimmune diseases (rheumatoid arthritis, asthma, Crohn’s disease, ulcerative colitis, dermatitis, and type 1 diabetes), and levels of inflammatory biomarkers (CRP, fibrinogen, IL-6, IL-6R, and sIL-6R). We then assigned strong cumulative evidence to 29 significant associations, which assessed ten variants in this gene related to the risk of five cardiovascular diseases, four inflammatory diseases, and the concentration of four phenotypes. Further analysis of functional annotation indicates that the strong variants might fall in putative functional regions.

*IL6R* gene encodes a subunit of an IL-6R protein complex that exists in two forms, an mIL-6R and sIL-6R, resulting in different signal transduction mechanisms known as IL-6/IL-6R classic signaling and trans-signaling, respectively. These two models of signaling can mediate the anti-inflammatory (classic signaling) and pro-inflammatory (trans-signaling) response of IL-6 (37, 38). By specifically binding to IL-6, IL-6/IL-6R signaling plays an essential role in the pathogenesis of many diseases, including COVID-2019 (4, 7, 9, 10).

Previous studies have suggested that rs2228145 has similar synergistic effects as tocilizumab on a series of cardiovascular and inflammatory biomarkers (10, 12, 19). Consistent with this, our meta-analysis found that this variant was significantly related to a decreased serum concentration of CRP and fibrinogen. There were also consistent associations with strong cumulative evidence between rs2228145 and a reduced risk of CHD, atherosclerosis, AAA, rheumatoid arthritis, and inflammatory bowel disease identified by this meta-analysis, findings that suggest targeting the IL-6R is a plausible strategy in cardiovascular and inflammatory diseases. On the contrary, the C allele of rs2228145 is strongly related to higher levels of sIL-6R and IL-6, which seems paradoxical. This apparent paradox might be interpreted that the C allele reduces mIL-6R level either by alternative splicing or increasing the membrane-bound form shedding rather than by regulating the production of IL-6R or IL-6 (9). Such mechanisms could account for the accumulation of both sIL-6R and IL-6 in the circulation of individuals carrying the C allele of rs2228145.

The mutation of rs2228145 promotes the proteolysis of the IL-6R, which increases the level of sIL-6R and decreases the level of mIL-6R (39). Thus, this variant exerts its functional mechanism by regulating the balance between mIL-6R and sIL-6R (12). The minor allele of rs2228145 strongly increases the concentration of sIL-6R, whereas it reduces the surface expression of mIL-6R in individual immune cells. Reducing mIL-6R expression leads to impaired IL-6 responsiveness, as measured by decreased phosphorylation of the transcription factors STAT3 and STAT1 following stimulation with IL-6 (12, 19). Therefore, it is speculated that rs2228145 impairs classical IL-6/IL-6R signaling and reduces the inflammatory response, thus leading to a lower risk of CVDs, inflammatory, or autoimmune diseases (19). Our findings also link rs2228145 with an increased risk of asthma and dermatitis, but the exact mechanisms for these associations remain unclear. Experimental evidence indicates that IL-6/sIL-6R trans-signaling might play an essential role in the development of asthma (40, 41) and dermatitis (42). Further studies are warranted to elucidate these mechanisms.

LD analysis using data from the 1000 Genomes Project reveals that four variants (rs7518199, rs4537545, rs7529229, and rs4129267) are highly correlated with rs2228145 in the European population (*r* ^2^ > 0.90 for all tests), indicating that the functional mechanisms of these SNPs associated with diseases and phenotypes might be similar. In addition, the variant rs4845625 (T allele) strongly associated with CAD is in weak or moderate LD with rs2228145 (*r* ^2^ = 0.04, 0.462, and 0.63 in African, European, and Asian populations, respectively), reflecting that functional mechanisms of *IL6R* variants associated with CVD risk might be distinct. Additional studies are needed to investigate rs4845625 and its related SNPs affecting the development of CVD.

Clinically, targeting the IL-6/IL-6R pathway is a plausible therapeutic strategy in several cardiovascular and inflammatory diseases, including COVID-2019 (37, 43–45). For example, clinical trials suggest that tocilizumab, a humanized monoclonal biologic drug that blocks both sIL-6R and mIL-6R, is an effective treatment for rheumatoid arthritis (44). In addition, sgp130Fc, a protein that specifically blocks trans-signaling, is an effective therapy for various preclinical chronic and autoimmune disease models (37, 42). Therefore, our findings have clinical implications and may provide tools for identifying patients who can benefit from the therapeutic intervention in the IL-6/IL-6R pathway.

Some limitations should be noted in this study. Firstly, although we have tried our best to perform a comprehensive meta-analysis to include all the relevant literature, a small part of articles may still be missed. Secondly, several studies were omitted without valid data for extraction. For example, some articles that just provide a histogram of the average level of phenotypes corresponding to different genotypes were excluded. Thirdly, meta-analysis was not performed for some diseases and phenotypes, such as breast cancer, colorectal cancer, stroke, and systemic lupus erythematosus because fewer than three datasets were included. Fourthly, we could not define a unified analytical standard across studies since we could not obtain raw data from the original studies. To minimize bias, we adopted age, sex, or multivariable-adjusted risk estimates. Fifthly, moderate and weak evidence of significant associations needs to be explained with caution. Future studies with a larger sample size are required to refute or confirm these associations.

In conclusion, our study provides comprehensive evidence that variants in the *IL6R* gene are associated with the risk of inflammatory or autoimmune diseases and levels of inflammatory biomarkers. Also, it highlights the significant role of the IL-6/IL-6R pathway in the pathogenesis of cardiovascular and inflammatory diseases. Phenome-wide association analysis confirmed that variants in the *IL6R* gene are associated with asthma and dermatitis. Bioinformatics analyses showed that most of these variants fall in putative functional regions. Those findings highlight that the variations in the IL6R gene may be considered a useful genetic tool for investigating the pharmacological targeting potential of IL-6R. More efforts should be made to understand the biological pathways further and apply these lines of evidences for clinical practice and public health for risk evaluation and management.

## Data Availability Statement

The original contributions presented in the study are included in the article/**Supplementary Material**. Further inquiries can be directed to the corresponding author.

## Ethics Statement

Ethical review and approval were not required for the study on human participants in accordance with the local legislation and institutional requirements. Written informed consent for participation was not required for this study in accordance with the national legislation and the institutional requirements.

## Author Contributions

DG initiated and designed this study and approved the final draft manuscript. MZ and YB performed study selection, data extraction, and statistical analysis. LW, HC, and MT performed the cumulative evidence assessments and phenome-wide analysis. YW and XW conducted the functional analyses. MZ also drafted the first manuscript. All authors contributed to the article and approved the submitted version.

## Funding

This study was supported by the National Natural Science Foundation of China (81903393 and 81903398). The funding agencies of this study had no role in study design, data collection, data management, data analysis, data interpretation, writing of the manuscript, or the decision for submission.

## Conflict of Interest

The authors declare that the research was conducted in the absence of any commercial or financial relationships that could be construed as a potential conflict of interest.

## Publisher’s Note

All claims expressed in this article are solely those of the authors and do not necessarily represent those of their affiliated organizations, or those of the publisher, the editors and the reviewers. Any product that may be evaluated in this article, or claim that may be made by its manufacturer, is not guaranteed or endorsed by the publisher.
