# Supplementary material for: Cumulative Evidence for Associations Between Genetic Variants in Interleukin 6 Receptor Gene and Human Diseases and Phenotypes
Source: Front Immunol. 2022 Apr 14;13:860703. doi: 10.3389/fimmu.2022.860703 (PMC9046675; doi:10.3389/fimmu.2022.860703)
Supplement: Supplementary file 1 [file DataSheet_1.docx]

**Supplementary Materials to:**

**Cumulative evidence for associations between genetic variants in interleukin 6 receptor gene and human diseases and phenotypes**

Min Zhang^1^, Ye Bai^1^, Yutong Wang^2^, Huijie Cui^2^, Mingshuang Tang^2^, Lanbing Wang^3^, Xin Wang^2^, Dongqing Gu^4*^

^1^ School of Public Health and Management, Chongqing Medical University, Chongqing 400016, China.

^2^ Department of Epidemiology and Medicine, West China School of Public Health and West China Fourth Hospital, Sichuan University, Chengdu 610041, Sichuan, China.

^3^ Division of Medical Affairs, The First Affiliated Hospital of Army Military Medical University, Chongqing 400038, China.

^4^ Division of Noncommunicable Disease Epidemiology, The First Affiliated Hospital of Army Military Medical University, Chongqing 400038, China.

^* ­^**Correspondence to:**

**Dongqing Gu**

Division of Noncommunicable Disease Epidemiology, The First Affiliated Hospital of Army Military Medical University, 30 Gaotanyan Street, Shapingba District, Chongqing 400038, China.

Email: [dongqing.gu@vip.163.com](mailto:dongqing.gu@vip.163.com)

**A list of supporting informations**

**Supplementary notes for the Vence criteria** (**Page 3**)

**Supplementary Figure 1.** Evidence from ENCODE data for regulatory function of variants in *IL-6R* gene using the UCSC Genome Browser. (**Pages 4-5**)

**Supplementary Table 1.** Characteristics of the included studies on variants in the *IL6R* gene and risk of human diseases. (**Pages 6-17**)

**Supplementary Table 2.** Characteristics of the included studies on variants in the *IL6R* gene and levels of categorical phenotypes. (**Pages 18-27**)

**Supplementary Table 3.** Characteristics of the included studies on variants in the *IL6R* gene and mean levels of continuous quantitative phenotypes. (**Pages 28-33**)

**Supplementary Table 4.** Polymorphism rs2228145 associated with continuous quantitative phenotypes in meta-analysis. (**Page 34**)

**Supplementary Table 5.** Details of protection from bias for genetic variants significantly associated with risk of diseases and phenotypes. (**Pages 35-36**)

**Supplementary Table 6.** Correlations (*r*^2^) among the ten variants showing strong evidence using data from the 1000 Genomes Project. (**Pages 37-38**)

**Supplementary Table 7.**  Analyses of expression quantitative trait locus (eQTL) in the two independent loci. (**Pages 39**)

**Supplementary notes for the Vence criteria**

We applied the Venice criteria to evaluate the epidemiological credibility of significant associations identified by meta-analysis. Briefly, credibility was defined as strong, moderate, or weak, based on the grade of A, B, or C in three categories: amount of evidence, replication of the association, and protection from bias.

**Amount of evidence**

A: Large-scale evidence — minor genetic group (alleles or genotypes) in cases and controls > 1,000. B: Moderate amount of evidence — minor genetic group in cases and controls between 100 and 1,000. C: Little evidence — minor genetic group in cases and controls < 100.

**Replication of association**

A: Little between-study heterogeneity — *I*^2^ < 25%. B: Moderate between-study heterogeneity — *I*^2^ between 25% and 50%. C: Large between-study heterogeneity — *I*^2^ > 50%. Qualitative epidemiologic considerations about the presence of heterogeneity and potential explanation for heterogeneity would need to be taken into account in judging replication. It may be reasonable to grade as A on this criterion for associations with moderate or high heterogeneity with an extensive replication record such as associations identified by GWAS or large GWAS meta-analysis from collaborative studies.

**Protection from bias**

A: No observable bias and bias was unlikely to explain the presence of the association. B: No obvious bias may affect the presence of the association, but there is considerable missing information on the identification of evidence. C: Bias is demonstrable or is likely to explain the presence of the association. The Venice criteria include an extensive checklist for sources of bias in different settings. The checklist has different considerations depending on whether the evidence comes from retrospective meta-analyses of published data or prospective GWAS and replication studies from collaborative consortia with harmonization of data collection and analysis.

General checks for bias that have been adopted for meta-analysis are: (1) Association lost with exclusion of first study; (2) Association lost with exclusion of studies deviated from HWE; (3) Small effect size of association (i.e., OR < 1.15); (4) Evidence of publication bias (*p* < 0.10 in Begg's test); (5) Evidence of small-study effect (*p* < 0.10 in Egger's test); (6) Evidence is presented for an excess of individual studies with significant findings (*p* < 0.10 in significant bias test).


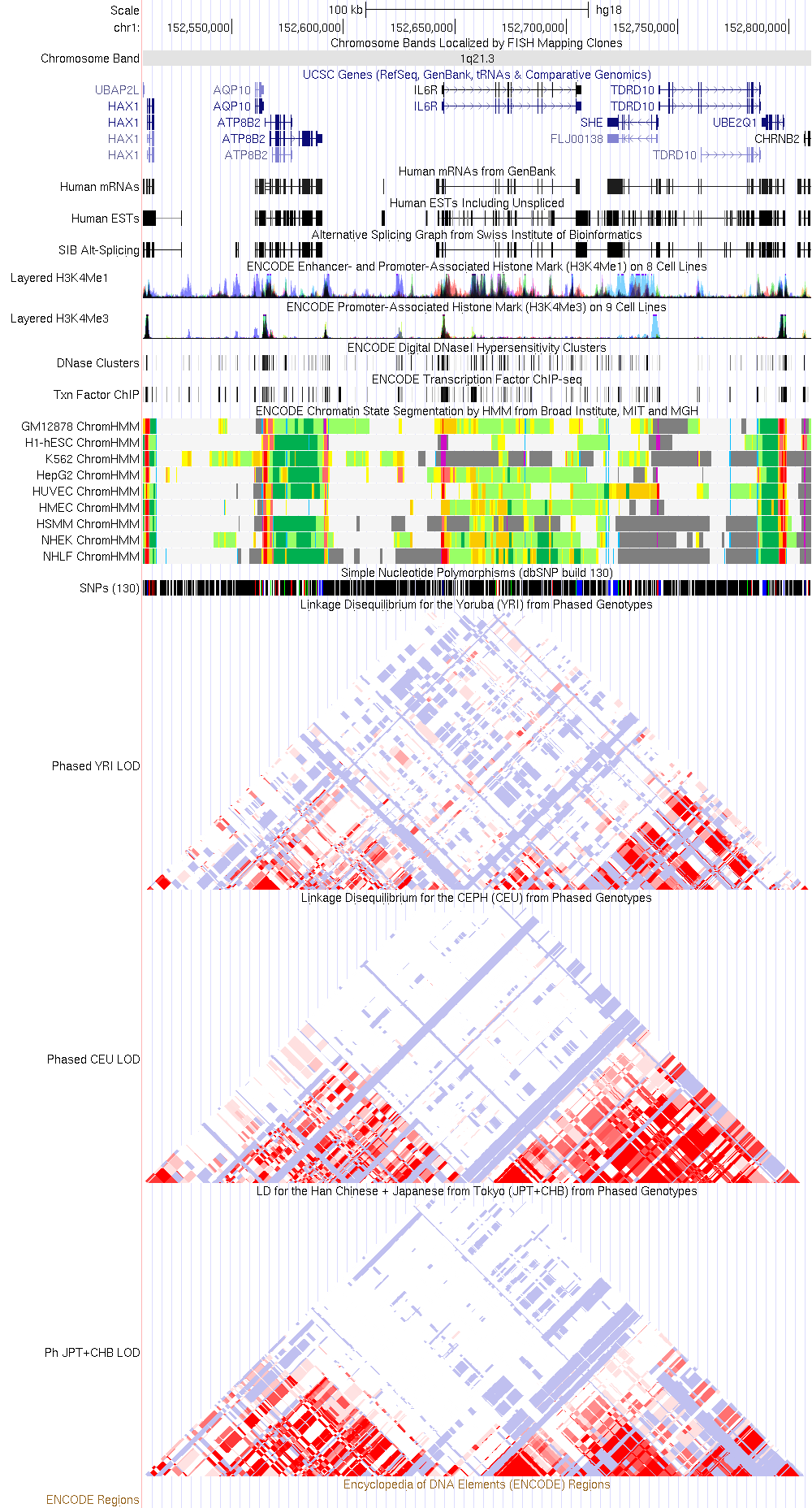


**Supplementary Figure 1.** Evidence from ENCODE data for regulatory function of variants in *IL-6R* gene using the UCSC Genome Browser. The plot represent a 1000-kb window centered on *IL6R* gene region (NCBI Human Genome GRCh37). Tracks (from top to bottom) in each of the plots are Genome Base Position, Chromosome Bands, UCSC Genes, Human mRNAs from GenBank, Human ESTs That Have Been Spliced, Alternative Splicing Graph, ENCODE Enhancer- and Promoter-Associated Histone Mark (H3K4Me1) on 8 Cell Lines, ENCODE Promoter-Associated Histone Mark (H3K4Me3) on 9 Cell Lines, ENCODE Digital DNaseI Hypersensitivity Clusters, ENCODE Transcription Factor ChIP-seq, ENCODE Chromatin State Segmentation by HMM from Broad Institute (bright red, active promoter; light red, weak promoter; purple, inactive/poised promoter; orange, strong enhancer; yellow, weak/poised enhancer; blue, insulator; dark green, transcriptional transition/elongation; light green, weak transcribed; gray, polycomb-repressed; light gray, heterochromatin/low signal/repetitive/copy number variation), Simple Nucleotide Polymorphisms (dbSNP build 130), Linkage Disequilibrium (LD) for the Yoruba (YRI) from Phased Genotypes, LD for the CEPH (CEU) from Phased Genotypes, and LD for the Han Chinese+Japanese from Tokyo (CHB+JPT) from Phased Genotypes.

**Supplementary Table 1. Characteristics of the included studies on variants in the *IL6R* gene and risk of human diseases.**

| **PMID** | **Author** | **Year** | **SNP** | **Country** | **Diseases** | **Study design** | **Ethnicity** | **Major-allele** | **Minor-allele** | **Cases** | **Controls** | **OR (95%CI)** |
| --- | --- | --- | --- | --- | --- | --- | --- | --- | --- | --- | --- | --- |
| 33912579 | Raita | 2021 | rs79778789 | UKB | asthma | MR | Caucasian | G | A | 46799 | 347457 | 1.020 (0.960, 1.090) |
| 33912579 | Raita | 2021 | rs79219014 | UKB | asthma | MR | Caucasian | T | G | 46799 | 347457 | 1.020 (0.970, 1.080) |
| 33912579 | Raita | 2021 | rs7525477 | UKB | asthma | MR | Caucasian | A | G | 46799 | 347457 | 0.980 (0.940, 1.020) |
| 33912579 | Raita | 2021 | rs4129267 | UKB | asthma | MR | Caucasian | C | T | 46799 | 347457 | 1.030 (1.020, 1.040) |
| 33912579 | Raita | 2021 | rs145262901 | UKB | asthma | MR | Caucasian | A | G | 46799 | 347457 | 0.930 (0.840, 1.020) |
| 33912579 | Raita | 2021 | rs139952834 | UKB | asthma | MR | Caucasian | T | C | 46799 | 347457 | 0.970 (0.880, 1.070) |
| 33912579 | Raita | 2021 | rs116141616 | UKB | asthma | MR | Caucasian | A | G | 46799 | 347457 | 1.070 (0.970, 1.170) |
| 33912579 | Raita | 2021 | rs116037345 | UKB | asthma | MR | Caucasian | T | C | 46799 | 347457 | 0.990 (0.920, 1.060) |
| 33912579 | Raita | 2021 | rs113580743 | UKB | asthma | MR | Caucasian | A | G | 46799 | 347457 | 0.940 (0.880, 1.010) |
| 33536774 | Sokolik | 2021 | rs2228145 | Russia | psoriatic arthritis | case-control study | Caucasian | A | C | 74 | 120 | 1.146 (0.751, 1.749) |
| 32920385 | Ramos | 2021 | rs2228145 | Poland | exposed to pesticides | case-control study | Other | A | C | 180 | 180 | 1.046 (0.780, 1.401) |
| 32561314 | Wu | 2021 | rs2228145 | European | Postoperative atrial fibrillation | case-control study | Asian | A | C | 112 | 259 | 1.544 (1.122, 2.123) |
| 33659786 | Topchieva | 2020 | rs2228145 | UKB | Hypertension | case-control study | Caucasian | A | C | 152 | 148 | 1.091 (0.783, 1.522) |
| 33096487 | Bowker | 2020 | rs2228145 | European | type 2 diabetes | GWAS | Caucasian | A | C | 260614 | 1350640 | 0.980 (0.970, 0.990) |
| 33096487 | Bowker | 2020 | rs2228145 | England | coronary artery disease | case-control study | Caucasian | A | C | 24890 | 427309 | 0.960 (0.950, 0.980) |
| 33096487 | Bowker | 2020 | rs2228145 | England | type 1 diabetes | GWAS | Caucasian | A | C | 24209 | 758240 | 0.970 (0.950, 0.990) |
| 33015641 | Bovijn | 2020 | rs2228145 | Brazil | hospitalisation for COVID-19 | MR | Caucasian | A | C | 928 | 2028 | 0.830 (0.700, 0.980) |
| 33015641 | Bovijn | 2020 | rs2228145 | China | hospitalisation for COVID-19 | MR | Caucasian | A | C | 3199 | 897488 | 0.940 (0.870, 1.010) |
| 32535289 | Usategui-Martín | 2020 | rs2228145 | China | osteoporotic fracture | case-control study | Asian | A | C | 221 | 354 | 1.580 (1.235, 2.022) |
| 32382712 | Pedersen | 2020 | rs4537545 | Danes | JAK2V617F somatic mutation | cohort study-MR | Caucasian | C | T | 62 | 49143 | 0.660 (0.450, 0.960) |
| 32382712 | Pedersen | 2020 | rs4537545 | Danes | myeloproliferative neoplasm | cohort study-MR | Caucasian | C | T | 352 | 107969 | 0.810 (0.700, 0.940) |
| 32328834 | Zhang | 2020 | rs2228145 | Mix | Multiple sclerosis | case-control study | Caucasian | A | C | 32367 | 36012 | 0.988 (0.982, 0.994) |
| 32328834 | Zhang | 2020 | rs2228145 | Mix | Alzheimer’s disease | case-control study | Asian | A | C | 21982 | 41944 | 1.003 (0.975, 1.032) |
| 32328834 | Zhang | 2020 | rs2228145 | Mix | Parkinson’s disease | case-control study | Asian | A | C | 33674 | 449056 | 0.981 (0.949, 1.015) |
| 32328834 | Zhang | 2020 | rs2228145 | Mix | amyotrophic lateral sclerosis | case-control study | Asian | A | C | 20806 | 59804 | 1.001 (0.974, 1.028) |
| 32292581 | Ciesla | 2020 | rs4129267 | Poland | rheumatoid arthritis | case-control study | Caucasian | C | T | 122 | 24 | 0.789 (0.407, 1.528) |
| 32292581 | Ciesla | 2020 | rs2228145 | Poland | rheumatoid arthritis | case-control study | Caucasian | A | C | 122 | 24 | 0.903 (0.462, 1.764) |
| 32223966 | Yuan | 2020 | rs4129267 | European | coronary artery disease | case-control study | Caucasian | C | T | 60801 | 123504 | 0.952 (0.934, 0.971) |
| 32223966 | Yuan | 2020 | rs4129267 | European | atrial fibrillation | case-control study | Caucasian | C | T | 55114 | 482295 | 0.962 (0.949, 0.975) |
| 32223966 | Yuan | 2020 | rs4129267 | European | ischemic stroke | case-control study | Caucasian | C | T | 34217 | 404603 | 0.977 (0.958, 0.997) |
| 31929778 | Alkharsah | 2019 | rs2228145 | Saudi Arabia | HCV infection | case-control study | Asian | A | C | 17 | 132 | 1.327 (0.634, 2.776) |
| 31918059 | Wei | 2020 | rs4845626 | China | lung cancer | case-control study | Asian | G | T | 550 | 550 | 0.640 (0.540, 0.790) |
| 31918059 | Wei | 2020 | rs4329505 | China | lung cancer | case-control study | Asian | T | C | 550 | 550 | 0.750 (0.630, 0.900) |
| 31900081 | Lin | 2020 | rs2228145 | European | ischemic stroke | case-control study | Other | A | C | 34217 | 404603 | 0.978 (0.958, 0.998) |
| 31744360 | Andrade Ramos | 2019 | rs2228145 | Brazil | Minor Neonatal morbidity | case-control study | Other | A | C | 91 | 181 | 0.460 (0.220, 0.980) |
| 31744360 | Andrade Ramos | 2019 | rs2228145 | Brazil | Major Infant morbidity | case-control study | Asian | A | C | 91 | 181 | 0.310 (0.110, 0.870) |
| 31707836 | Bick | 2020 | rs2228145 | England | Cardiovascular disease | cohort study | Caucasian | A | C | 53 | 432 | 0.460 (0.290, 0.730) |
| 31707836 | Bick | 2020 | rs2228145 | England | Cardiovascular disease | cohort study | Caucasian | A | C | 1951 | 34337 | 0.950 (0.890, 1.010) |
| 31679401 | Huang | 2020 | rs2228145 | China | large artery atherosclerotic stroke | cohort study | Asian | A | C | 768 | 686 | 0.722 (0.619, 0.843) |
| 31679401 | Huang | 2020 | rs2228145 | China | outcome of atherosclerotic stroke | cohort study | Asian | A | C | 768 | 686 | 0.640 (0.500, 0.820) |
| 31668584 | Cornish | 2020 | rs4129267 | Mix | Colorectal cancer | cohort study-MR | Caucasian | C | T | 7952 | 11680 | 0.974 (0.950, 0.998) |
| 31665421 | Saunders | 2020 | rs4129267 | Mix | glioma | cohort study-MR | Caucasian | C | T | 12488 | 18169 | 0.992 (0.957, 1.028) |
| 31468132 | Kaanane | 2019 | rs2228145 | Moroccan | lung cancer | case-control study | Caucasian | A | C | 120 | 120 | 0.800 (0.490, 1.310) |
| 31395468 | Suijkerbuijk | 2019 | rs2228145 | Sweden | anterior cruciate ligament injury | case-control study | Caucasian | A | C | 79 | 116 | 1.089 (0.711, 1.668) |
| 31395468 | Suijkerbuijk | 2019 | rs2228145 | South Africa | anterior cruciate ligament injury | case-control study | Caucasian | A | C | 98 | 100 | 0.687 (0.439, 1.076) |
| 31341681 | Cavieres | 2019 | rs2228145 | Chilean | treatment for Schizophrenia | case-control study | Caucasian | A | C | 24 | 48 | 0.738 (0.364, 1.494) |
| 31341681 | Cavieres | 2019 | rs2228145 | Chilean | treatment for Schizophrenia | case-control study | Caucasian | A | C | 41 | 48 | 0.944 (0.513, 1.737) |
| 31312381 | He | 2019 | rs2228145 | China | gastric cancer | case-control study | Asian | A | C | 479 | 483 | 0.940 (0.790, 1.130) |
| 31219249 | Song | 2019 | rs7553796 | China | HCV infection | case-control study | Asian | C | A | 394 | 395 | 0.587 (0.481, 0.717) |
| 31219249 | Song | 2019 | rs4845617 | China | HCV infection | case-control study | Asian | G | A | 394 | 395 | 0.950 (0.779, 1.158) |
| 31219249 | Song | 2019 | rs4845374 | China | HCV infection | case-control study | Asian | T | A | 394 | 395 | 1.259 (0.955, 1.660) |
| 31219249 | Song | 2019 | rs4509570 | China | HCV infection | case-control study | Asian | C | G | 394 | 395 | 1.314 (0.972, 1.776) |
| 31219249 | Song | 2019 | rs4075015 | China | HCV infection | case-control study | Asian | A | T | 394 | 395 | 0.950 (0.777, 1.161) |
| 31219249 | Song | 2019 | rs12090237 | China | HCV infection | case-control study | Asian | G | A | 394 | 395 | 0.913 (0.559, 1.490) |
| 31100612 | Draganov | 2019 | rs6690230 | Spain | treatment response in depression | cross-sectional | Caucasian | G | C | 91 | 62 | 1.200 (0.706, 2.038) |
| 31100612 | Draganov | 2019 | rs57569414 | Spain | treatment response in depression | cross-sectional | Caucasian | C | A | 91 | 62 | 0.350 (0.160, 0.770) |
| 31100612 | Draganov | 2019 | rs4556347 | Spain | treatment response in depression | cross-sectional | Caucasian | T | A | 91 | 62 | 0.78 (0.463, 1.313) |
| 31100612 | Draganov | 2019 | rs4133213 | Spain | treatment response in depression | cross-sectional | Caucasian | A | G | 91 | 62 | 0.93 (0.559, 1.548) |
| 31100612 | Draganov | 2019 | rs4075015 | Spain | treatment response in depression | cross-sectional | Caucasian | A | T | 91 | 62 | 1.64 (1.02, 2.65) |
| 31100612 | Draganov | 2019 | rs3887104 | Spain | treatment response in depression | cross-sectional | Caucasian | C | T | 91 | 62 | 1.23 (0.683, 2.214) |
| 31100612 | Draganov | 2019 | rs12083537 | Spain | treatment response in depression | cross-sectional | Caucasian | T | C | 91 | 62 | 0.77 (0.402, 1.474) |
| 31100612 | Draganov | 2019 | rs12047973 | Spain | treatment response in depression | cross-sectional | Caucasian | A | G | 91 | 62 | 0.99 (0.206, 4.767) |
| 31021550 | Avendano-Tamayo | 2019 | rs2228145 | Colombian | dengue | case-control study | Caucasian | A | C | 191 | 101 | 0.425 (0.204, 0.887) |
| 31021550 | Avendano-Tamayo | 2019 | rs2228145 | Colombian | dengue | case-control study | Caucasian | A | C | 191 | 101 | 1.498 (0.856, 2.62) |
| 30787661 | Chycki | 2019 | rs2228145 | Poland | Anterior Cruciate Ligament Rupture | case-control study | Caucasian | A | C | 229 | 195 | 1.007 (0.76, 1.334) |
| 30662970 | Sundaresh | 2019 | rs2228145 | French | bipolar disorder | case-control study | Caucasian | A | C | 565 | 201 | 1.084 (0.856, 1.371) |
| 30662970 | Sundaresh | 2019 | rs2228145 | South Indian Tamil | bipolar disorder | case-control study | Caucasian | A | C | 152 | 159 | 0.995 (0.696, 1.421) |
| 30474404 | Kunisato | 2019 | rs4537545 | Japanese | autoimmune thyroid disease | case-control study | Asian | C | T | 262 | 67 | 0.89 (0.61, 1.31) |
| 30474404 | Kunisato | 2019 | rs4537545 | Japanese | Graves’ disease | case-control study | Asian | C | T | 133 | 67 | 0.93 (0.61, 1.41) |
| 30474404 | Kunisato | 2019 | rs4537545 | Japanese | Hashimoto’s disease | case-control study | Asian | C | T | 130 | 67 | 0.88 (0.58, 1.34) |
| 30445015 | Liu | 2019 | rs2228145 | Mix | Crohn’s disease | case-control study | Caucasian | A | C | 12194 | 28072 | 0.942 (0.912, 0.972) |
| 30445015 | Liu | 2019 | rs2228145 | Mix | ulcerative colitis | case-control study | Caucasian | A | C | 12366 | 28072 | 0.981 (0.95, 1.012) |
| 30249138 | Preda | 2018 | rs8192282 | Romania | Spontaneous preterm birth maternal | case-control study | Caucasian | A | G | 79 | 81 | 1.168 (0.695, 1.963) |
| 30249138 | Preda | 2018 | rs8192282 | Romania | Spontaneous preterm birth fetal | case-control study | Caucasian | A | G | 79 | 81 | 0.958 (0.561, 1.637) |
| 30228077 | Useche | 2018 | rs2228145 | Huila,Colombian | dengue | case-control study | Caucasian | A | C | 176 | 311 | 0.602 (0.391, 0.935) |
| 30228077 | Useche | 2018 | rs2228145 | Antioquia,Colombian | dengue | case-control study | Caucasian | A | C | 122 | 337 | 1.163 (0.714, 1.923) |
| 30090940 | Cai | 2018 | rs2228145 | USA | Abdominal aortic aneurysm | case-control study | Caucasian | A | C | 7987 | 324812 | 0.87 (0.84, 0.9) |
| 30090940 | Cai | 2018 | rs2228145 | USA | aneurysm | case-control study | Caucasian | A | C | 9984 | 322815 | 0.9 (0.87, 0.93) |
| 30090940 | Cai | 2018 | rs2228145 | USA | aneurysm | case-control study | Other | A | C | 11315 | 321484 | 0.92 (0.89, 0.94) |
| 30090940 | Cai | 2018 | rs2228145 | USA | Aneurysm | cohort study-MR | Caucasian | A | C | 998 | 331801 | 0.83 (0.75, 0.92) |
| 30090940 | Cai | 2018 | rs2228145 | USA | atherosclerosis | case-control study | Other | A | C | 68224 | 264575 | 0.95 (0.94, 0.97) |
| 30090940 | Cai | 2018 | rs2228145 | USA | Ischemic heart disease | case-control study | Other | A | C | 84531 | 248268 | 0.95 (0.94, 0.97) |
| 30090940 | Cai | 2018 | rs2228145 | USA | ischemic heart disease | case-control study | Caucasian | A | C | 49587 | 283212 | 0.95 (0.93, 0.97) |
| 30090940 | Cai | 2018 | rs2228145 | USA | Myocardial infarction | case-control study | African | A | C | 18304 | 314495 | 0.94 (0.92, 0.97) |
| 30090940 | Cai | 2018 | rs2228145 | USA | Atherosclerosis | case-control study | Caucasian | A | C | 5325 | 327474 | 0.9 (0.87, 0.94) |
| 30090940 | Cai | 2018 | rs2228145 | USA | Peripheral vascular disease | case-control study | Other | A | C | 21632 | 311167 | 0.95 (0.93, 0.97) |
| 30090940 | Cai | 2018 | rs2228145 | USA | Atherosclerosis | case-control study | Other | A | C | 7654 | 325145 | 0.92 (0.89, 0.96) |
| 30090940 | Cai | 2018 | rs2228145 | USA | Atherosclerosis | case-control study | Asian | A | C | 10317 | 322482 | 0.93 (0.91, 0.96) |
| 30090940 | Cai | 2018 | rs2228145 | USA | Peripheral vascular disease | case-control study | Other | A | C | 22963 | 309836 | 0.96 (0.94, 0.98) |
| 30090940 | Cai | 2018 | rs2228145 | USA | Skin diseases | case-control study | Other | A | C | 64563 | 268236 | 1.03 (1.01, 1.04) |
| 30090940 | Cai | 2018 | rs2228145 | USA | Skin diseases (dermatitis) | case-control study | Caucasian | A | C | 14976 | 317823 | 1.05 (1.02, 1.07) |
| 30090940 | Cai | 2018 | rs2228145 | USA | Skin diseases (Atopic dermatitis) | case-control study | Caucasian | A | C | 39603 | 293196 | 1.03 (1.01, 1.05) |
| 30090940 | Cai | 2018 | rs2228145 | USA | Skin diseases (dermatosis) | case-control study | Other | A | C | 15309 | 317490 | 1.04 (1.02, 1.07) |
| 30090940 | Cai | 2018 | rs2228145 | USA | Acquired deformities of finger | cohort study | Caucasian | A | C | 998 | 331801 | 1.19 (1.09, 1.31) |
| 30090940 | Cai | 2018 | rs2228145 | USA | Gouty arthropathy | cohort study | Caucasian | A | C | 4992 | 327807 | 1.08 (1.03, 1.13) |
| 30090940 | Cai | 2018 | rs2228145 | USA | Pleurisy/pleural effusion | case-control study | Caucasian | A | C | 5990 | 326809 | 1.07 (1.03, 1.12) |
| 30090940 | Cai | 2018 | rs2228145 | USA | renal disorder | case-control study | Other | A | C | 3661 | 329138 | 1.1 (1.04, 1.16) |
| 30090940 | Cai | 2018 | rs2228145 | USA | Conjunctivitis | cohort study | Mixed | A | C | 4659 | 328140 | 1.08 (1.03, 1.13) |
| 29797122 | Topchieva | 2018 | rs2228145 | Russian | Nonalcoholic Steatohepatitis | cohort study | Mixed | A | C | 115 | 124 | 1.058 (0.726, 1.541) |
| 29775600 | Parisinos | 2018 | rs2228145 | UK | Crohn’s disease | cohort study | Mixed | A | C | 20,550 | 41,642 | 0.948 (0.925, 0.972) |
| 29775600 | Parisinos | 2018 | rs2228145 | UK | ulcerative colitis | cohort study | Mixed | A | C | 17,647 | 47,179 | 0.973 (0.948, 0.998) |
| 29197507 | Khandaker | 2018 | rs2228145 | England | Depression | cohort study | Mixed | A | C | 38 | 3398 | 0.65 (0.4, 1.056) |
| 29197507 | Khandaker | 2018 | rs2228145 | England | Psychosis | cohort study | Mixed | A | C | 54 | 3521 | 0.792 (0.533, 1.176) |
| 29094161 | Hartwig | 2017 | rs222814 | Mix | schizophrenia | case-control study | Caucasian | A | C | 34241 | 45604 | 1.06 (1.01, 1.12) |
| 29042807 | Mitrokhin | 2017 | rs2228044 | Russian | coronary artery disease | case-control study | Caucasian | G | C |  |  | 0.9 (0.53, 1.52) |
| 29042807 | Mitrokhin | 2017 | rs2228043 | Russian | coronary artery disease | case-control study | Caucasian | G | C |  |  | 1.14 (0.69, 1.9) |
| 28974776 | Chan | 2017 | rs7553796 | Germany | schizophrenia | case-control study | Caucasian | C | A | 149 | 198 | 1.537 (1.344, 1.758) |
| 28918391 | Terrell | 2018 | rs2228145 | USA | concussion | cohort study | Mixed | A | C | 133 | 923 | 1.943 (1.3, 2.904) |
| 28879718 | Ahmed | 2017 | rs4845617 | Pakistani | Rheumatoid Arthritis | case-control study | Other | G | A |  |  | 0.438 (0.26, 0.737) |
| 28879718 | Ahmed | 2017 | rs4537545 | Pakistani | Rheumatoid Arthritis | case-control study | Other | C | T | 60 | 60 | 0.716 (0.431, 1.19) |
| 28879718 | Ahmed | 2017 | rs2228145 | Pakistani | Rheumatoid Arthritis | cohort study | Mixed | A | C | 60 | 60 | 0.153 (0.085, 0.274) |
| 28593468 | Ponce | 2018 | rs4845617 | Mexican | hip fracture | case-control study | Other | G | A | 134 | 134 | 0.887 (0.632, 1.245) |
| 28593468 | Ponce | 2018 | rs2228145 | Mexican | hip fracture | cohort study | Mixed | A | C | 134 | 134 | 0.81 (0.576, 1.138) |
| 28543912 | Izakovicova | 2017 | rs2228145 | Czech | Recurrent aphthous stomatitis | cohort study | Mixed | A | C | 64 | 184 | 0.94 (0.62, 1.43) |
| 28442395 | Zhang | 2017 | rs2228145 | China | gastric cancer | cohort study | Mixed | A | C | 473 | 474 | 0.885 (0.738, 1.062) |
| 28334838 | Farahi | 2017 | rs2228145 | Africa America | COPD | cohort study | Mixed | A | C | 821 | 1749 | 1.05 (0.87, 1.27) |
| 28334838 | Farahi | 2017 | rs2228145 | Non-Hispanic White | COPD | cohort study | Mixed | A | C | 2812 | 2534 | 1.02 (0.93, 1.11) |
| 28334838 | Farahi | 2017 | rs2228145 | ECLIPSE | COPD | cohort study | Mixed | A | C | 1764 | 178 | 1.12 (0.88, 1.42) |
| 28334838 | Farahi | 2017 | rs2228145 | NETT/NAS | COPD | cohort study | Mixed | A | C | 373 | 435 | 0.9 (0.73, 1.12) |
| 28334838 | Farahi | 2017 | rs2228145 | Gen/KOLS | COPD | cohort study | Mixed | A | C | 863 | 808 | 1.02 (0.86, 1.2) |
| 28334838 | Farahi | 2017 | rs2228145 | UK Biobank | COPD | cohort study | Mixed | A | C | 886 | 29949 | 1.02 (0.92, 1.12) |
| 28334838 | Farahi | 2017 | rs2228145 | UK Biobank | asthma | cohort study | Mixed | A | C | 13157 | 63859 | 1.02 (0.99, 1.05) |
| 28334838 | Farahi | 2017 | rs2228145 | UK Biobank | Atopic asthma | cohort study | Mixed | A | C | 4405 | 6396 | 1.07 (1.01, 1.13) |
| 28334838 | Farahi | 2017 | rs2228145 | UK Biobank | allergy-related conditions | cohort study | Mixed | A | C | 19850 | 42807 | 1.08 (1.04, 1.11) |
| 28106546 | Haddick | 2017 | rs2228145 | USA | earlier age of Alzheimer’s Disease | case-control study | Caucasian | A | C | 100 | 102 | 1.7 (1.15, 2.55) |
| 28106546 | Haddick | 2017 | rs2228145 | USA | earlier age of Alzheimer’s Disease | case-control study | Caucasian | A | C | 58 | 275 | 1.4 (0.95, 2.18) |
| 28106546 | Haddick | 2017 | rs2228145 | USA | earlier age of Alzheimer’s Disease | case-control study | Caucasian | A | C | 207 | 179 | 1.6 (1.23, 2.14) |
| 28106546 | Haddick | 2017 | rs2228145 | USA | earlier age of Alzheimer’s Disease | case-control study | Caucasian | A | C | 702 | 258 | 1 (0.84, 1.28) |
| 28106546 | Haddick | 2017 | rs2228145 | USA | earlier age of Alzheimer’s Disease | case-control study | Caucasian | A | C | 75 | 75 | 1.2 (0.75, 1.78) |
| 27899403 | Jones | 2017 | rs4129267 | Mix | abdominal aortic aneurysms | GWAS | Caucasian | C | T | 5232 | 7908 | 1.106 (1.049, 1.166) |
| 27899403 | Jones | 2017 | rs4129267 | Mix | abdominal aortic aneurysms | GWAS | Caucasian | C | T | 608 | 612 | 1.189 (1.011, 1.399) |
| 27899403 | Jones | 2017 | rs4129267 | Mix | abdominal aortic aneurysms | GWAS | Caucasian | C | T | 397 | 384 | 1.193 (0.974, 1.464) |
| 27899403 | Jones | 2017 | rs4129267 | Mix | abdominal aortic aneurysms | GWAS | Caucasian | C | T | 1846 | 5605 | 1.139 (1.055, 1.23) |
| 27899403 | Jones | 2017 | rs4129267 | Mix | abdominal aortic aneurysms | GWAS | Caucasian | C | T | 840 | 2791 | 1.222 (1.082, 1.383) |
| 27899403 | Jones | 2017 | rs4129267 | Mix | abdominal aortic aneurysms | GWAS | Caucasian | C | T | 724 | 1231 | 1.224 (1.079, 1.389) |
| 27899403 | Jones | 2017 | rs4129267 | Mix | abdominal aortic aneurysms | GWAS | Caucasian | C | T | 557 | 89235 | 1.138 (1.003, 1.29) |
| 27744395 | Jansen | 2017 | rs2228145 | CARDIoGRAM | Rheumatoid Arthritis | cohort study | Caucasian | A | C | 6579 | 23770 | 0.901 (0.869, 0.934) |
| 27744395 | Jansen | 2017 | rs2228145 | CARDIoGRAM | Coronary Artery Disease | cohort study | Caucasian | A | C | 22233 | 64762 | 0.957 (0.93, 0.984) |
| 27078193 | Pérez-Rubio | 2016 | rs4845626 | Mexican | COPD Severity | case-control study | Other | G | T |  |  | 0.3 (0.12, 0.74) |
| 27078193 | Pérez-Rubio | 2016 | rs4329505 | Mexican | COPD Severity | case-control study | Other | T | C | 299 | 531 | 0.42 (0.23, 0.76) |
| 26997259 | Wang | 2016 | rs2228145 | China | asthma | cohort study | Mixed | A | C | 394 | 395 | 1.099 (0.892, 1.354) |
| 26997259 | Wang | 2016 | rs12083537 | China | asthma | case-control study | Asian | T | C |  |  | 1.129 (0.783, 1.629) |
| 26955245 | Kim | 2016 | rs4845617 | Korean | Ischemic Stroke | case-control study | Asian | G | A | 121 | 291 | 0.832 (0.628, 1.104) |
| 26955245 | Kim | 2016 | rs2228144 | Korean | Ischemic Stroke | case-control study | Asian | G | A |  |  | 0.8 (0.49, 1.29) |
| 26939566 | Ruiz-Larrañaga | 2016 | rs2228145 | Italy | rheumatoid arthritis | case-control study | Other | A | C | 142 | 139 | 0.4 (0.19, 0.87) |
| 26782593 | Song | 2015 | rs4537545 | China | ischemic stroke | case-control study | Asian | C | T |  |  | 0.945 (0.74, 1.207) |
| 26782593 | Song | 2015 | rs4075015 | China | ischemic stroke | case-control study | Asian | A | T | 307 | 227 | 1.086 (0.85, 1.389) |
| 26725994 | Miwa | 2016 | rs2228145 | Japan | dementia | case-control study | Other | A | C | 60 | 803 | 0.87 (0.46, 1.7) |
| 26714766 | Sarsu | 2015 | rs7529229 | Turkey | acute appendicitis | case-control study | Caucasian | T | C | 75 | 75 | 3.622 (0.74, 17.731) |
| 26632999 | Senhaji | 2016 | rs2228145 | Moroccan | inflammatory bowel disease | case-control study | Other | A | C | 199 | 311 | 1.01 (0.769, 1.326) |
| 26632999 | Senhaji | 2016 | rs2228145 | Moroccan | Crohn’s disease | case-control study | Asian | A | C | 136 | 311 | 0.948 (0.694, 1.293) |
| 26632999 | Senhaji | 2016 | rs2228145 | Moroccan | ulcerative colitis | case-control study | Caucasian | A | C | 63 | 311 | 1.153 (0.767, 1.734) |
| 26481614 | Kapelski | 2015 | rs4845617 | Polish | schizophrenia | case-control study | Caucasian | G | A |  |  | 1.043 (0.819, 1.327) |
| 26481614 | Kapelski | 2015 | rs4537545 | Polish | schizophrenia | case-control study | Caucasian | C | T |  |  | 1.241 (0.974, 1.581) |
| 26481614 | Kapelski | 2015 | rs2228145 | Polish | schizophrenia | case-control study | Caucasian | A | C |  |  | 1.288 (1.011, 1.641) |
| 26238946 | Abe | 2015 | rs4845625 | Japan | Hypertriglyceridemia | case-control study | Asian | C | T | 1612 | 3005 | 0.763 (0.641, 0.909) |
| 25813875 | Burger | 2015 | rs2228145 | South Africa | carpal tunnel syndrome | case-control study | Caucasian | A | C |  |  | 2.072 (1.209, 3.552) |
| 25524550 | Horibe | 2015 | rs4845625 | Japan | chronic kidney disease | case-control study | Asian | C | T | 1588 | 659 | 0.699 (0.521, 0.935) |
| 25409741 | Huusko | 2014 | rs4845617 | Finland | bronchopulmonary dysplasia | case-control study | Caucasian | G | A | 56 | 197 | 0.904 (0.589, 1.385) |
| 25409741 | Huusko | 2014 | rs4845617 | Finland | bronchopulmonary dysplasia | case-control study | Caucasian | G | A | 58 | 68 | 1.353 (0.846, 2.166) |
| 25409741 | Huusko | 2014 | rs4845374 | Finland | bronchopulmonary dysplasia | case-control study | Caucasian | T | A | 56 | 197 | 0.947 (0.493, 1.817) |
| 25409741 | Huusko | 2014 | rs4845374 | Finland | bronchopulmonary dysplasia | case-control study | Caucasian | T | A | 56 | 197 | 1.135 (0.496, 2.598) |
| 25409741 | Huusko | 2014 | rs4601580 | Finland | bronchopulmonary dysplasia | case-control study | Caucasian | T | A | 56 | 197 | 0.851 (0.557, 1.301) |
| 25409741 | Huusko | 2014 | rs4601580 | Finland | bronchopulmonary dysplasia | case-control study | Caucasian | T | A | 56 | 197 | 0.965 (0.585, 1.591) |
| 25409741 | Huusko | 2014 | rs4553185 | Finland | bronchopulmonary dysplasia | case-control study | Caucasian | T | C | 56 | 197 | 0.753 (0.488, 1.161) |
| 25409741 | Huusko | 2014 | rs4553185 | Finland | bronchopulmonary dysplasia | case-control study | Caucasian | T | C | 56 | 197 | 1.041 (0.631, 1.718) |
| 25409741 | Huusko | 2014 | rs4453032 | Finland | bronchopulmonary dysplasia | case-control study | Caucasian | A | G | 56 | 197 | 0.814 (0.511, 1.296) |
| 25409741 | Huusko | 2014 | rs4453032 | Finland | bronchopulmonary dysplasia | case-control study | Caucasian | A | G | 56 | 197 | 0.922 (0.557, 1.527) |
| 25409741 | Huusko | 2014 | rs4240872 | Finland | bronchopulmonary dysplasia | case-control study | Caucasian | T | C | 56 | 197 | 1.03 (0.67, 1.584) |
| 25409741 | Huusko | 2014 | rs4240872 | Finland | bronchopulmonary dysplasia | case-control study | Caucasian | T | C | 56 | 197 | 1.205 (0.689, 2.109) |
| 25409741 | Huusko | 2014 | rs4075015 | Finland | bronchopulmonary dysplasia | case-control study | Caucasian | A | T | 56 | 197 | 1.096 (0.718, 1.673) |
| 25409741 | Huusko | 2014 | rs4075015 | Finland | bronchopulmonary dysplasia | case-control study | Caucasian | A | T | 56 | 197 | 1.201 (0.728, 1.982) |
| 25409741 | Huusko | 2014 | rs4072391 | Finland | bronchopulmonary dysplasia | case-control study | Caucasian | C | T | 56 | 197 | 0.911 (0.586, 1.418) |
| 25409741 | Huusko | 2014 | rs4072391 | Finland | bronchopulmonary dysplasia | case-control study | Caucasian | C | T | 56 | 197 | 1.211 (0.647, 2.265) |
| 25409741 | Huusko | 2014 | rs1386821 | Finland | bronchopulmonary dysplasia | case-control study | Caucasian | A | C | 56 | 197 | 1.07 (0.64, 1.789) |
| 25409741 | Huusko | 2014 | rs1386821 | Finland | bronchopulmonary dysplasia | case-control study | Caucasian | A | C | 56 | 197 | 0.754 (0.397, 1.432) |
| 25041016 | Prayong | 2014 | rs2228145 | Thailand | cholangiocarcinoma | case-control study | Other | A | C | 79 | 80 | 0.35 (0.19, 0.63) |
| 25016825 | Liu | 2014 | rs4845626 | China | rheumatoid arthritis | case-control study | Asian | G | T | 162 | 188 | 1.682 (0.963, 2.938) |
| 25016825 | Liu | 2014 | rs11265618 | China | rheumatoid arthritis | case-control study | Asian | C | T | 162 | 188 | 1.949 (1.118, 3.396) |
| 24971461 | Bank | 2014 | rs4537545 | Danish | Crohn’s disease | case-control study | Caucasian | C | T | 624 | 795 | 1.081 (0.928, 1.26) |
| 24971461 | Bank | 2014 | rs4537545 | Danish | ulcerative colitis | case-control study | Caucasian | C | T | 411 | 795 | 1.067 (0.898, 1.269) |
| 24971337 | Zhou | 2014 | rs7529229 | China | coronary heart disease | case-control study | Asian | T | C | 263 | 196 | 1.11 (0.853, 1.445) |
| 24772425 | Shen | 2014 | rs7411976 | China | Tuberculosis | case-control study | Asian | A | C | 353 | 400 | 1.27 (0.79, 2.04) |
| 24772425 | Shen | 2014 | rs4845626 | China | Tuberculosis | case-control study | Asian | G | T | 353 | 400 | 0.9 (0.63, 1.3) |
| 24772425 | Shen | 2014 | rs4845618 | China | Tuberculosis | case-control study | Asian | T | G | 353 | 400 | 1.09 (0.89, 1.34) |
| 24772425 | Shen | 2014 | rs4845617 | China | Tuberculosis | case-control study | Asian | G | A | 353 | 400 | 0.95 (0.77, 1.77) |
| 24772425 | Shen | 2014 | rs3887104 | China | Tuberculosis | case-control study | Asian | C | T | 353 | 400 | 1.33 (0.92, 1.93) |
| 24772425 | Shen | 2014 | rs3828078 | China | Tuberculosis | case-control study | Asian | G | A | 353 | 400 | 1.46 (0.92, 2.31) |
| 24772425 | Shen | 2014 | rs2229238 | China | Tuberculosis | case-control study | Asian | C | T | 353 | 400 | 0.57 (0.39, 0.83) |
| 24772425 | Shen | 2014 | rs2228145 | China | Tuberculosis | case-control study | Caucasian | A | C | 353 | 400 | 0.82 (0.67, 1.01) |
| 24762198 | Yu | 2014 | rs7553796 | China | Colorectal cancer | case-control study | Asian | C | A | 299 | 296 | 0.917 (0.73, 1.151) |
| 24762198 | Yu | 2014 | rs7514452 | China | Colorectal cancer | case-control study | Asian | T | C | 299 | 296 | 0.821 (0.553, 1.219) |
| 24703484 | Emsley | 2014 | rs10752641 | UK | seizures | case-control study | Caucasian | C | G | 98 | 123 | 0.64 (0.39, 1.03) |
| 24703484 | Emsley | 2014 | rs10752641 | UK | seizures | case-control study | Caucasian | C | G | 123 | 181 | 0.94 (0.65, 1.37) |
| 24699044 | He | 2014 | rs7529229 | China | coronary artery disease | case-control study | Asian | T | C | 402 | 804 | 1.219 (1.024, 1.452) |
| 24671014 | Hong | 2014 | rs2228145 | China | Hypertension | cross-sectional | Asian | A | C | 454 | 101 | 0.63 (0.36, 1.12) |
| 24671014 | Hong | 2014 | rs2228145 | China | Cardiovascular disease | cross-sectional | Asian | A | C | 111 | 101 | 0.32 (0.13, 0.76) |
| 24670917 | Slattery | 2014 | rs7549250 | non-Hispanic white | breast cancer | case-control study | Caucasian | T | C | 4157 | 3566 | 0.88 (0.79, 0.97) |
| 24670917 | Slattery | 2014 | rs4509570 | non-Hispanic white | breast cancer | case-control study | Caucasian | C | G | 1847 | 1739 | 1.15 (1, 1.32) |
| 24670917 | Slattery | 2014 | rs4075015 | non-Hispanic white | breast cancer | case-control study | Caucasian | A | T | 4157 | 3567 | 1.136 (1.031, 1.25) |
| 24670917 | Slattery | 2014 | rs2229238 | non-Hispanic white | breast cancer | case-control study | Caucasian | C | T | 1847 | 1742 | 0.81 (0.7, 0.93) |
| 24670917 | Slattery | 2014 | rs1386821 | Hispanic/Native American | breast cancer | case-control study | Other | A | C | 4157 | 3567 | 0.88 (0.79, 0.97) |
| 24670917 | Slattery | 2014 | rs11265618 | non-Hispanic white | breast cancer | case-control study | Caucasian | C | T | 2311 | 1989 | 1.18 (1.01, 1.37) |
| 24668548 | Deng | 2014 | rs6684439 | China | hepatocellular carcinoma | case-control study | Asian | C | T | 192 | 192 | 0.657 (0.476, 0.907) |
| 24668548 | Deng | 2014 | rs2228145 | China | hepatocellular carcinoma | case-control study | Caucasian | A | C | 192 | 192 | 0.723 (0.522, 1.002) |
| 24498998 | Lopez-Mejias | 2013 | rs2228145 | Spanish | Henoch–Schonlein purpura | case-control study | Caucasian | A | C | 285 | 877 | 1.05 (0.86, 1.27) |
| 24423322 | Bhatt | 2014 | rs1386821 | European ancestry | Polycystic Ovary Snydrome | case-control study | Caucasian | A | C | 905 | 955 | 1.36 (1.12, 1.66) |
| 24239840 | Lin | 2014 | rs2228145 | USA | spontaneous miscarriage | case-control study | Caucasian | A | C | 157 | 158 | 0.97 (0.703, 1.339) |
| 24239840 | Lin | 2014 | rs11265611 | USA | atrial fibrillation | case-control study | Caucasian | A | G | 948 | 3330 | 0.7 (0.58, 0.85) |
| 23594084 | Jeon | 2013 | rs4845617 | Korean | systemic lupus erythematosus | case-control study | Asian | G | A | 300 | 299 | 1.024 (0.794, 1.321) |
| 23594084 | Jeon | 2013 | rs4845374 | Korean | systemic lupus erythematosus | case-control study | Asian | T | A | 300 | 299 | 1.029 (0.675, 1.569) |
| 23594084 | Jeon | 2013 | rs2228145 | Korean | systemic lupus erythematosus | case-control study | Caucasian | A | C | 300 | 299 | 0.951 (0.735, 1.229) |
| 23593036 | Ferreira | 2013 | rs2228145 | Mix | type 1 diabetes | case-control study | Caucasian | A | C | 8371 | 18463 | 0.94 (0.91, 0.99) |
| 23593036 | Ferreira | 2013 | rs2228145 | Mix | type 1 diabetes | case-control study | Caucasian | A | C | 5797 | 3771 | 0.92 (0.87, 0.97) |
| 23593036 | Ferreira | 2013 | rs2228145 | Mix | coronary heart disease | case-control study | Caucasian | A | C | 51441 | 187667 | 0.97 (0.95, 0.98) |
| 23593036 | Ferreira | 2013 | rs2228145 | Mix | Atrial Fibrillation | case-control study | Caucasian | A | C | 2260 | 18524 | 0.9 (0.85, 0.95) |
| 23593036 | Ferreira | 2013 | rs2228145 | Mix | abdominal aortic aneurysms | case-control study | Caucasian | A | C | 4524 | 20234 | 0.84 (0.8, 0.89) |
| 23593036 | Ferreira | 2013 | rs2228145 | Mix | Rheumatoid Arthritis | case-control study | Caucasian | A | C | 11475 | 27345 | 0.9 (0.86, 0.93) |
| 23593036 | Ferreira | 2013 | rs2228145 | Mix | Asthma | case-control study | Caucasian | A | C | 15797 | 57800 | 1.09 (1.03, 1.15) |
| 23593036 | Ferreira | 2013 | rs2228145 | Mix | all cancer | case-control study | Asian | A | C | 5376 | 62499 | 0.99 (0.95, 1.04) |
| 23593036 | Ferreira | 2013 | rs2228145 | Mix | breast cancer | GWAS | Caucasian | A | C | 14456 | 30214 | 1.03 (0.96, 1.1) |
| 23593036 | Ferreira | 2013 | rs2228145 | Mix | colorectal cancer | cross-sectional | Asian | A | C | 1863 | 3766 | 1.03 (0.96, 1.12) |
| 23582566 | Esparza-Gordillo | 2013 | rs2228145 | Mix | Skin diseases (Atopic dermatitis) | case-control study | Caucasian | A | C | 7130 | 9253 | 1.15 (1.09, 1.21) |
| 23582566 | Esparza-Gordillo | 2013 | rs2228145 | Mix | Transient course | case-control study | Caucasian | A | C | 639 | 1729 | 1.03 (0.91, 1.18) |
| 23582566 | Esparza-Gordillo | 2013 | rs2228145 | Mix | Transient course | case-control study | Caucasian | A | C | 135 | 366 | 1.07 (0.8, 1.43) |
| 23582566 | Esparza-Gordillo | 2013 | rs2228145 | Mix | Persistent course | case-control study | Caucasian | A | C | 714 | 1729 | 1.19 (1.06, 1.35) |
| 23582566 | Esparza-Gordillo | 2013 | rs2228145 | Mix | Persistent course | case-control study | Caucasian | A | C | 99 | 366 | 1.37 (1, 1.87) |
| 23582566 | Esparza-Gordillo | 2013 | rs2228145 | Mix | asthma | case-control study | Caucasian | A | C | 647 | 4894 | 1.04 (0.92, 1.17) |
| 23372742 | Stone | 2013 | rs4537545 | USA | Castleman Disease | case-control study | Caucasian | C | T | 58 | 50 | 1.961 (1.128, 3.411) |
| 23372742 | Stone | 2013 | rs2228145 | USA | Castleman Disease | case-control study | Caucasian | A | C | 58 | 50 | 1.706 (0.97, 3.001) |
| 23202125 | CARDIoGRAMplusC4D | 2013 | rs4845625 | European or south Asian | coronary artery disease | GWAS | Mixed | C | T | 18014 | 40925 | 1.25 (1.01, 1.48) |
| 23202125 | CARDIoGRAMplusC4D | 2013 | rs4845625 | European or south Asian | coronary artery disease | GWAS | Mixed | C | T |  |  | 1.02 (0.94, 1.11) |
| 23202125 | CARDIoGRAMplusC4D | 2013 | rs4845625 | European or south Asian | coronary artery disease | GWAS | Mixed | C | T |  |  | 1.05 (1, 1.09) |
| 23202125 | CARDIoGRAMplusC4D | 2013 | rs4845625 | European or south Asian | coronary artery disease | GWAS | Mixed | C | T |  |  | 1.21 (1.06, 1.36) |
| 23202125 | CARDIoGRAMplusC4D | 2013 | rs4845625 | European or south Asian | coronary artery disease | GWAS | Mixed | C | T |  |  | 1.12 (1, 1.23) |
| 23202125 | CARDIoGRAMplusC4D | 2013 | rs4845625 | European or south Asian | coronary artery disease | GWAS | Mixed | C | T |  |  | 1.02 (0.91, 1.14) |
| 23202125 | CARDIoGRAMplusC4D | 2013 | rs4845625 | European or south Asian | coronary artery disease | GWAS | Mixed | C | T |  |  | 1.04 (0.96, 1.13) |
| 23202125 | CARDIoGRAMplusC4D | 2013 | rs4845625 | European or south Asian | coronary artery disease | GWAS | Mixed | C | T |  |  | 0.96 (0.8, 1.13) |
| 23202125 | CARDIoGRAMplusC4D | 2013 | rs4845625 | European or south Asian | coronary artery disease | GWAS | Mixed | C | T |  |  | 0.98 (0.87, 1.08) |
| 23202125 | CARDIoGRAMplusC4D | 2013 | rs4845625 | European or south Asian | coronary artery disease | GWAS | Mixed | C | T |  |  | 1.16 (1.05, 1.27) |
| 23202125 | CARDIoGRAMplusC4D | 2013 | rs4845625 | European or south Asian | coronary artery disease | GWAS | Mixed | C | T |  |  | 1.09 (0.91, 1.26) |
| 23202125 | CARDIoGRAMplusC4D | 2013 | rs4845625 | European or south Asian | coronary artery disease | GWAS | Mixed | C | T |  |  | 1.11 (1.02, 1.21) |
| 23202125 | CARDIoGRAMplusC4D | 2013 | rs4845625 | European or south Asian | coronary artery disease | GWAS | Mixed | C | T | 40365 | 63714 | 1.15 (1, 1.3) |
| 23202125 | CARDIoGRAMplusC4D | 2013 | rs4845625 | European or south Asian | coronary artery disease | GWAS | Mixed | C | T |  |  | 1 (0.83, 1.18) |
| 23202125 | CARDIoGRAMplusC4D | 2013 | rs4845625 | European or south Asian | coronary artery disease | GWAS | Mixed | C | T |  |  | 1.11 (0.97, 1.25) |
| 23202125 | CARDIoGRAMplusC4D | 2013 | rs4845625 | European or south Asian | coronary artery disease | GWAS | Mixed | C | T |  |  | 0.99 (0.67, 1.31) |
| 23202125 | CARDIoGRAMplusC4D | 2013 | rs4845625 | European or south Asian | coronary artery disease | GWAS | Mixed | C | T |  |  | 1.22 (0.97, 1.47) |
| 23202125 | CARDIoGRAMplusC4D | 2013 | rs4845625 | European or south Asian | coronary artery disease | GWAS | Mixed | C | T |  |  | 0.98 (0.83, 1.14) |
| 23202125 | CARDIoGRAMplusC4D | 2013 | rs4845625 | European or south Asian | coronary artery disease | GWAS | Mixed | C | T |  |  | 1.03 (0.83, 1.23) |
| 23202125 | CARDIoGRAMplusC4D | 2013 | rs4845625 | European or south Asian | coronary artery disease | GWAS | Mixed | C | T |  |  | 1 (0.88, 1.12) |
| 23202125 | CARDIoGRAMplusC4D | 2013 | rs4845625 | European or south Asian | coronary artery disease | GWAS | Mixed | C | T |  |  | 1.1 (0.99, 1.22) |
| 23202125 | CARDIoGRAMplusC4D | 2013 | rs4845625 | European or south Asian | coronary artery disease | GWAS | Mixed | C | T |  |  | 0.98 (0.82, 1.14) |
| 23202125 | CARDIoGRAMplusC4D | 2013 | rs4845625 | European or south Asian | coronary artery disease | GWAS | Mixed | C | T |  |  | 1.07 (0.96, 1.18) |
| 23202125 | CARDIoGRAMplusC4D | 2013 | rs4845625 | European or south Asian | coronary artery disease | GWAS | Mixed | C | T |  |  | 0.98 (0.85, 1.11) |
| 23202125 | CARDIoGRAMplusC4D | 2013 | rs4845625 | European or south Asian | coronary artery disease | GWAS | Mixed | C | T |  |  | 1.14 (1.05, 1.24) |
| 23202125 | CARDIoGRAMplusC4D | 2013 | rs4845625 | European or south Asian | coronary artery disease | GWAS | Mixed | C | T |  |  | 1.07 (0.88, 1.27) |
| 23202125 | CARDIoGRAMplusC4D | 2013 | rs4845625 | European or south Asian | coronary artery disease | GWAS | Mixed | C | T |  |  | 1.05 (0.98, 1.12) |
| 23202125 | CARDIoGRAMplusC4 | 2013 | rs4845625 | European or south Asian | coronary artery disease | GWAS | Mixed | C | T |  |  | 0.97 (0.87, 1.08) |
| 23202125 | CARDIoGRAMplusC4D | 2013 | rs4845625 | European or south Asian | coronary artery disease | GWAS | Mixed | C | T |  |  | 1.03 (0.83, 1.23) |
| 23202125 | CARDIoGRAMplusC4D | 2013 | rs4845625 | European or south Asian | coronary artery disease | GWAS | Mixed | C | T |  |  | 0.94 (0.83, 1.05) |
| 23202125 | CARDIoGRAMplusC4D | 2013 | rs4845625 | European or south Asian | coronary artery disease | GWAS | Mixed | C | T |  |  | 0.92 (0.61, 1.22) |
| 23202125 | CARDIoGRAMplusC4D | 2013 | rs4845625 | European or south Asian | coronary artery disease | GWAS | Mixed | C | T |  |  | 0.96 (0.68, 1.24) |
| 23202125 | CARDIoGRAMplusC4D | 2013 | rs4845625 | European or south Asian | coronary artery disease | GWAS | Mixed | C | T |  |  | 1.11 (0.79, 1.43) |
| 23202125 | CARDIoGRAMplusC4D | 2013 | rs4845625 | European or south Asian | coronary artery disease | GWAS | Mixed | C | T |  |  | 0.86 (0.56, 1.16) |
| 23202125 | CARDIoGRAMplusC4D | 2013 | rs4845625 | European or south Asian | coronary artery disease | GWAS | Mixed | C | T |  |  | 0.95 (0.84, 1.06) |
| 23202125 | CARDIoGRAMplusC4D | 2013 | rs4845625 | European or south Asian | coronary artery disease | GWAS | Mixed | C | T |  |  | 0.75 (0.28, 1.22) |
| 23202125 | CARDIoGRAMplusC4D | 2013 | rs4845625 | European or south Asian | coronary artery disease | GWAS | Mixed | C | T |  |  | 1.11 (1.01, 1.22) |
| 23202125 | CARDIoGRAMplusC4D | 2013 | rs4845625 | European or south Asian | coronary artery disease | GWAS | Mixed | C | T |  |  | 1.07 (0.93, 1.2) |
| 23202125 | CARDIoGRAMplusC4D | 2013 | rs4845625 | European or south Asian | coronary artery disease | GWAS | Mixed | C | T |  |  | 1.04 (0.97, 1.1) |
| 23202125 | CARDIoGRAMplusC4D | 2013 | rs4845625 | European or south Asian | coronary artery disease | GWAS | Mixed | C | T |  |  | 1.03 (0.96, 1.1) |
| 23202125 | CARDIoGRAMplusC4D | 2013 | rs4845625 | European or south Asian | coronary artery disease | GWAS | Mixed | C | T |  |  | 1.13 (1.04, 1.23) |
| 23202125 | CARDIoGRAMplusC4D | 2013 | rs4845625 | European or south Asian | coronary artery disease | GWAS | Mixed | C | T |  |  | 1.01 (0.92, 1.11) |
| 23202125 | CARDIoGRAMplusC4D | 2013 | rs4845625 | European or south Asian | coronary artery disease | GWAS | Mixed | C | T |  |  | 1.11 (0.95, 1.28) |
| 23202125 | CARDIoGRAMplusC4D | 2013 | rs4845625 | European or south Asian | coronary artery disease | GWAS | Mixed | C | T |  |  | 1 (0.83, 1.16) |
| 23202125 | CARDIoGRAMplusC4D | 2013 | rs4845625 | European or south Asian | coronary artery disease | GWAS | Mixed | C | T |  |  | 0.96 (0.68, 1.24) |
| 23202125 | CARDIoGRAMplusC4D | 2013 | rs4845625 | European or south Asian | coronary artery disease | GWAS | Mixed | C | T |  |  | 1.17 (1.05, 1.28) |
| 23202125 | CARDIoGRAMplusC4D | 2013 | rs4845625 | European or south Asian | coronary artery disease | GWAS | Mixed | C | T | 5055 | 5617 | 1.09 (1.033, 1.15) |
| 23148991 | You | 2013 | rs4845617 | China | rheumatoid arthritis | case-control study | Asian | G | A | 452 | 373 | 0.764 (0.628, 0.928) |
| 23148991 | You | 2013 | rs4845374 | China | rheumatoid arthritis | case-control study | Asian | T | A | 452 | 373 | 0.893 (0.628, 1.269) |
| 23148991 | You | 2013 | rs2228145 | China | rheumatoid arthritis | case-control study | Caucasian | A | C | 452 | 373 | 0.776 (0.636, 0.946) |
| 23143596 | Eyre | 2012 | rs2228145 | Mix | rheumatoid arthritis | case-control study | Caucasian | A | C | 11475 | 27345 | 0.9 (0.86, 0.93) |
| 23111417 | Harrison | 2013 | rs7529229 | Mix | abdominal aortic aneurysms | case-control study | Caucasian | T | C | 62 | 819 | 0.76 (0.57, 1.01) |
| 23111417 | Harrison | 2013 | rs7529229 | Mix | abdominal aortic aneurysms | case-control study | Caucasian | T | C | 631 | 6342 | 0.79 (0.7, 0.9) |
| 23111417 | Harrison | 2013 | rs7529229 | Mix | abdominal aortic aneurysms | case-control study | Caucasian | T | C | 867 | 2000 | 0.83 (0.73, 0.94) |
| 23111417 | Harrison | 2013 | rs7529229 | Mix | abdominal aortic aneurysms | case-control study | Caucasian | T | C | 1373 | 718 | 0.84 (0.75, 0.94) |
| 23111417 | Harrison | 2013 | rs7529229 | Mix | abdominal aortic aneurysms | case-control study | Caucasian | T | C | 1596 | 5855 | 0.87 (0.81, 0.94) |
| 23094986 | Hsieh | 2012 | rs2228145 | Taiwan,China | obesity | cohort study | Caucasian | A | C | 451 |  | 1.09 (0.74, 1.61) |
| 23094986 | Hsieh | 2012 | rs2228145 | Taiwan,China | obesity | case-control study | Caucasian | A | C | 474 |  | 1.43 (0.98, 2.1) |
| 23094986 | Hsieh | 2012 | rs2228145 | Taiwan,China | metabolic syndrome | case-control study | Caucasian | A | C | 451 |  | 0.63 (0.31, 1.29) |
| 23094986 | Hsieh | 2012 | rs2228145 | Taiwan,China | metabolic syndrome | case-control study | Caucasian | A | C | 474 |  | 2.4 (1.26, 4.55) |
| 23078005 | Rausz | 2013 | rs2228145 | Hungary | Mastocytosis | case-control study | Caucasian | A | C | 66 | 99 | 1.693 (1.074, 2.668) |
| 23073775 | Chen | 2013 | rs7529229 | China | angina pectoris | case-control study | Asian | T | C | 72 | 231 | 1.031 (0.708, 1.502) |
| 23073775 | Chen | 2013 | rs7529229 | China | angina pectoris | case-control study | Asian | T | C | 79 | 231 | 0.923 (0.641, 1.33) |
| 23073775 | Chen | 2013 | rs7529229 | China | coronary heart disease | case-control study | Asian | T | C | 187 | 231 | 1.01 (0.77, 1.33) |
| 23073775 | Chen | 2013 | rs7529229 | China | myocardial infarction | case-control study | Asian | T | C | 36 | 231 | 1.061 (0.644, 1.746) |
| 23073775 | Chen | 2013 | rs2228145 | China | coronary heart disease | case-control study | Asian | A | C | 187 | 231 | 0.97 (0.71, 1.28) |
| 23073775 | Chen | 2013 | rs2228145 | China | angina pectoris | case-control study | Caucasian | A | C | 72 | 231 | 1.102 (0.758, 1.604) |
| 23073775 | Chen | 2013 | rs2228145 | China | angina pectoris | cohort study | Asian | A | C | 79 | 231 | 0.955 (0.664, 1.374) |
| 23073775 | Chen | 2013 | rs2228145 | China | myocardial infarction | case-control study | Caucasian | A | C | 36 | 231 | 1.042 (0.633, 1.716) |
| 22742541 | Cenit | 2012 | rs2228145 | Spanish | Systemic sclerosis | case-control study | Caucasian | A | C | 1013 | 1375 | 0.98 (0.87, 1.1) |
| 22642608 | Perez-Bravo | 2012 | rs2228145 | Chilean | type 1 diabetes | case-control study | Caucasian | A | C | 145 | 103 | 0.613 (0.426, 0.884) |
| 22553514 | Zhou | 2010 | rs2228145 | China | glaucoma | case-control study | Caucasian | A | C | 37 | 100 | 6.373 (4.12, 9.857) |
| 22421340 | Swerdlow | 2012 | rs7529229 | Mix | All cancer | case-control study | Caucasian | T | C | 22504 | 58743 | 1 (0.96, 1.04) |
| 22421340 | Swerdlow | 2012 | rs7529229 | Mix | breast cancer | case-control study | Caucasian | T | C | 14726 | 21484 | 1 (0.95, 1.06) |
| 22421340 | Swerdlow | 2012 | rs7529229 | Mix | cardiovascular disease | case-control study | Caucasian | T | C | 17595 | 76321 | 0.98 (0.95, 1) |
| 22421340 | Swerdlow | 2012 | rs7529229 | Mix | Colorectal cancer | case-control study | Caucasian | T | C | 1863 | 1002 | 1.03 (0.96, 1.12) |
| 22421340 | Swerdlow | 2012 | rs7529229 | Mix | coronary heart disease | case-control study | Caucasian | T | C | 95 | 2173 | 0.59 (0.43, 0.82) |
| 22421340 | Swerdlow | 2012 | rs7529229 | Mix | coronary heart disease | case-control study | Caucasian | T | C | 100 | 2195 | 1.15 (0.86, 1.53) |
| 22421340 | Swerdlow | 2012 | rs7529229 | Mix | coronary heart disease | case-control study | Caucasian | T | C | 115 | 1158 | 0.76 (0.57, 1.01) |
| 22421340 | Swerdlow | 2012 | rs7529229 | Mix | coronary heart disease | case-control study | Caucasian | T | C | 128 | 3632 | 1.01 (0.78, 1.3) |
| 22421340 | Swerdlow | 2012 | rs7529229 | Mix | coronary heart disease | case-control study | Caucasian | T | C | 145 | 908 | 0.85 (0.65, 1.09) |
| 22421340 | Swerdlow | 2012 | rs7529229 | Mix | coronary heart disease | case-control study | Caucasian | T | C | 159 | 1176 | 0.91 (0.72, 1.16) |
| 22421340 | Swerdlow | 2012 | rs7529229 | Mix | coronary heart disease | case-control study | Caucasian | T | C | 269 | 2398 | 0.93 (0.78, 1.11) |
| 22421340 | Swerdlow | 2012 | rs7529229 | Mix | coronary heart disease | case-control study | Caucasian | T | C | 277 | 1127 | 1.06 (0.87, 1.27) |
| 22421340 | Swerdlow | 2012 | rs7529229 | Mix | coronary heart disease | case-control study | Caucasian | T | C | 289 | 3578 | 0.94 (0.79, 1.12) |
| 22421340 | Swerdlow | 2012 | rs7529229 | Mix | coronary heart disease | case-control study | Caucasian | T | C | 299 | 3475 | 0.98 (0.83, 1.16) |
| 22421340 | Swerdlow | 2012 | rs7529229 | Mix | coronary heart disease | case-control study | Caucasian | T | C | 301 | 3164 | 0.85 (0.71, 1.02) |
| 22421340 | Swerdlow | 2012 | rs7529229 | Mix | coronary heart disease | case-control study | Caucasian | T | C | 312 | 578 | 1.15 (0.95, 1.4) |
| 22421340 | Swerdlow | 2012 | rs7529229 | Mix | coronary heart disease | case-control study | Caucasian | T | C | 410 | 441 | 0.88 (0.74, 1.05) |
| 22421340 | Swerdlow | 2012 | rs7529229 | Mix | coronary heart disease | case-control study | Caucasian | T | C | 418 | 4624 | 0.91 (0.79, 1.05) |
| 22421340 | Swerdlow | 2012 | rs7529229 | Mix | coronary heart disease | case-control study | Caucasian | T | C | 435 | 6265 | 1 (0.87, 1.16) |
| 22421340 | Swerdlow | 2012 | rs7529229 | Mix | coronary heart disease | case-control study | Caucasian | T | C | 589 | 4654 | 0.97 (0.85, 1.09) |
| 22421340 | Swerdlow | 2012 | rs7529229 | Mix | coronary heart disease | case-control study | Caucasian | T | C | 617 | 2657 | 1.02 (0.9, 1.16) |
| 22421340 | Swerdlow | 2012 | rs7529229 | Mix | coronary heart disease | case-control study | Caucasian | T | C | 648 | 6437 | 0.95 (0.84, 1.07) |
| 22421340 | Swerdlow | 2012 | rs7529229 | Mix | coronary heart disease | case-control study | Caucasian | T | C | 668 | 3206 | 0.99 (0.88, 1.12) |
| 22421340 | Swerdlow | 2012 | rs7529229 | Mix | coronary heart disease | case-control study | Caucasian | T | C | 676 | 6238 | 1.02 (0.91, 1.15) |
| 22421340 | Swerdlow | 2012 | rs7529229 | Mix | coronary heart disease | case-control study | Caucasian | T | C | 737 | 8021 | 0.98 (0.88, 1.1) |
| 22421340 | Swerdlow | 2012 | rs7529229 | Mix | coronary heart disease | case-control study | Caucasian | T | C | 896 | 5761 | 0.91 (0.81, 1.02) |
| 22421340 | Swerdlow | 2012 | rs7529229 | Mix | coronary heart disease | case-control study | Caucasian | T | C | 1303 | 3880 | 0.96 (0.88, 1.05) |
| 22421340 | Swerdlow | 2012 | rs7529229 | Mix | coronary heart disease | case-control study | Caucasian | T | C | 1837 | 7692 | 0.96 (0.89, 1.03) |
| 22421340 | Swerdlow | 2012 | rs7529229 | Mix | coronary heart disease | case-control study | Caucasian | T | C | 2396 | 2094 | 0.99 (0.91, 1.08) |
| 22421340 | Swerdlow | 2012 | rs7529229 | Mix | coronary heart disease | case-control study | Caucasian | T | C | 51 | 252 | 1.17 (0.76, 1.8) |
| 22421340 | Swerdlow | 2012 | rs7529229 | Mix | coronary heart disease | case-control study | Caucasian | T | C | 60 | 522 | 0.94 (0.64, 1.4) |
| 22421340 | Swerdlow | 2012 | rs7529229 | Mix | coronary heart disease | case-control study | Caucasian | T | C | 509 | 553 | 0.92 (0.78, 1.08) |
| 22421340 | Swerdlow | 2012 | rs7529229 | Mix | coronary heart disease | case-control study | Caucasian | T | C | 632 | 1000 | 1.06 (0.92, 1.22) |
| 22421340 | Swerdlow | 2012 | rs7529229 | Mix | coronary heart disease | case-control study | Caucasian | T | C | 796 | 895 | 1.03 (0.9, 1.18) |
| 22421340 | Swerdlow | 2012 | rs7529229 | Mix | coronary heart disease | case-control study | Caucasian | T | C | 1222 | 1298 | 0.95 (0.87, 1.04) |
| 22421340 | Swerdlow | 2012 | rs7529229 | Mix | coronary heart disease | case-control study | Caucasian | T | C | 1926 | 2937 | 0.89 (0.82, 0.97) |
| 22421340 | Swerdlow | 2012 | rs7529229 | Mix | coronary heart disease | case-control study | Caucasian | T | C | 2073 | 1493 | 0.88 (0.8, 0.97) |
| 22421340 | Swerdlow | 2012 | rs7529229 | Mix | coronary heart disease | case-control study | Caucasian | T | C | 4070 | 4258 | 0.92 (0.85, 0.99) |
| 22421340 | Swerdlow | 2012 | rs7529229 | Mix | stroke | case-control study | Caucasian | T | C | 6904 | 90512 | 0.98 (0.94, 1.02) |
| 22421340 | Swerdlow | 2012 | rs7529229 | Mix | Type 2 Diabetes | case-control study | Caucasian | T | C | 12859 | 86807 | 0.97 (0.94, 1) |
| 22421339 | Sarwar | 2012 | rs2228145 | Mix | Type 2 Diabetes | case-control study | Caucasian | A | C | 9722 | 80095 | 0.99 (0.96, 1.03) |
| 22421339 | Sarwar | 2012 | rs2228145 | Mix | coronary heart disease | case-control study | Caucasian | A | C | 51441 | 136226 | 0.966 (0.95, 0.982) |
| 22228719 | Tabassum | 2012 | rs7514452 | India | Obesity | case-control study | Other | T | C | 453 | 830 | 1.36 (1.12, 1.67) |
| 22228719 | Tabassum | 2012 | rs7514452 | India | Obesity | case-control study | Other | T | C | 444 | 1399 | 1.06 (0.88, 1.27) |
| 22072558 | Stephens | 2012 | rs2228145 | USA | multiple myeloma | case-control study | African | A | C | 626 | 44 | 1.749 (1.086, 2.818) |
| 21981268 | Lopez-Mejias | 2011 | rs2228145 | Spain | cardiovascular disease | case-control study | Other | A | C | 220 | 1030 | 0.89 (0.72, 1.11) |
| 21907864 | Ferreira | 2011 | rs4129267 | Australia | asthma | GWAS | Caucasian | C | T | 2110 | 3857 | 1.09 (1.01, 1.18) |
| 21907864 | Ferreira | 2011 | rs4129267 | Australia | asthma | GWAS | Caucasian | C | T | 10365 | 16110 | 1.09 (1.05, 1.13) |
| 21907864 | Ferreira | 2011 | rs4129267 | Australia | asthma | GWAS | Caucasian | C | T | 1716 | 16888 | 1.08 (1, 1.15) |
| 21907864 | Ferreira | 2011 | rs4129267 | Australia | asthma | GWAS | Caucasian | C | T | 654 | 621 | 1.07 (0.92, 1.25) |
| 21907864 | Ferreira | 2011 | rs4129267 | Australia | asthma | GWAS | Caucasian | C | T | 602 | 2206 | 1.1 (0.97, 1.23) |
| 21907864 | Ferreira | 2011 | rs4129267 | Australia | asthma | GWAS | Caucasian | C | T | 350 | 2321 | 1.16 (1, 1.32) |
| 21851175 | van Munster | 2011 | rs2228145 | Netherlands | delirium | case-control study | Caucasian | A | C | 311 | 559 | 1.068 (0.868, 1.314) |
| 21846873 | Schnabel | 2011 | rs7529229 | European+German | Atrial Fibrillation | GWAS | Caucasian |  | C | 2728 | 16702 | 0.9 (0.85, 0.96) |
| 21846873 | Schnabel | 2011 | rs4845625 | European | Atrial Fibrillation | GWAS | Caucasian | C | T | 2260 | 16264 | 1.111 (1.053, 1.176) |
| 21846873 | Schnabel | 2011 | rs4845625 | African American | Atrial Fibrillation | GWAS | African | C | T | 263 | 3399 | 1.163 (0.971, 1.389) |
| 21846873 | Schnabel | 2011 | rs4845625 | German | Atrial Fibrillation | GWAS | Caucasian | C | T | 468 | 438 | 1.408 (1.124, 1.754) |
| 21846873 | Schnabel | 2011 | rs4537545 | European+German | Atrial Fibrillation | GWAS | Caucasian | C | T | 2728 | 16702 | 0.901 (0.847, 0.952) |
| 21846873 | Schnabel | 2011 | rs28638007 | European+German | Atrial Fibrillation | GWAS | Caucasian | T | C | 2728 | 16702 | 0.9 (0.85, 0.95) |
| 21700295 | Sasayama | 2011 | rs2228145 | Japan | schizophrenia | case-control study | Caucasian | A | C | 104 | 112 | 1.299 (0.886, 1.905) |
| 21523452 | Madeleine | 2011 | rs4509570 | Seattle | breast cancer | case-control study | Caucasian | C | G | 882 | 906 | 1 (0.8, 1.2) |
| 21523452 | Madeleine | 2011 | rs2228145 | Seattle | breast cancer | case-control study | Caucasian | A | C | 882 | 906 | 1.1 (0.9, 1.2) |
| 21523452 | Madeleine | 2011 | rs1386821 | Seattle | breast cancer | case-control study | Caucasian | A | C | 882 | 906 | 1.1 (0.9, 1.3) |
| 20951753 | Rantala | 2011 | rs6427641 | Finland | respiratory tract infections | case-control study | Caucasian | A | G | 264 | 247 | 1.4 (1.07, 1.82) |
| 20951753 | Rantala | 2011 | rs4845617 | Finland | respiratory tract infections | case-control study | Caucasian | G | A | 264 | 247 | 1.48 (1.12, 1.97) |
| 20951753 | Rantala | 2011 | rs4845371 | Finland | respiratory tract infections | case-control study | Caucasian | T | C | 264 | 247 | 1.071 (0.838, 1.369) |
| 20951753 | Rantala | 2011 | rs4601580 | Finland | respiratory tract infections | case-control study | Caucasian | T | A | 264 | 247 | 1.227 (0.959, 1.571) |
| 20951753 | Rantala | 2011 | rs2228145 | Finland | respiratory tract infections | case-control study | Asian | A | C | 264 | 247 | 1.171 (0.897, 1.528) |
| 19567438 | Elliott | 2009 | rs4537545 | Mix | coronary heart disease | case-control study | Caucasian | C | T | 2073 | 1493 | 0.88 (0.8, 0.97) |
| 19567438 | Elliott | 2009 | rs4537545 | Mix | coronary heart disease | case-control study | Caucasian | C | T | 1926 | 2937 | 0.89 (0.82, 0.97) |
| 19567438 | Elliott | 2009 | rs4537545 | Mix | coronary heart disease | case-control study | Caucasian | C | T | 896 | 5761 | 0.91 (0.82, 1.02) |
| 19567438 | Elliott | 2009 | rs4537545 | Mix | coronary heart disease | case-control study | Caucasian | C | T | 4070 | 4258 | 0.92 (0.86, 0.99) |
| 19567438 | Elliott | 2009 | rs4537545 | Mix | coronary heart disease | case-control study | Caucasian | C | T | 1222 | 1298 | 0.95 (0.88, 1.04) |
| 19567438 | Elliott | 2009 | rs4537545 | Mix | coronary heart disease | case-control study | Caucasian | C | T | 1137 | 1215 | 1 (0.88, 1.13) |
| 19567438 | Elliott | 2009 | rs4537545 | Mix | coronary heart disease | case-control study | Caucasian | C | T | 1250 | 12568 | 1.02 (0.93, 1.12) |
| 19567438 | Elliott | 2009 | rs4537545 | Mix | coronary heart disease | case-control study | Caucasian | C | T | 796 | 895 | 1.03 (0.89, 1.18) |
| 19406470 | Aladzsity | 2009 | rs2228145 | Hungary | myelodysplastic syndrome | case-control study | Asian | A | C | 102 | 99 | 1.068 (0.679, 1.679) |
| 19406470 | Aladzsity | 2009 | rs2228145 | Hungary | multiple myeloma | case-control study | Asian | A | C | 100 | 99 | 1.247 (0.818, 1.902) |
| 19280716 | Dema | 2009 | rs2228145 | Spain | celiac disease | case-control study | Asian | A | C | 374 | 853 | 1.24 (1.032, 1.488) |
| 19124510 | Birmann | 2009 | rs7529229 | USA | Multiple Myeloma | case-control study | Caucasian | T | C | 82 | 164 | 0.697 (0.476, 1.02) |
| 19124510 | Birmann | 2009 | rs6684439 | USA | Multiple Myeloma | case-control study | Caucasian | C | T | 82 | 164 | 0.659 (0.45, 0.965) |
| 19124510 | Birmann | 2009 | rs4845623 | USA | Multiple Myeloma | case-control study | Caucasian | A | G | 82 | 164 | 0.739 (0.49, 1.113) |
| 19124510 | Birmann | 2009 | rs4845617 | USA | Multiple Myeloma | case-control study | Caucasian | G | A | 82 | 164 | 1.261 (0.857, 1.856) |
| 19124510 | Birmann | 2009 | rs4845374 | USA | Multiple Myeloma | case-control study | Caucasian | T | A | 82 | 164 | 1.06 (0.64, 1.753) |
| 19124510 | Birmann | 2009 | rs4075015 | USA | Multiple Myeloma | case-control study | Caucasian | A | T | 82 | 164 | 1.02 (0.689, 1.509) |
| 19124510 | Birmann | 2009 | rs2229238 | USA | Multiple Myeloma | case-control study | Caucasian | C | T | 82 | 164 | 2.082 (1.137, 3.813) |
| 19124510 | Birmann | 2009 | rs2228145 | USA | Multiple Myeloma | case-control study | Caucasian | A | C | 82 | 164 | 0.723 (0.491, 1.063) |
| 19124510 | Birmann | 2009 | rs12083537 | USA | Multiple Myeloma | case-control study | Caucasian | T | C | 82 | 164 | 1.252 (0.774, 2.025) |
| 19124510 | Birmann | 2009 | rs10752641 | USA | Multiple Myeloma | case-control study | Caucasian | C | G | 82 | 164 | 1.696 (1.03, 2.792) |
| 19026125 | Storz | 2008 | rs2228145 | German | Behçet’s disease | case-control study | Caucasian | C | A | 94 | 50 | 1.355 (0.827, 2.22) |
| 19026125 | Storz | 2008 | rs2228145 | Turkish | Behçet’s disease | case-control study | Asian | C | A | 30 | 20 | 3.5 (1.643, 7.455) |
| 18853133 | Rafiq | 2008 | rs2228145 | UK,USA,Finland | Type 2 Diabetes | cross-sectional | Asian | A | C | 4549 | 5579 | 0.96 (0.9, 1.02) |
| 18781131 | Gu | 2008 | rs6684439 | USA | melanoma | case-control study | Caucasian | C | T | 219 | 219 | 1.128 (0.858, 1.483) |
| 18781131 | Gu | 2008 | rs4845622 | USA | melanoma | case-control study | Caucasian | A | C | 219 | 219 | 0.906 (0.687, 1.194) |
| 18781131 | Gu | 2008 | rs4845618 | USA | melanoma | case-control study | Caucasian | T | G | 219 | 219 | 0.982 (0.745, 1.293) |
| 18781131 | Gu | 2008 | rs4845617 | USA | melanoma | case-control study | Caucasian | G | A | 219 | 219 | 0.926 (0.701, 1.222) |
| 18781131 | Gu | 2008 | rs4329505 | USA | melanoma | case-control study | Caucasian | T | C | 219 | 219 | 0.925 (0.635, 1.349) |
| 18781131 | Gu | 2008 | rs4240872 | USA | melanoma | case-control study | Caucasian | T | C | 219 | 219 | 0.887 (0.588, 1.336) |
| 18781131 | Gu | 2008 | rs4075015 | USA | melanoma | case-control study | Caucasian | A | T | 219 | 219 | 1.175 (0.893, 1.548) |
| 18781131 | Gu | 2008 | rs2229238 | USA | melanoma | case-control study | Caucasian | C | T | 219 | 219 | 0.832 (0.577, 1.199) |
| 18781131 | Gu | 2008 | rs2228145 | USA | melanoma | case-control study | Caucasian | A | C | 219 | 219 | 0.884 (0.672, 1.163) |
| 18781131 | Gu | 2008 | rs12083537 | USA | melanoma | case-control study | Caucasian | T | C | 219 | 219 | 0.746 (0.538, 1.034) |
| 18276608 | Velez | 2008 | rs952146 | USA | Spontaneous preterm birth maternal | case-control study | Caucasian | A | G | 145 | 198 | 1.035 (0.757, 1.415) |
| 18276608 | Velez | 2008 | rs952146 | USA | Spontaneous preterm birth fetal | case-control study | Caucasian | A | G | 140 | 179 | 1.088 (0.79, 1.499) |
| 18276608 | Velez | 2008 | rs952146 | African-American | Spontaneous preterm birth maternal | case-control study | African | A | G | 76 | 191 | 1.041 (0.71, 1.527) |
| 18276608 | Velez | 2008 | rs952146 | African-American | Spontaneous preterm birth fetal | case-control study | African | A | G | 66 | 183 | 1.232 (0.819, 1.853) |
| 18276608 | Velez | 2008 | rs7549338 | USA | Spontaneous preterm birth maternal | case-control study | Caucasian | G | C | 145 | 198 | 1.402 (1.027, 1.914) |
| 18276608 | Velez | 2008 | rs7549338 | USA | Spontaneous preterm birth fetal | case-control study | Caucasian | G | C | 140 | 179 | 0.916 (0.669, 1.255) |
| 18276608 | Velez | 2008 | rs7549338 | African-American | Spontaneous preterm birth maternal | case-control study | African | G | C | 76 | 191 | 0.567 (0.38, 0.845) |
| 18276608 | Velez | 2008 | rs7549338 | African-American | Spontaneous preterm birth fetal | case-control study | African | G | C | 66 | 183 | 1.247 (0.829, 1.878) |
| 18276608 | Velez | 2008 | rs7526293 | USA | Spontaneous preterm birth maternal | case-control study | Caucasian | C | T | 145 | 198 | 0.742 (0.51, 1.077) |
| 18276608 | Velez | 2008 | rs7526293 | USA | Spontaneous preterm birth fetal | case-control study | Caucasian | C | T | 140 | 179 | 0.996 (0.683, 1.451) |
| 18276608 | Velez | 2008 | rs7526293 | African-American | Spontaneous preterm birth maternal | case-control study | African | C | T | 76 | 191 | 0.991 (0.677, 1.45) |
| 18276608 | Velez | 2008 | rs7526293 | African-American | Spontaneous preterm birth fetal | case-control study | African | C | T | 66 | 183 | 1.029 (0.687, 1.54) |
| 18276608 | Velez | 2008 | rs6687726 | USA | Spontaneous preterm birth maternal | case-control study | Caucasian | G | A | 145 | 198 | 1.279 (0.94, 1.74) |
| 18276608 | Velez | 2008 | rs6687726 | USA | Spontaneous preterm birth fetal | case-control study | Caucasian | A | G | 140 | 179 | 0.924 (0.675, 1.264) |
| 18276608 | Velez | 2008 | rs6687726 | African-American | Spontaneous preterm birth maternal | case-control study | African | G | A | 76 | 191 | 1.49 (1.015, 2.188) |
| 18276608 | Velez | 2008 | rs6687726 | African-American | Spontaneous preterm birth fetal | case-control study | African | G | A | 66 | 183 | 0.919 (0.61, 1.383) |
| 18276608 | Velez | 2008 | rs6427641 | USA | Spontaneous preterm birth maternal | case-control study | Caucasian | A | G | 145 | 198 | 1.009 (0.744, 1.368) |
| 18276608 | Velez | 2008 | rs6427641 | USA | Spontaneous preterm birth fetal | case-control study | Caucasian | A | G | 140 | 179 | 1.038 (0.757, 1.423) |
| 18276608 | Velez | 2008 | rs6427641 | African-American | Spontaneous preterm birth maternal | case-control study | African | A | G | 76 | 191 | 1.429 (0.957, 2.133) |
| 18276608 | Velez | 2008 | rs6427641 | African-American | Spontaneous preterm birth fetal | case-control study | African | A | G | 66 | 183 | 1.184 (0.78, 1.797) |
| 18276608 | Velez | 2008 | rs4845625 | USA | Spontaneous preterm birth maternal | case-control study | Caucasian | T | C | 145 | 198 | 0.716 (0.525, 0.976) |
| 18276608 | Velez | 2008 | rs4845625 | USA | Spontaneous preterm birth fetal | case-control study | Caucasian |  | T | 140 | 179 | 0.856 (0.625, 1.171) |
| 18276608 | Velez | 2008 | rs4845625 | African-American | Spontaneous preterm birth maternal | case-control study | African |  | T | 76 | 191 | 0.61 (0.405, 0.918) |
| 18276608 | Velez | 2008 | rs4845625 | African-American | Spontaneous preterm birth fetal | case-control study | African | C | T | 66 | 183 | 1.269 (0.83, 1.94) |
| 18276608 | Velez | 2008 | rs4845623 | USA | Spontaneous preterm birth maternal | case-control study | Caucasian | A | G | 145 | 198 | 0.852 (0.628, 1.155) |
| 18276608 | Velez | 2008 | rs4845623 | African-American | Spontaneous preterm birth fetal | case-control study | African | A | G | 66 | 183 | 1.291 (0.866, 1.925) |
| 18276608 | Velez | 2008 | rs4845622 | USA | Spontaneous preterm birth maternal | case-control study | Caucasian | A | C | 145 | 198 | 0.876 (0.645, 1.189) |
| 18276608 | Velez | 2008 | rs4845622 | USA | Spontaneous preterm birth fetal | case-control study | Caucasian | A | C | 140 | 179 | 1.038 (0.754, 1.429) |
| 18276608 | Velez | 2008 | rs4845622 | African-American | Spontaneous preterm birth maternal | case-control study | African | A | C | 76 | 191 | 1.641 (0.94, 2.864) |
| 18276608 | Velez | 2008 | rs4845622 | African-American | Spontaneous preterm birth fetal | case-control study | African | A | C | 66 | 183 | 0.718 (0.397, 1.298) |
| 18276608 | Velez | 2008 | rs4845618 | USA | Spontaneous preterm birth maternal | case-control study | Caucasian | T | G | 145 | 198 | 1.279 (0.94, 1.74) |
| 18276608 | Velez | 2008 | rs4845618 | African-American | Spontaneous preterm birth fetal | case-control study | African | T | G | 66 | 183 | 0.75 (0.503, 1.119) |
| 18276608 | Velez | 2008 | rs4845374 | USA | Spontaneous preterm birth maternal | case-control study | Caucasian | T | A | 145 | 198 | 1.478 (0.971, 2.25) |
| 18276608 | Velez | 2008 | rs4845374 | USA | Spontaneous preterm birth fetal | case-control study | Caucasian | T | A | 140 | 179 | 0.92 (0.59, 1.434) |
| 18276608 | Velez | 2008 | rs4845374 | African-American | Spontaneous preterm birth maternal | case-control study | African | T | A | 76 | 191 | 1.234 (0.795, 1.916) |
| 18276608 | Velez | 2008 | rs4845374 | African-American | Spontaneous preterm birth fetal | case-control study | African | T | A | 66 | 183 | 1.053 (0.664, 1.67) |
| 18276608 | Velez | 2008 | rs4601580 | USA | Spontaneous preterm birth maternal | case-control study | Caucasian | T | A | 145 | 198 | 1.083 (0.795, 1.474) |
| 18276608 | Velez | 2008 | rs4601580 | USA | Spontaneous preterm birth fetal | case-control study | Caucasian | T | A | 140 | 179 | 1.091 (0.797, 1.493) |
| 18276608 | Velez | 2008 | rs4601580 | African-American | Spontaneous preterm birth maternal | case-control study | African | T | A | 76 | 191 | 0.934 (0.638, 1.366) |
| 18276608 | Velez | 2008 | rs4601580 | African-American | Spontaneous preterm birth fetal | case-control study | African | T | A | 66 | 183 | 0.953 (0.636, 1.428) |
| 18276608 | Velez | 2008 | rs4553185 | USA | Spontaneous preterm birth maternal | case-control study | Caucasian | T | C | 145 | 198 | 1.336 (0.981, 1.82) |
| 18276608 | Velez | 2008 | rs4553185 | USA | Spontaneous preterm birth fetal | case-control study | Caucasian | T | C | 140 | 179 | 0.925 (0.676, 1.267) |
| 18276608 | Velez | 2008 | rs4553185 | African-American | Spontaneous preterm birth maternal | case-control study | African | T | C | 76 | 191 | 1.714 (1.169, 2.514) |
| 18276608 | Velez | 2008 | rs4553185 | African-American | Spontaneous preterm birth fetal | case-control study | African | T | C | 66 | 183 | 0.854 (0.57, 1.28) |
| 18276608 | Velez | 2008 | rs4537545 | USA | Spontaneous preterm birth maternal | case-control study | Caucasian | C | T | 145 | 198 | 0.888 (0.655, 1.204) |
| 18276608 | Velez | 2008 | rs4537545 | USA | Spontaneous preterm birth fetal | case-control study | Caucasian | C | T | 140 | 179 | 1.085 (0.789, 1.493) |
| 18276608 | Velez | 2008 | rs4537545 | African-American | Spontaneous preterm birth maternal | case-control study | African | C | T | 76 | 191 | 0.667 (0.451, 0.985) |
| 18276608 | Velez | 2008 | rs4537545 | African-American | Spontaneous preterm birth fetal | case-control study | African | C | T | 66 | 183 | 1.166 (0.778, 1.75) |
| 18276608 | Velez | 2008 | rs4329505 | USA | Spontaneous preterm birth maternal | case-control study | Caucasian | T | C | 145 | 198 | 1.375 (0.899, 2.104) |
| 18276608 | Velez | 2008 | rs4329505 | USA | Spontaneous preterm birth fetal | case-control study | Caucasian | T | C | 140 | 179 | 1.003 (0.639, 1.575) |
| 18276608 | Velez | 2008 | rs4329505 | African-American | Spontaneous preterm birth fetal | case-control study | African | T | C | 66 | 183 | 1.164 (0.761, 1.78) |
| 18276608 | Velez | 2008 | rs4075015 | USA | Spontaneous preterm birth fetal | case-control study | Caucasian | A | T | 140 | 179 | 1.048 (0.762, 1.441) |
| 18276608 | Velez | 2008 | rs4075015 | African-American | Spontaneous preterm birth fetal | case-control study | African | A | T | 66 | 183 | 0.991 (0.538, 1.824) |
| 18276608 | Velez | 2008 | rs4072391 | USA | Spontaneous preterm birth maternal | case-control study | Caucasian | C | T | 145 | 198 | 0.681 (0.463, 1.003) |
| 18276608 | Velez | 2008 | rs4072391 | African-American | Spontaneous preterm birth fetal | case-control study | African | C | T | 66 | 183 | 1.166 (0.756, 1.799) |
| 18276608 | Velez | 2008 | rs2229238 | USA | Spontaneous preterm birth maternal | case-control study | Caucasian | C | T | 145 | 198 | 0.681 (0.463, 1.003) |
| 18276608 | Velez | 2008 | rs2229238 | USA | Spontaneous preterm birth fetal | case-control study | Caucasian | C | T | 140 | 179 | 1.061 (0.725, 1.552) |
| 18276608 | Velez | 2008 | rs2229238 | African-American | Spontaneous preterm birth maternal | case-control study | African | C | T | 76 | 191 | 0.795 (0.499, 1.266) |
| 18276608 | Velez | 2008 | rs2229238 | African-American | Spontaneous preterm birth fetal | case-control study | African | C | T | 66 | 183 | 0.991 (0.613, 1.601) |
| 18276608 | Velez | 2008 | rs1552481 | USA | Spontaneous preterm birth maternal | case-control study | Caucasian | T | C | 145 | 198 | 0.15 (0.008, 2.799) |
| 18276608 | Velez | 2008 | rs1552481 | African-American | Spontaneous preterm birth maternal | case-control study | African | T | C | 76 | 191 | 0.935 (0.578, 1.513) |
| 18276608 | Velez | 2008 | rs1552481 | African-American | Spontaneous preterm birth fetal | case-control study | African | T | C | 66 | 183 | 1.1 (0.648, 1.868) |
| 18276608 | Velez | 2008 | rs1386821 | USA | Spontaneous preterm birth maternal | case-control study | Caucasian | A | C | 145 | 198 | 0.838 (0.578, 1.214) |
| 18276608 | Velez | 2008 | rs1386821 | USA | Spontaneous preterm birth fetal | case-control study | Caucasian | A | C | 140 | 179 | 0.864 (0.579, 1.289) |
| 18276608 | Velez | 2008 | rs1386821 | African-American | Spontaneous preterm birth maternal | case-control study | African | A | C | 76 | 191 | 1.25 (0.676, 2.314) |
| 18276608 | Velez | 2008 | rs1386821 | African-American | Spontaneous preterm birth fetal | case-control study | African | A | C | 66 | 183 | 0.877 (0.464, 1.657) |
| 18276608 | Velez | 2008 | rs11265618 | USA | Spontaneous preterm birth maternal | case-control study | Caucasian | C | T | 145 | 198 | 7.767 (5.345, 11.287) |
| 18276608 | Velez | 2008 | rs11265618 | USA | Spontaneous preterm birth fetal | case-control study | Caucasian | C | T | 140 | 179 | 0.785 (0.51, 1.208) |
| 18276608 | Velez | 2008 | rs11265618 | African-American | Spontaneous preterm birth maternal | case-control study | African | C | T | 76 | 191 | 0.902 (0.606, 1.344) |
| 18276608 | Velez | 2008 | rs11265618 | African-American | Spontaneous preterm birth fetal | case-control study | African | C | T | 66 | 183 | 1.009 (0.663, 1.536) |
| 18276608 | Velez | 2008 | rs11265610 | USA | Spontaneous preterm birth maternal | case-control study | Caucasian | T | C | 145 | 198 | 1.024 (0.228, 4.612) |
| 18276608 | Velez | 2008 | rs11265610 | USA | Spontaneous preterm birth fetal | case-control study | Caucasian | T | C | 140 | 179 | 9.043 (0.465, 175.8) |
| 18276608 | Velez | 2008 | rs11265610 | African-American | Spontaneous preterm birth maternal | case-control study | African | T | C | 76 | 191 | 0.851 (0.558, 1.297) |
| 18276608 | Velez | 2008 | rs11265610 | African-American | Spontaneous preterm birth fetal | case-control study | African | T | C | 66 | 183 | 0.894 (0.562, 1.42) |
| 18276608 | Velez | 2008 | rs10752641 | USA | Spontaneous preterm birth maternal | case-control study | Caucasian | C | G | 145 | 198 | 0.758 (0.528, 1.087) |
| 18276608 | Velez | 2008 | rs10752641 | USA | Spontaneous preterm birth fetal | case-control study | Caucasian | C | G | 140 | 179 | 0.995 (0.697, 1.421) |
| 18276608 | Velez | 2008 | rs10752641 | African-American | Spontaneous preterm birth maternal | case-control study | African | C | G | 76 | 191 | 0.76 (0.511, 1.129) |
| 18276608 | Velez | 2008 | rs10752641 | African-American | Spontaneous preterm birth fetal | case-control study | African | C | G | 66 | 183 | 0.89 (0.59, 1.341) |
| 17984249 | Bustamante | 2007 | rs4845617 | Spanish | Obesity | cohort study | Caucasian | G | A | 86 | 376 | 0.686 (0.481, 0.976) |
| 17984249 | Bustamante | 2007 | rs3887104 | Spanish | Obesity | cohort study | Caucasian | C | T | 83 | 385 | 0.947 (0.578, 1.55) |
| 17984249 | Bustamante | 2007 | rs2228145 | Spanish | Obesity | case-control study | Caucasian | A | C | 82 | 402 | 0.839 (0.595, 1.182) |
| 17898129 | Qi | 2007 | rs6684439 | USA | Type 2 Diabetes | case-control study | Caucasian | C | T | 672 | 1058 | 0.95 (0.824, 1.094) |
| 17898129 | Qi | 2007 | rs4845622 | USA | Type 2 Diabetes | case-control study | Caucasian | A | C | 672 | 1058 | 1.018 (0.884, 1.171) |
| 17898129 | Qi | 2007 | rs4845618 | USA | Type 2 Diabetes | case-control study | Caucasian | T | G | 672 | 1058 | 0.903 (0.784, 1.039) |
| 17898129 | Qi | 2007 | rs4845617 | USA | Type 2 Diabetes | case-control study | Caucasian | G | A | 672 | 1058 | 0.911 (0.79, 1.049) |
| 17898129 | Qi | 2007 | rs4329505 | USA | Type 2 Diabetes | case-control study | Caucasian | T | C | 672 | 1058 | 1.154 (0.956, 1.393) |
| 17898129 | Qi | 2007 | rs4240872 | USA | Type 2 Diabetes | case-control study | Caucasian | T | C | 672 | 1058 | 0.922 (0.783, 1.085) |
| 17898129 | Qi | 2007 | rs4075015 | USA | Type 2 Diabetes | case-control study | Caucasian | A | T | 672 | 1058 | 1.043 (0.907, 1.2) |
| 17898129 | Qi | 2007 | rs2229238 | USA | Type 2 Diabetes | case-control study | Caucasian | C | T | 672 | 1058 | 0.931 (0.781, 1.111) |
| 17898129 | Qi | 2007 | rs2228145 | USA | Type 2 Diabetes | case-control study | Caucasian | A | C | 672 | 1058 | 1.013 (0.879, 1.167) |
| 17898129 | Qi | 2007 | rs12083537 | USA | Type 2 Diabetes | case-control study | Caucasian | T | C | 672 | 1058 | 0.894 (0.755, 1.058) |
| 17119059 | Cozen | 2006 | rs2228145 | USA | Multiple Myeloma | case-control study | Caucasian | A | C | 150 | 126 | 1.100 (0.730, 1.655) |
| 15561970 | Hamid | 2004 | rs2228145 | Denmark | Type 2 Diabetes | case-control study | Caucasian | A | C | 1349 | 4596 | 0.885 (0.811, 0.967) |

**Supplementary Table 2.** Characteristics of the included studies on variants in the *IL6R* gene and levels of categorical phenotypes.

| **PMID** | **Author** | **Year** | **SNP** | **Country** | **Ethnicity** | **Phenotypes** | **Major-allele** | **Minor-allele** | **Individuals** | **Beta** | **SE-Beta** |
| --- | --- | --- | --- | --- | --- | --- | --- | --- | --- | --- | --- |
| 33096487 | Bowker | 2020 | rs2228145 | European | Caucasian | 2-hour glucose | A | C | 42854 | -0.008 | 0.0071 |
| 33096487 | Bowker | 2020 | rs2228145 | European | Caucasian | fasting plasma glucose | A | C | 133010 | 0.001 | 0.0031 |
| 33096487 | Bowker | 2020 | rs2228145 | European | Caucasian | Non-fasted glucose | A | C | 355817 | -0.0003 | 0.0022 |
| 33096487 | Bowker | 2020 | rs2228145 | European | Caucasian | HbA1c | A | C | 479942 | -0.007 | 0.0020 |
| 33096487 | Bowker | 2020 | rs2228145 | European | Caucasian | Fasting insulin | A | C | 108557 | 0.001 | 0.0041 |
| 33096487 | Bowker | 2020 | rs2228145 | European | Caucasian | BMI | A | C | 772066 | 0.003 | 0.0015 |
| 33096487 | Bowker | 2020 | rs2228145 | European | Caucasian | Hip circumference | A | C | 604143 | 0.002 | 0.0020 |
| 33096487 | Bowker | 2020 | rs2228145 | European | Caucasian | Waist circumference | A | C | 615305 | 0.0007 | 0.0017 |
| 33096487 | Bowker | 2020 | rs2228145 | European | Caucasian | Waist-to-hip ratio adjusted for BMI | A | C | 625123 | -0.004 | 0.0005 |
| 33096487 | Bowker | 2020 | rs2228145 | European | Caucasian | Waist-to-hip ratio | A | C | 602940 | -0.001 | 0.0015 |
| 32382712 | Pedersen | 2020 | rs4537545 | Danes | Caucasian | CRP level | C | T | 55469 | -0.137 | 0.0082 |
| 32328834 | Zhang | 2020 | rs2228145 | Mix | Caucasian | sIL-6R level | A | C | 1650 | 0.295 | 0.0150 |
| 32223966 | Yuan | 2020 | rs4129267 | European | Caucasian | HDL cholesterol level | C | T | 188577 | 0.003 | 0.0040 |
| 32223966 | Yuan | 2020 | rs4129267 | European | Caucasian | LDL cholesterol level | C | T | 188577 | 0.003 | 0.0040 |
| 32223966 | Yuan | 2020 | rs4129267 | European | Caucasian | total cholesterol level | C | T | 188577 | 0.005 | 0.0040 |
| 32223966 | Yuan | 2020 | rs4129267 | European | Caucasian | Triglycerides level | C | T | 188577 | 0.002 | 0.0030 |
| 31900081 | Lin | 2020 | rs2228145 | European | Caucasian | sIL-6R level | A | C | 1454 | 0.295 | 0.0150 |
| 31552141 | Rosa | 2019 | rs79925547 | mix | Caucasian | sIL-6R level | C | T | 3301 | 0.752 | 0.1155 |
| 31552141 | Rosa | 2019 | rs79778789 | mix | Caucasian | sIL-6R level | G | A | 3301 | -0.7852 | 0.0887 |
| 31552141 | Rosa | 2019 | rs79219014 | mix | Caucasian | sIL-6R level | G | T | 3301 | 0.7582 | 0.0767 |
| 31552141 | Rosa | 2019 | rs77741705 | mix | Caucasian | sIL-6R level | G | C | 3301 | 0.5205 | 0.0941 |
| 31552141 | Rosa | 2019 | rs7525477 | mix | Caucasian | sIL-6R level | G | A | 3301 | -0.3502 | 0.0261 |
| 31552141 | Rosa | 2019 | rs4129267 | mix | Caucasian | sIL-6R level | C | T | 3301 | 1.1148 | 0.0157 |
| 31552141 | Rosa | 2019 | rs35717427 | mix | Caucasian | sIL-6R level | G | A | 3301 | 0.5238 | 0.0360 |
| 31552141 | Rosa | 2019 | rs147700711 | mix | Caucasian | sIL-6R level | G | T | 3301 | -0.4972 | 0.1190 |
| 31552141 | Rosa | 2019 | rs142712385 | mix | Caucasian | sIL-6R level | T | A | 3301 | -0.2782 | 0.0534 |
| 31552141 | Rosa | 2019 | rs139952834 | mix | Caucasian | sIL-6R level | C | T | 3301 | -0.6506 | 0.1072 |
| 31552141 | Rosa | 2019 | rs113580743 | mix | Caucasian | sIL-6R level | G | A | 3301 | -0.5141 | 0.0605 |
| 31276585 | McGowan | 2019 | rs2228145 | Mix | Caucasian | sIL-6R level | A | C | 1645 | 0.2949 | 0.0148 |
| 30945673 | Prasad | 2019 | rs4129267 | Indians | Other | CRP level | C | T | 4493 | -0.39 | 0.1358 |
| 30657332 | Paige | 2019 | rs2228145 | Mix | Caucasian | abdominal aortic aneurysm growth rate | A | C |  | -0.06 | 0.0612 |
| 30388399 | Ligthart | 2018 | rs61812598 | Mix | Caucasian | CRP Level | G | A | 204402 | -0.094 | 0.0040 |
| 30388399 | Ligthart | 2018 | rs61812598 | Mix | Caucasian | CRP Level | G | A | 204402 | -0.09 | 0.0040 |
| 30388399 | Ligthart | 2018 | rs4845623 | Mix | Caucasian | CRP Level | A | G | 204402 | -0.075 | 0.0001 |
| 30388399 | Ligthart | 2018 | rs4845623 | Mix | Caucasian | CRP Level | A | G | 204402 | -0.096 | 0.0001 |
| 30388399 | Ligthart | 2018 | rs4129267 | Mix | Caucasian | CRP level | C | T | 204402 | -0.088 | 0.0040 |
| 30388399 | Ligthart | 2018 | rs12083537 | Mix | Caucasian | CRP Level | A | G | 204402 | 0.064 | 0.0050 |
| 30090940 | Cai | 2018 | rs2228145 | USA | Mix | CK-MB (creatine kinase–MB) | A | C | 332799 | -0.01 | 0.0036 |
| 30090940 | Cai | 2018 | rs2228145 | USA | Mix | CRP level | A | C | 332799 | -0.06 | 0.0102 |
| 30090940 | Cai | 2018 | rs2228145 | USA | Mix | Pro–B-type natriuretic peptide | A | C | 332799 | -0.02 | 0.0153 |
| 30090940 | Cai | 2018 | rs2228145 | USA | Mix | Troponin I | A | C | 332799 | -0.04 | 0.0102 |
| 29775600 | Parisinos | 2018 | rs2228145 | UK | Caucasian | IL-6 level | A | C | 1,650 | 0.1362 | 0.0176 |
| 29775600 | Parisinos | 2018 | rs2228145 | UK | Caucasian | sIL-6R level | A | C | 1,650 | 0.2949 | 0.0148 |
| 29403010 | Kanai | 2018 | rs12133641 | Japan | Asian | CRP Level | A | G | 75391 | -0.04067 | 0.0053 |
| 29197507 | Khandaker | 2018 | rs2228145 | England | Caucasian | CRP level | A | C | 5086 | -0.11 | 0.0270 |
| 29197507 | Khandaker | 2018 | rs2228145 | England | Caucasian | IL-6 level | A | C | 5076 | 0.182 | 0.0190 |
| 28974776 | Chan | 2017 | rs7553796 | Germany | Caucasian | IL6R level | C | A | 198 | 0.28 | 0.0571 |
| 28974776 | Chan | 2017 | rs7514452 | Germany | Caucasian | IL6R level | T | C | 198 | 0.32 | 0.0656 |
| 28974776 | Chan | 2017 | rs4845625 | Germany | Caucasian | IL6R level | C | T | 198 | 0.31 | 0.0552 |
| 28974776 | Chan | 2017 | rs4537545 | Germany | Caucasian | IL6R level | C | T | 198 | -0.42 | 0.0518 |
| 28974776 | Chan | 2017 | rs4240872 | Germany | Caucasian | IL6R level | T | C | 198 | 0.3 | 0.0635 |
| 28974776 | Chan | 2017 | rs4129267 | Germany | Caucasian | IL6R level | C | T | 331 | -0.44 | 0.0529 |
| 28974776 | Chan | 2017 | rs2229238 | Germany | Caucasian | IL6R level | C | T | 198 | 0.32 | 0.0666 |
| 28769070 | Arguinano | 2017 | rs4845625 | France | Caucasian | ApoB level | C | T | 368 | 0.044 | 0.0170 |
| 28769070 | Arguinano | 2017 | rs4845625 | France | Caucasian | CRP level | C | T | 368 | 0.182 | 0.0710 |
| 28769070 | Arguinano | 2017 | rs4845625 | France | Caucasian | LDL cholesterol level | C | T | 368 | 0.052 | 0.0190 |
| 28769070 | Arguinano | 2017 | rs4845625 | France | Caucasian | LDL cholesterol level | C | T | 995 | 0.024 | 0.1100 |
| 28769070 | Arguinano | 2017 | rs4845625 | France | Caucasian | Total cholesterol level | C | T | 368 | 0.021 | 0.0120 |
| 28769070 | Arguinano | 2017 | rs4845625 | France | Caucasian | Total cholesterol level | C | T | 995 | 0.019 | 0.0070 |
| 28769070 | Arguinano | 2017 | rs4845625 | France | Caucasian | Triglycerides level | C | T | 368 | 0.013 | 0.0380 |
| 28769070 | Arguinano | 2017 | rs4537545 | France | Caucasian | ApoB level | C | T | 368 | -0.011 | 0.5280 |
| 28769070 | Arguinano | 2017 | rs4537545 | France | Caucasian | CRP level | C | T | 368 | -0.189 | 0.0720 |
| 28769070 | Arguinano | 2017 | rs4537545 | France | Caucasian | LDL cholesterol level | C | T | 368 | -0.021 | 0.2960 |
| 28769070 | Arguinano | 2017 | rs4537545 | France | Caucasian | LDL cholesterol level | C | T | 995 | -0.01 | 0.3640 |
| 28769070 | Arguinano | 2017 | rs4537545 | France | Caucasian | Total cholesterol level | C | T | 368 | 0 | 0.9860 |
| 28769070 | Arguinano | 2017 | rs4537545 | France | Caucasian | Total cholesterol level | C | T | 995 | -0.01 | 0.1900 |
| 28769070 | Arguinano | 2017 | rs4537545 | France | Caucasian | Triglycerides level | C | T | 368 | -0.005 | 0.0880 |
| 26946122 | Soerensen | 2016 | rs6689393 | Denmark | Caucasian | Hand grip strength | G | A | 1174 | 0.2510672 | 0.2442 |
| 26946122 | Soerensen | 2016 | rs6689393 | Denmark | Caucasian | Mini-Mental State Examination | G | A | 1174 | -0.01678 | 0.0857 |
| 26946122 | Soerensen | 2016 | rs6689393 | Denmark | Caucasian | Cognitive composite score | G | A | 1174 | 0.2022 | 0.1565 |
| 26946122 | Soerensen | 2016 | rs6689393 | Denmark | Caucasian | activity of daily living disability score | G | A | 1174 | 0.0706277 | 0.0874 |
| 26946122 | Soerensen | 2016 | rs6689393 | Denmark | Caucasian | activity of daily living strength score | G | A | 1174 | 0.0699513 | 0.0813 |
| 26946122 | Soerensen | 2016 | rs6689393 | Denmark | Caucasian | Self-rated health | G | A | 1174 | 0.0828694 | 0.0839 |
| 26946122 | Soerensen | 2016 | rs6684439 | Denmark | Caucasian | Hand grip strength | C | T | 1174 | -0.173123 | 0.2434 |
| 26946122 | Soerensen | 2016 | rs6684439 | Denmark | Caucasian | Mini-Mental State Examination | C | T | 1174 | 0.1356312 | 0.0854 |
| 26946122 | Soerensen | 2016 | rs6684439 | Denmark | Caucasian | Cognitive composite score | C | T | 1174 | -0.07318 | 0.1567 |
| 26946122 | Soerensen | 2016 | rs6684439 | Denmark | Caucasian | activity of daily living disability score | C | T | 1174 | 0.0258642 | 0.0871 |
| 26946122 | Soerensen | 2016 | rs6684439 | Denmark | Caucasian | activity of daily living strength score | C | T | 1174 | 0.0011829 | 0.0815 |
| 26946122 | Soerensen | 2016 | rs6684439 | Denmark | Caucasian | Self-rated health | C | T | 1174 | 0.0180801 | 0.0841 |
| 26946122 | Soerensen | 2016 | rs4845626 | Denmark | Caucasian | Hand grip strength | G | T | 1174 | -0.128088 | 0.2936 |
| 26946122 | Soerensen | 2016 | rs4845626 | Denmark | Caucasian | Mini-Mental State Examination | G | T | 1174 | -0.146543 | 0.1047 |
| 26946122 | Soerensen | 2016 | rs4845626 | Denmark | Caucasian | Cognitive composite score | G | T | 1174 | -0.2753 | 0.1894 |
| 26946122 | Soerensen | 2016 | rs4845626 | Denmark | Caucasian | activity of daily living disability score | G | T | 1174 | 0.0142329 | 0.1051 |
| 26946122 | Soerensen | 2016 | rs4845626 | Denmark | Caucasian | activity of daily living strength score | G | T | 1174 | 0.0211917 | 0.0976 |
| 26946122 | Soerensen | 2016 | rs4845626 | Denmark | Caucasian | Self-rated health | G | T | 1174 | -0.006746 | 0.1022 |
| 26946122 | Soerensen | 2016 | rs4601580 | Denmark | Caucasian | Hand grip strength | A | T | 1174 | -0.118554 | 0.2405 |
| 26946122 | Soerensen | 2016 | rs4601580 | Denmark | Caucasian | Mini-Mental State Examination | A | T | 1174 | 0.0389431 | 0.0836 |
| 26946122 | Soerensen | 2016 | rs4601580 | Denmark | Caucasian | Cognitive composite score | A | T | 1174 | 0.1391 | 0.1518 |
| 26946122 | Soerensen | 2016 | rs4601580 | Denmark | Caucasian | activity of daily living disability score | A | T | 1174 | 0.0122286 | 0.0849 |
| 26946122 | Soerensen | 2016 | rs4601580 | Denmark | Caucasian | activity of daily living strength score | A | T | 1174 | -0.055904 | 0.0796 |
| 26946122 | Soerensen | 2016 | rs4601580 | Denmark | Caucasian | Self-rated health | A | T | 1174 | 0.017372 | 0.0811 |
| 26946122 | Soerensen | 2016 | rs4240872 | Denmark | Caucasian | Hand grip strength | T | C | 1174 | -0.024487 | 0.2725 |
| 26946122 | Soerensen | 2016 | rs4240872 | Denmark | Caucasian | Mini-Mental State Examination | T | C | 1174 | -0.040068 | 0.0966 |
| 26946122 | Soerensen | 2016 | rs4240872 | Denmark | Caucasian | Cognitive composite score | T | C | 1174 | 0.01884 | 0.1743 |
| 26946122 | Soerensen | 2016 | rs4240872 | Denmark | Caucasian | activity of daily living disability score | T | C | 1174 | 0.0866501 | 0.0966 |
| 26946122 | Soerensen | 2016 | rs4240872 | Denmark | Caucasian | activity of daily living strength score | T | C | 1174 | 0.076042 | 0.0899 |
| 26946122 | Soerensen | 2016 | rs4240872 | Denmark | Caucasian | Self-rated health | T | C | 1174 | 0.1281556 | 0.0936 |
| 26946122 | Soerensen | 2016 | rs4075015 | Denmark | Caucasian | Hand grip strength | A | T | 1174 | -0.274261 | 0.2497 |
| 26946122 | Soerensen | 2016 | rs4075015 | Denmark | Caucasian | Mini-Mental State Examination | A | T | 1174 | -0.059286 | 0.0875 |
| 26946122 | Soerensen | 2016 | rs4075015 | Denmark | Caucasian | Cognitive composite score | A | T | 1174 | -0.07279 | 0.1599 |
| 26946122 | Soerensen | 2016 | rs4075015 | Denmark | Caucasian | activity of daily living disability score | A | T | 1174 | -0.006126 | 0.0882 |
| 26946122 | Soerensen | 2016 | rs4075015 | Denmark | Caucasian | activity of daily living strength score | A | T | 1174 | 0.0465801 | 0.0825 |
| 26946122 | Soerensen | 2016 | rs4075015 | Denmark | Caucasian | Self-rated health | A | T | 1174 | 0.0927484 | 0.0863 |
| 26946122 | Soerensen | 2016 | rs2229238 | Denmark | Caucasian | Hand grip strength | C | T | 1174 | 0.0644024 | 0.3031 |
| 26946122 | Soerensen | 2016 | rs2229238 | Denmark | Caucasian | Mini-Mental State Examination | C | T | 1174 | -0.046518 | 0.1065 |
| 26946122 | Soerensen | 2016 | rs2229238 | Denmark | Caucasian | Cognitive composite score | C | T | 1174 | 0.0436 | 0.1923 |
| 26946122 | Soerensen | 2016 | rs2229238 | Denmark | Caucasian | activity of daily living disability score | C | T | 1174 | 0.0583333 | 0.1069 |
| 26946122 | Soerensen | 2016 | rs2229238 | Denmark | Caucasian | activity of daily living strength score | C | T | 1174 | 0.0974981 | 0.0998 |
| 26946122 | Soerensen | 2016 | rs2229238 | Denmark | Caucasian | Self-rated health | C | T | 1174 | 0.0665535 | 0.1030 |
| 26946122 | Soerensen | 2016 | rs12083537 | Denmark | Caucasian | Hand grip strength | A | G | 1174 | 0.4573808 | 0.2970 |
| 26946122 | Soerensen | 2016 | rs12083537 | Denmark | Caucasian | Mini-Mental State Examination | A | G | 1174 | -0.105182 | 0.1037 |
| 26946122 | Soerensen | 2016 | rs12083537 | Denmark | Caucasian | Cognitive composite score | A | G | 1174 | -0.02757 | 0.1906 |
| 26946122 | Soerensen | 2016 | rs12083537 | Denmark | Caucasian | activity of daily living disability score | A | G | 1174 | -0.118746 | 0.1060 |
| 26946122 | Soerensen | 2016 | rs12083537 | Denmark | Caucasian | activity of daily living strength score | A | G | 1174 | -0.208086 | 0.0999 |
| 26946122 | Soerensen | 2016 | rs12083537 | Denmark | Caucasian | Self-rated health | A | G | 1174 | -0.003006 | 0.1022 |
| 26336855 | Lopez-Lasanta | 2015 | rs6698040 | Spain | Caucasian | severity of joint damage RA | G | A | 527 | -0.79 | 0.4898 |
| 26336855 | Lopez-Lasanta | 2015 | rs4845618 | Spain | Caucasian | severity of joint damage RA | T | G | 527 | -1.09 | 0.3878 |
| 26336855 | Lopez-Lasanta | 2015 | rs4845618 | Spain | Caucasian | severity of joint damage RA | T | G | 527 | -1.01 | 0.3724 |
| 26336855 | Lopez-Lasanta | 2015 | rs4845374 | Spain | Caucasian | severity of joint damage RA | T | A | 527 | 1.14 | 0.4898 |
| 26336855 | Lopez-Lasanta | 2015 | rs4845374 | Spain | Caucasian | severity of joint damage RA | T | A | 705 | 0.95 | 0.4592 |
| 26336855 | Lopez-Lasanta | 2015 | rs4453032 | Spain | Caucasian | severity of joint damage RA | A | G | 527 | 0.25 | 0.3878 |
| 26336855 | Lopez-Lasanta | 2015 | rs4379670 | Spain | Caucasian | severity of joint damage RA | A | T | 527 | -0.64 | 0.5510 |
| 25340798 | Kauwe | 2014 | rs61812598 | USA | Caucasian | sIL-6R level | A | G | 574 | 0.49 | 0.0597 |
| 25187575 | Schick | 2015 | rs2228145 | Europe-America | Caucasian | CRP level | A | C | 6050 | -0.12 | 0.0190 |
| 25187575 | Schick | 2015 | rs2228145 | African-America | African American | CRP level | A | C | 3109 | -0.089 | 0.0400 |
| 24671014 | Hong | 2014 | rs2228145 | Asian | Asian | CRP level | A | C | 748 | -0.127 | 0.0410 |
| 24643644 | Ellis | 2014 | rs4129267 | Europe | Caucasian | CRP level | C | T | 29939 | -0.079 | 0.0080 |
| 24643644 | Ellis | 2014 | rs4129267 | African-America | African American | CRP level | C | T | 7570 | -0.12 | 0.0250 |
| 24643644 | Ellis | 2014 | rs2228145 | African-America | African American | CRP level | A | C | 7570 | -0.12 | 0.0250 |
| 23894628 | Kim | 2013 | rs4129267 | USA | Caucasian | IL6R level | C | T | 521 | 0.115 | 0.0001 |
| 23894628 | Kim | 2013 | rs4129267 | USA | Caucasian | IL6R level | C | T | 59 | 0.181 | 0.0481 |
| 23844046 | Dorajoo | 2013 | rs4537545 | China | Asian | CRP level | C | T | 2434 | -0.053 | 0.0149 |
| 23844046 | Dorajoo | 2013 | rs4537545 | Malays | Asian | CRP level | C | T | 2542 | -0.024 | 0.0153 |
| 23844046 | Dorajoo | 2013 | rs4537545 | Singapore | Asian | CRP level | C | T | 2538 | -0.026 | 0.0138 |
| 23593036 | Ferreira | 2013 | rs4329505 | Mix | Caucasian | sIL-6R level | T | C | 2517 | 0.031 | 0.0030 |
| 23593036 | Ferreira | 2013 | rs4329505 | Mix | Caucasian | sIL-6R level | T | C | 2517 | 0.066 | 0.0090 |
| 23593036 | Ferreira | 2013 | rs1386821 | Mix | Caucasian | sIL-6R level | T | G | 2340 | 0.016 | 0.0030 |
| 23593036 | Ferreira | 2013 | rs1386821 | Mix | Caucasian | sIL-6R level | T | G | 2340 | 0.031 | 0.0070 |
| 23582566 | Esparza-Gordillo | 2013 | rs2228145 | Mix | Caucasian | sIL-6R level | A | C | 105 | 0.204 | 0.0393 |
| 23505291 | Shah | 2013 | rs7553796 | UK | Caucasian | IL-6 level | A | C | 4911 | -0.07 | 0.0100 |
| 23505291 | Shah | 2013 | rs7553796 | UK | Caucasian | CRP level | A | C | 4911 | 0.08 | 0.0200 |
| 23505291 | Shah | 2013 | rs7553796 | UK | Caucasian | IL-6 level | A | C | 3445 | -0.05 | 0.0100 |
| 23505291 | Shah | 2013 | rs7553796 | UK | Caucasian | CRP level | A | C | 3445 | 0.08 | 0.0200 |
| 23505291 | Shah | 2013 | rs7529229 | UK | Caucasian | CRP level | T | C | 4911 | -0.1 | 0.0200 |
| 23505291 | Shah | 2013 | rs7529229 | UK | Caucasian | CRP level | T | C | 3445 | -0.1 | 0.0200 |
| 23505291 | Shah | 2013 | rs7529229 | UK | Caucasian | IL-6 level | T | C | 4911 | 0.09 | 0.0100 |
| 23505291 | Shah | 2013 | rs7529229 | UK | Caucasian | IL-6 level | T | C | 3445 | 0.07 | 0.0100 |
| 23505291 | Shah | 2013 | rs7518199 | UK | Caucasian | IL-6 level | A | C | 4911 | 0.1 | 0.0100 |
| 23505291 | Shah | 2013 | rs7518199 | UK | Caucasian | IL-6 level | A | C | 3445 | 0.06 | 0.0100 |
| 23505291 | Shah | 2013 | rs7518199 | UK | Caucasian | CRP level | C | A | 4911 | -0.1 | 0.0200 |
| 23505291 | Shah | 2013 | rs7518199 | UK | Caucasian | CRP level | C | A | 3445 | -0.1 | 0.0200 |
| 23505291 | Shah | 2013 | rs6667434 | UK | Caucasian | CRP level | G | A | 4911 | -0.08 | 0.0200 |
| 23505291 | Shah | 2013 | rs6667434 | UK | Caucasian | CRP level | G | A | 3445 | -0.08 | 0.0200 |
| 23505291 | Shah | 2013 | rs6667434 | UK | Caucasian | IL-6 level | G | A | 4911 | 0.07 | 0.0100 |
| 23505291 | Shah | 2013 | rs6667434 | UK | Caucasian | IL-6 level | G | A | 3445 | 0.05 | 0.0100 |
| 23505291 | Shah | 2013 | rs4845625 | UK | Caucasian | CRP level | C | T | 4911 | 0.08 | 0.0200 |
| 23505291 | Shah | 2013 | rs4845625 | UK | Caucasian | CRP level | C | T | 3445 | 0.08 | 0.0200 |
| 23505291 | Shah | 2013 | rs4845625 | UK | Caucasian | IL-6 level | C | T | 4911 | -0.07 | 0.0100 |
| 23505291 | Shah | 2013 | rs4845625 | UK | Caucasian | IL-6 level | C | T | 3445 | -0.06 | 0.0100 |
| 23505291 | Shah | 2013 | rs4845618 | UK | Caucasian | CRP level | C | A | 4911 | 0.09 | 0.0200 |
| 23505291 | Shah | 2013 | rs4845618 | UK | Caucasian | CRP level | C | A | 3445 | 0.09 | 0.0200 |
| 23505291 | Shah | 2013 | rs4845618 | UK | Caucasian | IL-6 level | C | A | 4911 | -0.06 | 0.0100 |
| 23505291 | Shah | 2013 | rs4845618 | UK | Caucasian | IL-6 level | C | A | 3445 | -0.05 | 0.0100 |
| 23505291 | Shah | 2013 | rs4845371 | UK | Caucasian | CRP level | C | T | 4911 | -0.08 | 0.0200 |
| 23505291 | Shah | 2013 | rs4845371 | UK | Caucasian | CRP level | C | T | 3445 | -0.08 | 0.0200 |
| 23505291 | Shah | 2013 | rs4845371 | UK | Caucasian | IL-6 level | C | T | 4911 | 0.07 | 0.0100 |
| 23505291 | Shah | 2013 | rs4845371 | UK | Caucasian | IL-6 level | C | T | 3445 | 0.05 | 0.0100 |
| 23505291 | Shah | 2013 | rs4553185 | UK | Caucasian | CRP level | T | C | 4911 | 0.08 | 0.0200 |
| 23505291 | Shah | 2013 | rs4553185 | UK | Caucasian | CRP level | T | C | 3445 | 0.08 | 0.0200 |
| 23505291 | Shah | 2013 | rs4553185 | UK | Caucasian | IL-6 level | T | C | 4911 | -0.07 | 0.0100 |
| 23505291 | Shah | 2013 | rs4553185 | UK | Caucasian | IL-6 level | T | C | 3445 | -0.05 | 0.0100 |
| 23505291 | Shah | 2013 | rs4537545 | UK | Caucasian | CRP level | C | T | 4911 | -0.11 | 0.0200 |
| 23505291 | Shah | 2013 | rs4537545 | UK | Caucasian | CRP level | C | T | 3445 | -0.11 | 0.0200 |
| 23505291 | Shah | 2013 | rs4537545 | UK | Caucasian | IL-6 level | C | T | 4911 | 0.1 | 0.0100 |
| 23505291 | Shah | 2013 | rs4537545 | UK | Caucasian | IL-6 level | C | T | 3445 | 0.07 | 0.0100 |
| 23505291 | Shah | 2013 | rs4509570 | UK | Caucasian | IL-6 level | C | G | 4911 | -0.07 | 0.0100 |
| 23505291 | Shah | 2013 | rs4509570 | UK | Caucasian | CRP level | C | G | 4911 | 0.03 | 0.0300 |
| 23505291 | Shah | 2013 | rs4509570 | UK | Caucasian | IL-6 level | C | G | 3445 | -0.04 | 0.0200 |
| 23505291 | Shah | 2013 | rs4509570 | UK | Caucasian | CRP level | C | G | 3445 | 0.03 | 0.0300 |
| 23505291 | Shah | 2013 | rs4129267 | UK | Caucasian | CRP level | C | T | 4911 | -0.11 | 0.0200 |
| 23505291 | Shah | 2013 | rs4129267 | UK | Caucasian | CRP level | C | T | 3445 | -0.11 | 0.0200 |
| 23505291 | Shah | 2013 | rs4129267 | UK | Caucasian | IL-6 level | C | T | 4911 | 0.09 | 0.0100 |
| 23505291 | Shah | 2013 | rs4129267 | UK | Caucasian | IL-6 level | C | T | 3445 | 0.07 | 0.0100 |
| 23505291 | Shah | 2013 | rs28638007 | UK | Caucasian | IL-6 level | A | G | 4911 | -0.07 | 0.0100 |
| 23505291 | Shah | 2013 | rs28638007 | UK | Caucasian | CRP level | A | G | 4911 | 0.09 | 0.0200 |
| 23505291 | Shah | 2013 | rs28638007 | UK | Caucasian | IL-6 level | A | G | 3445 | -0.06 | 0.0100 |
| 23505291 | Shah | 2013 | rs28638007 | UK | Caucasian | CRP level | A | G | 3445 | 0.09 | 0.0200 |
| 23505291 | Shah | 2013 | rs2228145 | UK | Caucasian | CRP level | A | C | 4911 | -0.11 | 0.0200 |
| 23505291 | Shah | 2013 | rs2228145 | UK | Caucasian | CRP level | A | C | 3445 | -0.11 | 0.0200 |
| 23505291 | Shah | 2013 | rs2228145 | UK | Caucasian | IL-6 level | A | C | 4911 | 0.09 | 0.0100 |
| 23505291 | Shah | 2013 | rs2228145 | UK | Caucasian | IL-6 level | A | C | 3445 | 0.07 | 0.0100 |
| 23094986 | Hsieh | 2012 | rs2228145 | China | Asian | BMI | A | C | 451 | 0.0769611 | 0.2136 |
| 23094986 | Hsieh | 2012 | rs2228145 | China | Asian | BMI | A | C | 474 | 0.3364722 | 0.2181 |
| 23094986 | Hsieh | 2012 | rs2228145 | China | Asian | diastolic blood pressure | A | C | 451 | 0.0769611 | 0.2203 |
| 23094986 | Hsieh | 2012 | rs2228145 | China | Asian | diastolic blood pressure | A | C | 474 | 0.10436 | 0.1965 |
| 23094986 | Hsieh | 2012 | rs2228145 | China | Asian | Fasting glucose | A | C | 451 | -0.174353 | 0.2214 |
| 23094986 | Hsieh | 2012 | rs2228145 | China | Asian | Fasting glucose | A | C | 474 | -0.18633 | 0.1986 |
| 23094986 | Hsieh | 2012 | rs2228145 | China | Asian | HDL cholesterol level | A | C | 451 | -0.18633 | 0.2241 |
| 23094986 | Hsieh | 2012 | rs2228145 | China | Asian | HDL cholesterol level | A | C | 474 | 0.3987761 | 0.1963 |
| 23094986 | Hsieh | 2012 | rs2228145 | China | Asian | systolic blood pressure | A | C | 451 | -0.328504 | 0.2394 |
| 23094986 | Hsieh | 2012 | rs2228145 | China | Asian | systolic blood pressure | A | C | 474 | -0.287682 | 0.2176 |
| 23094986 | Hsieh | 2012 | rs2228145 | China | Asian | Triglycerides level | A | C | 451 | 0.3220835 | 0.1964 |
| 23094986 | Hsieh | 2012 | rs2228145 | China | Asian | Triglycerides level | A | C | 474 | -0.314711 | 0.2438 |
| 23094986 | Hsieh | 2012 | rs2228145 | China | Asian | waist circumference | A | C | 451 | 0.1823216 | 0.2203 |
| 23094986 | Hsieh | 2012 | rs2228145 | China | Asian | waist circumference | A | C | 474 | 0.4574249 | 0.2169 |
| 22939635 | Reiner | 2012 | rs4129267 | African-America | African American | CRP level | C | T | 8280 | -0.11 | 0.0250 |
| 22939635 | Reiner | 2012 | rs4129267 | Hispanic American | Other | CRP level | C | T | 3548 | -0.087 | 0.0250 |
| 22421340 | Swerdlow | 2012 | rs7529229 | Mix | Caucasian | Albumin level | T | C | 5787 | 0.1 | 0.0510 |
| 22421340 | Swerdlow | 2012 | rs7529229 | Mix | Caucasian | AST | T | C | 7201 | 0.006 | 0.0051 |
| 22421340 | Swerdlow | 2012 | rs7529229 | Mix | Caucasian | CRP level | T | C | 76527 | -0.09 | 0.0051 |
| 22421340 | Swerdlow | 2012 | rs7529229 | Mix | Caucasian | fibrinogen level | T | C | 52667 | -0.009 | 0.0015 |
| 22421340 | Swerdlow | 2012 | rs7529229 | Mix | Caucasian | Haemoglobin level | T | C | 17898 | 0.022 | 0.0107 |
| 22421340 | Swerdlow | 2012 | rs7529229 | Mix | Caucasian | HDL cholesterol level | T | C | 105439 | 0.002 | 0.0020 |
| 22421340 | Swerdlow | 2012 | rs7529229 | Mix | Caucasian | IL-6 level | T | C | 29838 | 0.09 | 0.0051 |
| 22421340 | Swerdlow | 2012 | rs7529229 | Mix | Caucasian | LDL cholesterol level | T | C | 97966 | -0.003 | 0.0041 |
| 22421340 | Swerdlow | 2012 | rs7529229 | Mix | Caucasian | Platelets level | T | C | 3274 | -0.76 | 1.8214 |
| 22421340 | Swerdlow | 2012 | rs7529229 | Mix | Caucasian | sIL-6R level | T | C | 1454 | 0.1487 | 0.0091 |
| 22421340 | Swerdlow | 2012 | rs7529229 | Mix | Caucasian | Total cholesterol level | T | C | 114615 | 0.004 | 0.0046 |
| 22421340 | Swerdlow | 2012 | rs7529229 | Mix | Caucasian | Triglycerides level | T | C | 105656 | -0.002 | 0.0020 |
| 22421339 | Sarwar | 2012 | rs2228145 | Mix | Caucasian | BMI | A | C | 122222 | 0.0001 | 0.0010 |
| 22421339 | Sarwar | 2012 | rs2228145 | Mix | Caucasian | CRP level | A | C | 83948 | -0.075 | 0.0082 |
| 22421339 | Sarwar | 2012 | rs2228145 | Mix | Caucasian | Fasting glucose | A | C | 51109 | 0.0002 | 0.0014 |
| 22421339 | Sarwar | 2012 | rs2228145 | Mix | Caucasian | fibrinogen level | A | C | 50353 | -0.01 | 0.0015 |
| 22421339 | Sarwar | 2012 | rs2228145 | Mix | Caucasian | HDL cholesterol level | A | C | 95970 | 0.0007 | 0.0017 |
| 22421339 | Sarwar | 2012 | rs2228145 | Mix | Caucasian | IL-6 level | A | C | 27185 | 0.146 | 0.0194 |
| 22421339 | Sarwar | 2012 | rs2228145 | Mix | Caucasian | LDL cholesterol level | A | C | 80343 | -0.0003 | 0.0017 |
| 22421339 | Sarwar | 2012 | rs2228145 | Mix | Caucasian | sIL-6R level | A | C | 1645 | 0.343 | 0.0199 |
| 22421339 | Sarwar | 2012 | rs2228145 | Mix | Caucasian | smoking | A | C | 54817 | -0.020203 | 0.0130 |
| 22421339 | Sarwar | 2012 | rs2228145 | Mix | Caucasian | systolic blood pressure | A | C | 99577 | -0.001 | 0.0005 |
| 22421339 | Sarwar | 2012 | rs2228145 | Mix | Caucasian | Triglycerides level | A | C | 94473 | -0.003 | 0.0031 |
| 22421339 | Sarwar | 2012 | rs2228145 | Mix | Caucasian | waist circumference | A | C | 81492 | 0.002 | 0.0005 |
| 22291609 | Naitza | 2012 | rs4129267 | Sardinians | Other | IL-6 level | C | T | 5915 | 0.109 | 0.0200 |
| 22228719 | Tabassum | 2012 | rs7514452 | India | Other | BMI | T | C | 830 | 0.13 | 0.0500 |
| 22228719 | Tabassum | 2012 | rs7514452 | India | Other | BMI | T | C | 1399 | 0.009 | 0.0400 |
| 21937998 | Curocichin | 2011 | rs2228145 | Filipino | Asian | CRP level | A | C | 1691 | -0.313 | 0.0910 |
| 21931794 | Mahajan | 2011 | rs7529229 | India | Other | CRP level | T | C | 1042 | -0.14 | 0.0602 |
| 21931794 | Mahajan | 2011 | rs7529229 | India | Other | CRP level | T | C | 1073 | -0.03 | 0.0752 |
| 21931794 | Mahajan | 2011 | rs6667434 | India | Other | CRP level | G | A | 1042 | -0.11 | 0.0585 |
| 21931794 | Mahajan | 2011 | rs6667434 | India | Other | CRP level | G | A | 1073 | -0.09 | 0.0641 |
| 21931794 | Mahajan | 2011 | rs4845622 | India | Other | CRP level | A | C | 1042 | -0.12 | 0.0466 |
| 21931794 | Mahajan | 2011 | rs4845622 | India | Other | CRP level | A | C | 1073 | -0.03 | 0.0942 |
| 21931794 | Mahajan | 2011 | rs4845371 | India | Other | CRP level | C | T | 1042 | -0.11 | 0.0585 |
| 21931794 | Mahajan | 2011 | rs4845371 | India | Other | CRP level | C | T | 1073 | -0.09 | 0.0641 |
| 21931794 | Mahajan | 2011 | rs4129267 | India | Other | CRP level | C | T | 1073 | -0.02 | 0.0751 |
| 21835044 | Chu | 2011 | rs4845623 | China | Asian | Total cholesterol level | A | G | 418 | 0.0953102 | 0.3233 |
| 21835044 | Chu | 2011 | rs4845623 | China | Asian | Triglycerides level | A | G | 418 | 0.3715636 | 0.2711 |
| 21835044 | Chu | 2011 | rs4845623 | China | Asian | HDL cholesterol level | A | G | 418 | -0.020203 | 0.2652 |
| 21835044 | Chu | 2011 | rs4845623 | China | Asian | LDL cholesterol level | A | G | 418 | -0.210721 | 0.3364 |
| 21835044 | Chu | 2011 | rs4845623 | China | Asian | Total cholesterol/HDL-C | A | G | 418 | -0.301105 | 0.3381 |
| 21835044 | Chu | 2011 | rs4845623 | China | Asian | Total cholesterol level | A | G | 441 | -0.040822 | 0.3203 |
| 21835044 | Chu | 2011 | rs4845623 | China | Asian | Triglycerides level | A | G | 441 | -0.562119 | 0.3291 |
| 21835044 | Chu | 2011 | rs4845623 | China | Asian | HDL cholesterol level | A | G | 441 | -0.274437 | 0.3151 |
| 21835044 | Chu | 2011 | rs4845623 | China | Asian | LDL cholesterol level | A | G | 441 | 0.0099503 | 0.3007 |
| 21835044 | Chu | 2011 | rs4845623 | China | Asian | Total cholesterol/HDL-C | A | G | 441 | -0.356675 | 0.3321 |
| 21835044 | Chu | 2011 | rs4845617 | China | Asian | Total cholesterol level | G | A | 418 | -0.094311 | 0.2559 |
| 21835044 | Chu | 2011 | rs4845617 | China | Asian | Triglycerides level | G | A | 418 | -0.083382 | 0.2119 |
| 21835044 | Chu | 2011 | rs4845617 | China | Asian | HDL cholesterol level | G | A | 418 | 0.0198026 | 0.1927 |
| 21835044 | Chu | 2011 | rs4845617 | China | Asian | LDL cholesterol level | G | A | 418 | -0.248461 | 0.2409 |
| 21835044 | Chu | 2011 | rs4845617 | China | Asian | Total cholesterol/HDL-C | G | A | 418 | 0.2151114 | 0.2279 |
| 21835044 | Chu | 2011 | rs4845617 | China | Asian | Total cholesterol level | G | A | 441 | 0.2070142 | 0.2279 |
| 21835044 | Chu | 2011 | rs4845617 | China | Asian | Triglycerides level | G | A | 441 | 0.463734 | 0.2053 |
| 21835044 | Chu | 2011 | rs4845617 | China | Asian | HDL cholesterol level | G | A | 441 | 0.2151114 | 0.2058 |
| 21835044 | Chu | 2011 | rs4845617 | China | Asian | LDL cholesterol level | G | A | 441 | 0.0769611 | 0.2187 |
| 21835044 | Chu | 2011 | rs4845617 | China | Asian | Total cholesterol/HDL-C | G | A | 441 | 0.2390169 | 0.2166 |
| 21835044 | Chu | 2011 | rs2229238 | China | Asian | Total cholesterol level | C | T | 418 | -0.210721 | 0.2657 |
| 21835044 | Chu | 2011 | rs2229238 | China | Asian | Triglycerides level | C | T | 418 | -0.210721 | 0.2327 |
| 21835044 | Chu | 2011 | rs2229238 | China | Asian | HDL cholesterol level | C | T | 418 | -0.174353 | 0.2089 |
| 21835044 | Chu | 2011 | rs2229238 | China | Asian | LDL cholesterol level | C | T | 418 | -0.210721 | 0.2566 |
| 21835044 | Chu | 2011 | rs2229238 | China | Asian | Total cholesterol/HDL-C | C | T | 418 | -0.248461 | 0.2482 |
| 21835044 | Chu | 2011 | rs2229238 | China | Asian | Total cholesterol level | C | T | 441 | 0.1823216 | 0.2277 |
| 21835044 | Chu | 2011 | rs2229238 | China | Asian | Triglycerides level | C | T | 441 | 0.4382549 | 0.2014 |
| 21835044 | Chu | 2011 | rs2229238 | China | Asian | HDL cholesterol level | C | T | 441 | 0.5187938 | 0.2069 |
| 21835044 | Chu | 2011 | rs2229238 | China | Asian | LDL cholesterol level | C | T | 441 | 0.1988509 | 0.2152 |
| 21835044 | Chu | 2011 | rs2229238 | China | Asian | Total cholesterol/HDL-C | C | T | 441 | 0.5988365 | 0.2195 |
| 21835044 | Chu | 2011 | rs2228145 | China | Asian | HDL cholesterol level | A | C | 418 | -0.248461 | 0.2140 |
| 21835044 | Chu | 2011 | rs2228145 | China | Asian | HDL cholesterol level | A | C | 441 | 0.4510756 | 0.2147 |
| 21835044 | Chu | 2011 | rs2228145 | China | Asian | LDL cholesterol level | A | C | 418 | -0.18633 | 0.2608 |
| 21835044 | Chu | 2011 | rs2228145 | China | Asian | LDL cholesterol level | A | C | 441 | 0.0953102 | 0.2242 |
| 21835044 | Chu | 2011 | rs2228145 | China | Asian | Total cholesterol level | A | C | 418 | -0.105361 | 0.2688 |
| 21835044 | Chu | 2011 | rs2228145 | China | Asian | Total cholesterol level | A | C | 441 | 0.1222176 | 0.2373 |
| 21835044 | Chu | 2011 | rs2228145 | China | Asian | Total cholesterol/HDL-C | A | C | 418 | -0.356675 | 0.2532 |
| 21835044 | Chu | 2011 | rs2228145 | China | Asian | Total cholesterol/HDL-C | A | C | 441 | 0.5187938 | 0.2251 |
| 21835044 | Chu | 2011 | rs2228145 | China | Asian | Triglycerides level | A | C | 418 | -0.162519 | 0.2319 |
| 21835044 | Chu | 2011 | rs2228145 | China | Asian | Triglycerides level | A | C | 441 | 0.4574249 | 0.2077 |
| 21647738 | Wu | 2012 | rs4129267 | Filipino | Asian | CRP level | C | T | 1798 | -0.068 | 0.0580 |
| 21300955 | Dehghan | 2011 | rs4129267 | Mix | Caucasian | CRP level | C | T | 66185 | -0.094 | 0.0070 |
| 21300955 | Dehghan | 2011 | rs4129267 | Mix | Caucasian | CRP level | C | T | 16540 | -0.045 | 0.0100 |
| 21196492 | OkadaHum | 2011 | rs2228145 | Japan | Asian | CRP level | A | C | 10112 | -0.038 | 0.0140 |
| 20978265 | Wassel | 2011 | rs7529229 | Europe-America | Caucasian | fibrinogen level | T | C | 23634 | -0.05 | 0.0090 |
| 20978265 | Wassel | 2011 | rs7529229 | African-America | African American | fibrinogen level | T | C | 6657 | -0.012 | 0.0182 |
| 20978265 | Wassel | 2011 | rs7518199 | Europe-America | Caucasian | fibrinogen level | A | C | 23634 | -0.048 | 0.0095 |
| 20978265 | Wassel | 2011 | rs4537545 | Europe-America | Caucasian | fibrinogen level | C | T | 23634 | -0.049 | 0.0094 |
| 20978265 | Wassel | 2011 | rs4129267 | Europe-America | Caucasian | fibrinogen level | C | T | 23634 | -0.046 | 0.0093 |
| 20978265 | Wassel | 2011 | rs2228145 | Europe-America | Caucasian | fibrinogen level | A | C | 23634 | -0.046 | 0.0094 |
| 20978265 | Wassel | 2011 | rs2228145 | African-America | African American | fibrinogen level | A | C | 6657 | -0.06 | 0.0258 |
| 20157327 | Andersson | 2010 | rs952146 | Sweden | Caucasian | fat mass | A | G | 1049 | -0.002 | 0.0430 |
| 20157327 | Andersson | 2010 | rs6684439 | Sweden | Caucasian | fat mass | C | T | 1049 | 0.022 | 0.0430 |
| 20157327 | Andersson | 2010 | rs4553185 | Sweden | Caucasian | fat mass | T | C | 1049 | -0.053 | 0.0430 |
| 20157327 | Andersson | 2010 | rs4240872 | Sweden | Caucasian | fat mass | T | C | 1049 | -0.062 | 0.0490 |
| 20157327 | Andersson | 2010 | rs4129267 | Sweden | Caucasian | fat mass | C | T | 1049 | 0.039 | 0.0430 |
| 20157327 | Andersson | 2010 | rs4075015 | Sweden | Caucasian | fat mass | A | T | 1049 | -0.092 | 0.0420 |
| 20157327 | Andersson | 2010 | rs2229238 | Sweden | Caucasian | fat mass | C | T | 1049 | 0.057 | 0.0530 |
| 20157327 | Andersson | 2010 | rs2228145 | Sweden | Caucasian | fat mass | A | C | 1049 | 0.04 | 0.0430 |
| 20157327 | Andersson | 2010 | rs1386821 | Sweden | Caucasian | fat mass | T | G | 1049 | -0.069 | 0.0560 |
| 20157327 | Andersson | 2010 | rs11265618 | Sweden | Caucasian | fat mass | C | T | 1049 | 0.004 | 0.0570 |
| 20031577 | Danik | 2009 | rs6684439 | Mix | Caucasian | fibrinogen level | G | A | 17686 | -0.671173 | 0.2365 |
| 20031577 | Danik | 2009 | rs4845623 | Mix | Caucasian | fibrinogen level | A | G | 17686 | -4.55 | 0.7825 |
| 20031577 | Danik | 2009 | rs4537545 | Mix | Caucasian | fibrinogen level | C | T | 17686 | -0.699838 | 0.2360 |
| 20031577 | Danik | 2009 | rs4129267 | Mix | Caucasian | fibrinogen level | C | T | 17686 | -5.3 | 0.7889 |
| 20031577 | Danik | 2009 | rs2228145 | Mix | Caucasian | CRP level | A | C | 17686 | -0.109 | 0.0111 |
| 20031577 | Danik | 2009 | rs2228145 | Mix | Caucasian | fibrinogen level | A | C | 17686 | -0.069984 | 0.0236 |
| 19567438 | Elliott | 2009 | rs4537545 | Mix | Caucasian | CRP level | C | T | 31582 | -0.115 | 0.0153 |
| 19567438 | Elliott | 2009 | rs4537545 | Mix | Caucasian | BMI | C | T | 13615 | 0.01 | 0.0612 |
| 19567438 | Elliott | 2009 | rs4537545 | Mix | Caucasian | diastolic blood pressure | C | T | 13615 | 0.14 | 0.1276 |
| 19567438 | Elliott | 2009 | rs4537545 | Mix | Caucasian | HDL cholesterol level | C | T | 13615 | -0.1 | 0.1531 |
| 19567438 | Elliott | 2009 | rs4537545 | Mix | Caucasian | LDL cholesterol level | C | T | 13615 | 0.41 | 0.4388 |
| 19567438 | Elliott | 2009 | rs4537545 | Mix | Caucasian | systolic blood pressure | C | T | 13615 | 0.07 | 0.2143 |
| 19567438 | Elliott | 2009 | rs4537545 | Mix | Caucasian | Total cholesterol level | C | T | 13615 | 0 | 0.5204 |
| 19567438 | Elliott | 2009 | rs4537545 | Mix | Caucasian | Triglycerides level | C | T | 13615 | -2.21 | 1.3265 |
| 19567438 | Elliott | 2009 | rs4537545 | Mix | Caucasian | Type 2 Diabetes | C | T | 13615 | 0.94 | 0.0408 |
| 19567438 | Elliott | 2009 | rs4537545 | Mix | Caucasian | Weight | C | T | 13615 | 0.19 | 0.1786 |
| 19249341 | Walston | 2009 | rs7518199 | USA | Caucasian | IL-6 level | A | C | 4190 | 0.101 | 0.0130 |
| 19249341 | Walston | 2009 | rs4845618 | USA | Caucasian | IL-6 level | T | G | 4190 | -0.078 | 0.0130 |
| 19249341 | Walston | 2009 | rs4553185 | USA | Caucasian | IL-6 level | T | C | 4190 | -0.082 | 0.0130 |
| 19249341 | Walston | 2009 | rs4537545 | USA | Caucasian | IL-6 level | C | T | 4190 | 0.114 | 0.0130 |
| 19249341 | Walston | 2009 | rs4072391 | USA | Caucasian | IL-10 level | C | T | 4190 | -0.076 | 0.0170 |
| 19249341 | Walston | 2009 | rs2229238 | USA | Caucasian | IL-11 level | C | T | 4190 | -0.079 | 0.0170 |
| 18439548 | Ridker | 2008 | rs4129267 | USA | Caucasian | CRP level | C | T | 4418 | -0.096 | 0.0217 |
| 18439548 | Ridker | 2008 | rs4129267 | USA | Caucasian | CRP level | C | T | 1927 | -0.117 | 0.0328 |
| 18439548 | Ridker | 2008 | rs2228145 | USA | Caucasian | CRP level | A | C | 4418 | -0.095 | 0.0216 |
| 18439548 | Ridker | 2008 | rs2228145 | USA | Caucasian | CRP level | A | C | 1927 | -0.119 | 0.0328 |
| 17671508 | Rafiq | 2007 | rs4537545 | Mix | Caucasian | Adiponectin level | C | T | 1273 | -0.06 | 0.0306 |
| 17671508 | Rafiq | 2007 | rs4537545 | Mix | Caucasian | BMI | C | T | 1273 | 0.04 | 0.1735 |
| 17671508 | Rafiq | 2007 | rs4537545 | Mix | Caucasian | Fasting glucose level | C | T | 1273 | -0.02 | 0.0102 |
| 17671508 | Rafiq | 2007 | rs4537545 | Mix | Caucasian | Fasting insulina level | C | T | 1273 | -0.04 | 0.0204 |
| 17671508 | Rafiq | 2007 | rs4537545 | Mix | Caucasian | HDL cholesterol level | C | T | 1273 | -0.008 | 0.0112 |
| 17671508 | Rafiq | 2007 | rs4537545 | Mix | Caucasian | LDL cholesterol level | C | T | 1273 | 0.17 | 1.4439 |
| 17671508 | Rafiq | 2007 | rs4537545 | Mix | Caucasian | Total cholesterol level | C | T | 1273 | -1.33 | 1.6429 |
| 17671508 | Rafiq | 2007 | rs4537545 | Mix | Caucasian | Triglycerides level | C | T | 1273 | -0.03 | 0.0204 |
| 17671508 | Rafiq | 2007 | rs4537545 | Mix | Caucasian | Waist/hip ratio | C | T | 1273 | 0.006 | 0.0027 |

**Supplementary Table 3.** Characteristics of the included studies on variants in the *IL6R* gene and mean levels of continuous quantitative phenotypes.

| **PMID** | **Author** | **Year** | **SNP** | **Phenotypes** | **Ethnicity** | **Population** | **Major** | **Minor** | **Genotypes** | | | **Major-Major** | | **Major-Minor** | | **Minor-Minor** | |
| --- | --- | --- | --- | --- | --- | --- | --- | --- | --- | --- | --- | --- | --- | --- | --- | --- | --- |
|  |  |  |  |  |  |  |  |  | **Major-Major** | **Major-Minor** | **Minor-Minor** | **Mean** | **SD** | **Mean** | **SD** | **Mean** | **SD** |
| 29197507 | Khandaker | 2018 | rs2228145 | CRP level | Caucasian | healthy individuals | A | C | 1176 | 1621 | 601 | 0.83 | 2.95 | 0.84 | 2.70 | 0.62 | 2.55 |
| 29197507 | Khandaker | 2018 | rs2228145 | IL-6 level | Caucasian | healthy individuals | A | C | 1176 | 1621 | 601 | 1.13 | 1.42 | 1.32 | 1.69 | 1.46 | 1.62 |
| 26997259 | Wang | 2016 | rs2228145 | sIL-6R level | Asian | asthma patients | A | C | 21 | 34 | 11 | 111.10 | 34.19 | 129.60 | 49.44 | 159.90 | 42.52 |
| 26997259 | Wang | 2016 | rs2228145 | FEF 25–75% | Asian | asthma patients | A | C | 151 | 170 | 42 | 55.94 | 82.47 | 54.20 | 32.73 | 45.12 | 31.53 |
| 26997259 | Wang | 2016 | rs2228145 | FEV1 | Asian | asthma patients | A | C | 163 | 178 | 45 | 76.01 | 24.36 | 76.21 | 24.11 | 66.16 | 26.95 |
| 26997259 | Wang | 2016 | rs2228145 | FEV1/FVC | Asian | asthma patients | A | C | 163 | 178 | 45 | 72.16 | 14.20 | 73.67 | 14.56 | 71.00 | 18.91 |
| 26997259 | Wang | 2016 | rs2228145 | FVC | Asian | asthma patients | A | C | 179 | 194 | 61 | 90.45 | 62.43 | 85.59 | 19.11 | 76.01 | 24.17 |
| 26997259 | Wang | 2016 | rs2228145 | IgE level | Asian | asthma patients | A | C | 166 | 181 | 47 | 472.80 | 572.30 | 540.60 | 859.70 | 792.40 | 1102.00 |
| 26997259 | Wang | 2016 | rs2228145 | Peripheral eosinophil | Asian | asthma patients | A | C | 166 | 180 | 47 | 0.39 | 0.39 | 0.43 | 0.33 | 0.34 | 0.24 |
| 26997259 | Wang | 2016 | rs2228145 | Peripheral eosinophil | Asian | asthma patients | A | C | 166 | 181 | 47 | 4.57 | 3.24 | 5.30 | 4.12 | 4.32 | 3.23 |
| 26997259 | Wang | 2016 | rs12083537 | sIL-6R level | Asian | asthma patients | A | G | 49 | 16 | 1 | 128.70 | 39.38 | 129.80 | 73.16 | 107.61 | 0.00 |
| 26997259 | Wang | 2016 | rs12083537 | IgE level | Asian | asthma patients | A | G | 332 | 58 | 4 | 540.90 | 600.50 | 542.60 | 629.70 | 634.90 | 232.20 |
| 26997259 | Wang | 2016 | rs12083537 | Peripheral eosinophil | Asian | asthma patients | A | G | 331 | 58 | 4 | 0.40 | 0.30 | 0.45 | 0.55 | 0.33 | 0.05 |
| 26997259 | Wang | 2016 | rs12083537 | Peripheral eosinophil | Asian | asthma patients | A | G | 332 | 58 | 4 | 4.92 | 3.77 | 4.71 | 3.31 | 3.79 | 1.19 |
| 26997259 | Wang | 2016 | rs12083537 | FEV1 | Asian | asthma patients | A | G | 325 | 57 | 4 | 74.92 | 24.71 | 77.46 | 23.79 | 42.10 | 15.11 |
| 26997259 | Wang | 2016 | rs12083537 | FVC | Asian | asthma patients | A | G | 325 | 57 | 4 | 88.59 | 47.86 | 88.02 | 18.62 | 65.10 | 16.98 |
| 26997259 | Wang | 2016 | rs12083537 | FEV1/FVC | Asian | asthma patients | A | G | 325 | 57 | 4 | 72.71 | 14.96 | 73.91 | 14.91 | 57.05 | 9.16 |
| 26997259 | Wang | 2016 | rs12083537 | FEF 25–75% | Asian | asthma patients | A | G | 306 | 53 | 4 | 53.97 | 62.31 | 56.00 | 32.76 | 17.78 | 9.58 |
| 26238946 | Abe | 2015 | rs4845625 | Triglycerides level | Asian | healthy individuals | C | T | 828 | 1475 | 679 | 1.61 | 1.10 | 1.61 | 1.18 | 1.52 | 1.02 |
| 25524550 | Horibe | 2015 | rs4845625 | creatinine level | Asian | healthy individuals | C | T | 592 | 1127 | 528 | 131.60 | 185.70 | 113.00 | 145.00 | 99.70 | 106.60 |
| 25524550 | Horibe | 2015 | rs4845625 | estimated glomerular filtration rate level | Asian | healthy individuals | C | T | 592 | 1127 | 528 | 61.10 | 34.30 | 61.90 | 29.80 | 66.90 | 38.00 |
| 24791950 | van Dongen | 2014 | rs2228145 | sIL-6R level | Caucasian | healthy individuals | A | C | 1843 | 2390 | 747 | 5.70 | 2.32 | 4.42 | 0.06 | 3.24 | 0.88 |
| 24791950 | van Dongen | 2014 | rs2228145 | sIL-6R level | Caucasian | healthy individuals | A | C | 2390 | 747 | 4980 | 5.70 | 2.32 | 4.42 | 0.06 | 3.24 | 0.88 |
| 24717336 | Wypasek | 2014 | rs2228145 | BMI | Caucasian | aortic stenosis patients | A | C | 124 | 105 | 55 | 27.70 | 4.20 | 27.50 | 3.70 | 27.30 | 4.50 |
| 24717336 | Wypasek | 2014 | rs2228145 | Glucose | Caucasian | aortic stenosis patients | A | C | 124 | 105 | 55 | 5.60 | 1.10 | 5.70 | 1.40 | 5.40 | 1.20 |
| 24717336 | Wypasek | 2014 | rs2228145 | HDL cholesterol level | Caucasian | aortic stenosis patients | A | C | 124 | 105 | 55 | 1.30 | 0.40 | 1.30 | 0.30 | 1.40 | 0.40 |
| 24717336 | Wypasek | 2014 | rs2228145 | LDL cholesterol level | Caucasian | aortic stenosis patients | A | C | 124 | 105 | 55 | 2.80 | 1.10 | 2.90 | 1.10 | 3.10 | 1.30 |
| 24717336 | Wypasek | 2014 | rs2228145 | Total cholesterol level | Caucasian | aortic stenosis patients | A | C | 124 | 105 | 55 | 4.60 | 1.40 | 4.60 | 1.50 | 4.70 | 1.40 |
| 24717336 | Wypasek | 2014 | rs2228145 | Triglycerides level | Caucasian | aortic stenosis patients | A | C | 124 | 105 | 55 | 1.60 | 0.90 | 1.40 | 0.70 | 1.50 | 1.20 |
| 24717336 | Wypasek | 2014 | rs2228145 | Creatinine | Caucasian | aortic stenosis patients | A | C | 124 | 105 | 55 | 88.4 | 39.4 | 80.8 | 21.7 | 82.9 | 24.7 |
| 24717336 | Wypasek | 2014 | rs2228145 | fibrinogen level | Caucasian | aortic stenosis patients | A | C | 124 | 105 | 55 | 3.4 | 1.1 | 3.4 | 1.1 | 3.2 | 1.3 |
| 23891823 | Méndez | 2013 | rs2228145 | BMD at LS | Other | obesity females | A | C | 32 | 95 | 53 | 0.94 | 0.12 | 0.95 | 0.13 | 0.94 | 0.14 |
| 23891823 | Méndez | 2013 | rs2228145 | BMD at TH | Other | obesity females | A | C | 32 | 95 | 53 | 1.02 | 0.15 | 1.01 | 0.16 | 1.04 | 0.13 |
| 23891823 | Méndez | 2013 | rs2228145 | BMD at FN | Other | obesity females | A | C | 32 | 95 | 53 | 0.80 | 0.10 | 1.02 | 0.15 | 0.81 | 0.12 |
| 23479153 | Vargas | 2013 | rs2228145 | diastolic blood pressure | Other | healthy individuals | A | C | 28 | 36 | 13 | 77.60 | 7.26 | 80.50 | 9.75 | 83.10 | 11.15 |
| 23479153 | Vargas | 2013 | rs2228145 | diastolic blood pressure | Other | metabolic syndrome patients | A | C | 28 | 36 | 13 | 79.40 | 7.34 | 83.80 | 12.44 | 81.00 | 11.82 |
| 23479153 | Vargas | 2013 | rs2228145 | Glucose | Other | healthy individuals | A | C | 28 | 36 | 13 | 95.20 | 15.58 | 101.60 | 21.55 | 94.20 | 14.90 |
| 23479153 | Vargas | 2013 | rs2228145 | Glucose | Other | metabolic syndrome patients | A | C | 28 | 36 | 13 | 110.00 | 49.63 | 109.90 | 33.11 | 95.80 | 15.51 |
| 23479153 | Vargas | 2013 | rs2228145 | HDL cholesterol level | Other | healthy individuals | A | C | 28 | 36 | 13 | 44.20 | 9.25 | 43.20 | 9.80 | 45.30 | 12.18 |
| 23479153 | Vargas | 2013 | rs2228145 | HDL cholesterol level | Other | metabolic syndrome patients | A | C | 28 | 36 | 13 | 45.60 | 11.72 | 43.60 | 9.91 | 43.90 | 10.20 |
| 23479153 | Vargas | 2013 | rs2228145 | systolic blood pressure | Other | healthy individuals | A | C | 28 | 36 | 13 | 120.10 | 9.75 | 124.40 | 13.72 | 132.00 | 16.19 |
| 23479153 | Vargas | 2013 | rs2228145 | systolic blood pressure | Other | metabolic syndrome patients | A | C | 28 | 36 | 13 | 125.50 | 14.70 | 132.80 | 12.59 | 136.00 | 22.78 |
| 23479153 | Vargas | 2013 | rs2228145 | Triglycerides level | Other | healthy individuals | A | C | 28 | 36 | 13 | 118.40 | 58.06 | 206.90 | 128.18 | 173.40 | 91.08 |
| 23479153 | Vargas | 2013 | rs2228145 | Triglycerides level | Other | metabolic syndrome patients | A | C | 28 | 36 | 13 | 177.30 | 95.12 | 233.60 | 123.03 | 189.40 | 55.84 |
| 23479153 | Vargas | 2013 | rs2228145 | waist circumference | Other | healthy individuals | A | C | 28 | 36 | 13 | 96.80 | 8.91 | 103.90 | 10.47 | 103.50 | 6.76 |
| 23479153 | Vargas | 2013 | rs2228145 | waist circumference | Other | metabolic syndrome patients | A | C | 28 | 36 | 13 | 102.20 | 7.23 | 108.80 | 10.33 | 106.80 | 6.16 |
| 22642608 | Perez-Bravo | 2012 | rs2228145 | BMI | Other | Women with Type 1 Diabetes | A | C | 22 | 96 | 27 | 22.90 | 4.50 | 22.10 | 4.40 | 21.50 | 4.10 |
| 22642608 | Perez-Bravo | 2012 | rs2228145 | Age of diagnosis | Other | Women with Type 1 Diabetes | A | C | 22 | 96 | 27 | 9.91 | 7.92 | 10.19 | 7.70 | 9.67 | 7.53 |
| 22642608 | Perez-Bravo | 2012 | rs2228145 | HbA1c | Other | Women with Type 1 Diabetes | A | C | 22 | 96 | 27 | 8.37 | 1.40 | 8.66 | 1.62 | 9.29 | 2.18 |
| 22642608 | Perez-Bravo | 2012 | rs2228145 | Insulin dose | Other | Women with Type 1 Diabetes | A | C | 22 | 96 | 27 | 1.06 | 0.38 | 0.93 | 0.33 | 1.01 | 0.33 |
| 21879314 | Sasayama | 2012 | rs2228145 | full scale intelligence quotient | Asian | healthy individuals | A | C | 186 | 298 | 92 | 113.10 | 11.60 | 110.80 | 11.80 | 111.40 | 12.60 |
| 21879314 | Sasayama | 2012 | rs2228145 | performance intelligence quotient | Asian | healthy individuals | A | C | 186 | 298 | 92 | 111.40 | 12.10 | 110.90 | 11.80 | 111.50 | 12.70 |
| 21879314 | Sasayama | 2012 | rs2228145 | Performance subtests Block design | Asian | healthy individuals | A | C | 186 | 298 | 92 | 12.30 | 2.70 | 12.10 | 3.20 | 12.00 | 2.80 |
| 21879314 | Sasayama | 2012 | rs2228145 | Performance subtests Digit symbol | Asian | healthy individuals | A | C | 186 | 298 | 92 | 13.30 | 2.80 | 13.10 | 2.50 | 13.80 | 2.80 |
| 21879314 | Sasayama | 2012 | rs2228145 | verbal intelligence quotient | Asian | healthy individuals | A | C | 186 | 298 | 92 | 112.40 | 12.10 | 109.00 | 12.80 | 109.60 | 12.40 |
| 21879314 | Sasayama | 2012 | rs2228145 | Verbal subtests Arithmetic | Asian | healthy individuals | A | C | 186 | 298 | 92 | 11.30 | 2.90 | 11.00 | 3.00 | 10.90 | 3.10 |
| 21879314 | Sasayama | 2012 | rs2228145 | Verbal subtests Comprehension | Asian | healthy individuals | A | C | 186 | 298 | 92 | 11.70 | 2.80 | 11.50 | 2.80 | 11.50 | 2.90 |
| 21879314 | Sasayama | 2012 | rs2228145 | Verbal subtests Digit span | Asian | healthy individuals | A | C | 186 | 298 | 92 | 11.60 | 3.00 | 11.20 | 2.80 | 11.70 | 2.60 |
| 21879314 | Sasayama | 2012 | rs2228145 | Verbal subtests Information | Asian | healthy individuals | A | C | 186 | 298 | 92 | 11.70 | 2.60 | 11.00 | 2.80 | 11.00 | 2.80 |
| 21879314 | Sasayama | 2012 | rs2228145 | Verbal subtests Similarities | Asian | healthy individuals | A | C | 186 | 298 | 92 | 12.80 | 2.10 | 12.20 | 2.40 | 12.30 | 2.40 |
| 21879314 | Sasayama | 2012 | rs2228145 | Verbal subtests Vocabulary | Asian | healthy individuals | A | C | 186 | 298 | 92 | 12.10 | 2.60 | 11.40 | 2.70 | 11.50 | 2.90 |
| 21835044 | Chu | 2011 | rs2228145 | HDL cholesterol level | Asian | healthy individuals | A | C | 161 | 202 | 55 | 48.90 | 10.60 | 50.20 | 12.50 | 50.60 | 10.70 |
| 21835044 | Chu | 2011 | rs2228145 | HDL cholesterol level | Asian | healthy individuals | A | C | 148 | 216 | 77 | 52.80 | 11.70 | 51.60 | 11.80 | 49.80 | 10.20 |
| 21835044 | Chu | 2011 | rs2228145 | LDL cholesterol level | Asian | healthy individuals | A | C | 161 | 202 | 55 | 80.40 | 20.20 | 80.90 | 18.70 | 83.90 | 18.20 |
| 21835044 | Chu | 2011 | rs2228145 | LDL cholesterol level | Asian | healthy individuals | A | C | 148 | 216 | 77 | 84.70 | 18.70 | 86.70 | 20.90 | 89.20 | 20.30 |
| 21835044 | Chu | 2011 | rs2228145 | Total cholesterol level | Asian | healthy individuals | A | C | 161 | 202 | 55 | 158.80 | 28.60 | 159.60 | 26.00 | 161.30 | 25.90 |
| 21835044 | Chu | 2011 | rs2228145 | Total cholesterol level | Asian | healthy individuals | A | C | 148 | 216 | 77 | 166.80 | 26.90 | 168.60 | 27.60 | 171.40 | 29.30 |
| 21835044 | Chu | 2011 | rs2228145 | Triglycerides level | Asian | healthy individuals | A | C | 161 | 202 | 55 | 69.90 | 33.50 | 70.30 | 33.80 | 58.70 | 21.20 |
| 21835044 | Chu | 2011 | rs2228145 | Triglycerides level | Asian | healthy individuals | A | C | 148 | 216 | 77 | 66.60 | 24.80 | 70.50 | 30.30 | 73.90 | 38.00 |
| 21835044 | Chu | 2011 | rs2228145 | LDL-C/HDL-C | Asian | healthy individuals | A | C | 161 | 202 | 55 | 1.73 | 0.60 | 1.73 | 0.64 | 1.72 | 0.51 |
| 21835044 | Chu | 2011 | rs2228145 | LDL-C/HDL-C | Asian | healthy individuals | A | C | 148 | 216 | 77 | 1.67 | 0.48 | 1.77 | 0.61 | 1.86 | 0.54 |
| 21835044 | Chu | 2011 | rs2228145 | Total cholesterol/HDL-C | Asian | healthy individuals | A | C | 161 | 202 | 55 | 3.38 | 0.89 | 3.35 | 0.93 | 3.28 | 0.71 |
| 21835044 | Chu | 2011 | rs2228145 | Total cholesterol/HDL-C | Asian | healthy individuals | A | C | 148 | 216 | 77 | 3.26 | 0.66 | 86.70 | 20.90 | 3.56 | 0.84 |
| 21835044 | Chu | 2011 | rs2228145 | Triglycerides/HDL-C | Asian | healthy individuals | A | C | 161 | 202 | 55 | 1.57 | 1.05 | 1.56 | 1.05 | 1.24 | 0.62 |
| 21835044 | Chu | 2011 | rs2228145 | Triglycerides/HDL-C | Asian | healthy individuals | A | C | 148 | 216 | 77 | 1.34 | 0.61 | 1.51 | 0.99 | 1.62 | 1.18 |
| 21835044 | Chu | 2011 | rs4845623 | LDL-C/HDL-C | Asian | healthy individuals | A | G | 290 | 116 | 12 | 1.74 | 0.59 | 1.73 | 0.65 | 1.31 | 0.36 |
| 21835044 | Chu | 2011 | rs4845623 | Triglycerides/HDL-C | Asian | healthy individuals | A | G | 290 | 116 | 12 | 1.51 | 0.98 | 1.61 | 1.11 | 1.05 | 0.46 |
| 21835044 | Chu | 2011 | rs4845623 | LDL-C/HDL-C | Asian | healthy individuals | A | G | 314 | 115 | 12 | 1.78 | 0.58 | 1.68 | 0.51 | 1.87 | 0.46 |
| 21835044 | Chu | 2011 | rs4845623 | Triglycerides/HDL-C | Asian | healthy individuals | A | G | 314 | 115 | 12 | 1.53 | 1.01 | 1.34 | 0.66 | 1.17 | 0.40 |
| 21835044 | Chu | 2011 | rs4845623 | Total cholesterol level | Asian | healthy individuals | A | G | 290 | 116 | 12 | 160.00 | 27.20 | 158.80 | 26.80 | 155.30 | 26.00 |
| 21835044 | Chu | 2011 | rs4845623 | Triglycerides level | Asian | healthy individuals | A | G | 290 | 116 | 12 | 68.00 | 32.00 | 71.10 | 33.80 | 59.30 | 30.10 |
| 21835044 | Chu | 2011 | rs4845623 | HDL cholesterol level | Asian | healthy individuals | A | G | 290 | 116 | 12 | 49.70 | 11.60 | 49.30 | 11.40 | 56.70 | 11.30 |
| 21835044 | Chu | 2011 | rs4845623 | LDL cholesterol level | Asian | healthy individuals | A | G | 290 | 116 | 12 | 81.80 | 19.20 | 80.40 | 19.40 | 72.00 | 16.50 |
| 21835044 | Chu | 2011 | rs4845623 | Total cholesterol/HDL-C | Asian | healthy individuals | A | G | 290 | 116 | 12 | 3.36 | 0.86 | 3.38 | 0.96 | 2.80 | 0.48 |
| 21835044 | Chu | 2011 | rs4845623 | Total cholesterol level | Asian | healthy individuals | A | G | 314 | 115 | 12 | 167.30 | 27.90 | 170.50 | 27.30 | 178.40 | 23.70 |
| 21835044 | Chu | 2011 | rs4845623 | Triglycerides level | Asian | healthy individuals | A | G | 314 | 115 | 12 | 71.10 | 31.70 | 67.50 | 26.80 | 58.40 | 15.30 |
| 21835044 | Chu | 2011 | rs4845623 | HDL cholesterol level | Asian | healthy individuals | A | G | 314 | 115 | 12 | 50.70 | 11.00 | 54.20 | 12.80 | 51.70 | 10.00 |
| 21835044 | Chu | 2011 | rs4845623 | LDL cholesterol level | Asian | healthy individuals | A | G | 314 | 115 | 12 | 86.00 | 20.70 | 86.80 | 18.90 | 93.10 | 14.20 |
| 21835044 | Chu | 2011 | rs4845623 | Total cholesterol/HDL-C | Asian | healthy individuals | A | G | 314 | 115 | 12 | 3.43 | 0.85 | 3.27 | 0.73 | 3.54 | 0.63 |
| 21835044 | Chu | 2011 | rs4845617 | LDL-C/HDL-C | Asian | healthy individuals | G | A | 123 | 199 | 96 | 1.73 | 0.57 | 1.70 | 0.95 | 1.77 | 0.69 |
| 21835044 | Chu | 2011 | rs4845617 | Triglycerides/HDL-C | Asian | healthy individuals | G | A | 123 | 199 | 96 | 1.57 | 0.94 | 1.52 | 1.02 | 1.47 | 1.08 |
| 21835044 | Chu | 2011 | rs4845617 | LDL-C/HDL-C | Asian | healthy individuals | G | A | 130 | 211 | 100 | 1.72 | 0.50 | 1.74 | 0.60 | 1.82 | 0.55 |
| 21835044 | Chu | 2011 | rs4845617 | Triglycerides/HDL-C | Asian | healthy individuals | G | A | 130 | 211 | 100 | 1.37 | 0.72 | 1.47 | 1.01 | 1.59 | 0.96 |
| 21835044 | Chu | 2011 | rs4845617 | Total cholesterol level | Asian | healthy individuals | G | A | 123 | 199 | 96 | 158.70 | 25.90 | 161.40 | 28.40 | 156.70 | 25.40 |
| 21835044 | Chu | 2011 | rs4845617 | Triglycerides level | Asian | healthy individuals | G | A | 123 | 199 | 96 | 70.10 | 29.90 | 70.20 | 34.40 | 63.50 | 31.30 |
| 21835044 | Chu | 2011 | rs4845617 | HDL cholesterol level | Asian | healthy individuals | G | A | 123 | 199 | 96 | 49.00 | 11.00 | 50.70 | 11.70 | 48.80 | 12.00 |
| 21835044 | Chu | 2011 | rs4845617 | LDL cholesterol level | Asian | healthy individuals | G | A | 123 | 199 | 96 | 80.50 | 18.20 | 81.70 | 19.60 | 80.60 | 19.70 |
| 21835044 | Chu | 2011 | rs4845617 | Total cholesterol/HDL-C | Asian | healthy individuals | G | A | 123 | 199 | 96 | 3.38 | 0.84 | 3.32 | 0.85 | 3.39 | 1.01 |
| 21835044 | Chu | 2011 | rs4845617 | Total cholesterol level | Asian | healthy individuals | G | A | 130 | 211 | 100 | 167.10 | 26.00 | 168.70 | 28.50 | 169.80 | 28.20 |
| 21835044 | Chu | 2011 | rs4845617 | Triglycerides level | Asian | healthy individuals | G | A | 130 | 211 | 100 | 66.80 | 27.00 | 69.60 | 31.00 | 74.00 | 32.10 |
| 21835044 | Chu | 2011 | rs4845617 | HDL cholesterol level | Asian | healthy individuals | G | A | 130 | 211 | 100 | 51.70 | 11.20 | 52.50 | 12.20 | 49.90 | 10.30 |
| 21835044 | Chu | 2011 | rs4845617 | LDL cholesterol level | Asian | healthy individuals | G | A | 130 | 211 | 100 | 85.30 | 18.30 | 86.70 | 21.00 | 87.40 | 20.60 |
| 21835044 | Chu | 2011 | rs4845617 | Total cholesterol/HDL-C | Asian | healthy individuals | G | A | 130 | 211 | 100 | 3.33 | 0.70 | 3.36 | 0.89 | 3.52 | 0.80 |
| 21835044 | Chu | 2011 | rs2229238 | LDL-C/HDL-C | Asian | healthy individuals | C | T | 169 | 192 | 57 | 1.71 | 0.61 | 1.74 | 0.62 | 1.75 | 0.57 |
| 21835044 | Chu | 2011 | rs2229238 | Triglycerides/HDL-C | Asian | healthy individuals | C | T | 169 | 192 | 57 | 1.57 | 1.04 | 1.56 | 1.06 | 1.27 | 0.63 |
| 21835044 | Chu | 2011 | rs2229238 | LDL-C/HDL-C | Asian | healthy individuals | C | T | 156 | 202 | 83 | 1.66 | 0.48 | 1.78 | 0.61 | 1.86 | 0.54 |
| 21835044 | Chu | 2011 | rs2229238 | Triglycerides/HDL-C | Asian | healthy individuals | C | T | 156 | 202 | 83 | 1.33 | 0.61 | 1.52 | 1.01 | 1.61 | 1.15 |
| 21835044 | Chu | 2011 | rs2229238 | Total cholesterol level | Asian | healthy individuals | C | T | 169 | 192 | 57 | 158.30 | 28.90 | 160.00 | 25.70 | 161.20 | 25.60 |
| 21835044 | Chu | 2011 | rs2229238 | Triglycerides level | Asian | healthy individuals | C | T | 169 | 192 | 57 | 70.20 | 33.30 | 70.00 | 34.10 | 59.50 | 21.50 |
| 21835044 | Chu | 2011 | rs2229238 | HDL cholesterol level | Asian | healthy individuals | C | T | 169 | 192 | 57 | 49.30 | 10.90 | 50.10 | 12.30 | 50.10 | 11.00 |
| 21835044 | Chu | 2011 | rs2229238 | LDL cholesterol level | Asian | healthy individuals | C | T | 169 | 192 | 57 | 49.30 | 10.90 | 81.50 | 18.10 | 84.00 | 18.40 |
| 21835044 | Chu | 2011 | rs2229238 | Total cholesterol/HDL-C | Asian | healthy individuals | C | T | 169 | 192 | 57 | 3.35 | 0.90 | 3.36 | 0.91 | 3.33 | 0.78 |
| 21835044 | Chu | 2011 | rs2229238 | Total cholesterol level | Asian | healthy individuals | C | T | 156 | 202 | 83 | 166.70 | 27.20 | 1.51 | 0.99 | 171.50 | 30.20 |
| 21835044 | Chu | 2011 | rs2229238 | Triglycerides level | Asian | healthy individuals | C | T | 156 | 202 | 83 | 66.30 | 24.60 | 70.90 | 30.90 | 73.60 | 36.90 |
| 21835044 | Chu | 2011 | rs2229238 | HDL cholesterol level | Asian | healthy individuals | C | T | 156 | 202 | 83 | 52.90 | 11.90 | 51.50 | 11.70 | 49.80 | 10.40 |
| 21835044 | Chu | 2011 | rs2229238 | LDL cholesterol level | Asian | healthy individuals | C | T | 156 | 202 | 83 | 84.50 | 18.90 | 86.70 | 20.80 | 89.20 | 20.50 |
| 21835044 | Chu | 2011 | rs2229238 | Total cholesterol/HDL-C | Asian | healthy individuals | C | T | 156 | 202 | 83 | 3.25 | 0.66 | 3.43 | 0.90 | 89.20 | 20.50 |
| 21552154 | Huuskonen | 2011 | rs4537545 | BMI | Caucasian | healthy males | C | T | 384 | 370 | 75 | 24.60 | 3.40 | 25.00 | 4.10 | 25.60 | 4.30 |
| 21552154 | Huuskonen | 2011 | rs4537545 | Body mass | Caucasian | healthy males | C | T | 384 | 370 | 75 | 79.60 | 12.30 | 81.50 | 14.30 | 82.10 | 14.60 |
| 21552154 | Huuskonen | 2011 | rs4537545 | Lean body mass | Caucasian | healthy males | C | T | 384 | 370 | 75 | 65.00 | 7.40 | 65.80 | 7.50 | 65.60 | 7.50 |
| 21552154 | Huuskonen | 2011 | rs4537545 | Lean mass of legs | Caucasian | healthy males | C | T | 384 | 370 | 75 | 10.10 | 1.20 | 10.20 | 1.20 | 10.10 | 1.10 |
| 21552154 | Huuskonen | 2011 | rs4537545 | VO2max | Caucasian | healthy males | C | T | 384 | 370 | 75 | 41.80 | 7.80 | 41.80 | 8.30 | 40.10 | 8.60 |
| 21552154 | Huuskonen | 2011 | rs4537545 | Maximal force of leg extensors | Caucasian | healthy males | C | T | 384 | 370 | 75 | 2951 | 864.00 | 2944. | 872.00 | 2875 | 833.00 |
| 21552154 | Huuskonen | 2011 | rs4537545 | IL-6 level | Caucasian | healthy males | C | T | 384 | 370 | 75 | 1.10 | 1.20 | 1.20 | 1.30 | 1.30 | 1.10 |
| 21552154 | Huuskonen | 2011 | rs4537545 | IGF-1 level | Caucasian | healthy males | C | T | 384 | 370 | 75 | 30.90 | 7.70 | 31.00 | 7.40 | 31.90 | 7.60 |
| 20661738 | Rodriguez | 2011 | rs2228145 | sIL-6R level | Caucasian | rheumatoid arthritis patients | A | C | 14 | 15 | 10 | 35.27 | 3.50 | 45.50 | 4.58 | 52.55 | 3.18 |
| 20186139 | Jiang | 2010 | rs2228145 | BMI | Asian | healthy individuals | A | C | 859 | 894 | 238 | 23.90 | 3.10 | 23.60 | 2.90 | 23.60 | 3.10 |
| 20186139 | Jiang | 2010 | rs2228145 | CRP level | Asian | healthy individuals | A | C | 859 | 894 | 238 | 1.53 | 1.50 | 1.39 | 1.37 | 1.30 | 1.50 |
| 20186139 | Jiang | 2010 | rs2228145 | diastolic blood pressure | Asian | healthy individuals | A | C | 859 | 894 | 238 | 74.50 | 11.00 | 73.70 | 10.50 | 73.50 | 11.30 |
| 20186139 | Jiang | 2010 | rs2228145 | Glucose | Asian | healthy individuals | A | C | 859 | 894 | 238 | 5.67 | 1.59 | 5.51 | 1.27 | 5.56 | 1.44 |
| 20186139 | Jiang | 2010 | rs2228145 | HDL cholesterol level | Asian | healthy individuals | A | C | 859 | 894 | 238 | 1.58 | 0.41 | 1.58 | 0.38 | 1.63 | 0.42 |
| 20186139 | Jiang | 2010 | rs2228145 | IL-6 level | Asian | healthy individuals | A | C | 859 | 894 | 238 | 10.40 | 0.71 | 11.10 | 0.72 | 13.00 | 1.11 |
| 20186139 | Jiang | 2010 | rs2228145 | LDL cholesterol level | Asian | healthy individuals | A | C | 859 | 894 | 238 | 3.38 | 0.68 | 3.37 | 0.68 | 3.35 | 0.70 |
| 20186139 | Jiang | 2010 | rs2228145 | systolic blood pressure | Asian | healthy individuals | A | C | 859 | 894 | 238 | 128.00 | 21.00 | 127.00 | 20.00 | 127.00 | 21.00 |
| 20186139 | Jiang | 2010 | rs2228145 | Total cholesterol level | Asian | healthy individuals | A | C | 859 | 894 | 238 | 5.90 | 1.10 | 5.80 | 1.10 | 5.80 | 1.00 |
| 20186139 | Jiang | 2010 | rs2228145 | Triglycerides level | Asian | healthy individuals | A | C | 859 | 894 | 238 | 1.60 | 0.75 | 1.49 | 0.76 | 1.48 | 0.79 |
| 20186139 | Jiang | 2010 | rs2228145 | waist circumference | Asian | healthy individuals | A | C | 859 | 894 | 238 | 78.80 | 9.20 | 78.30 | 8.70 | 78.30 | 8.80 |
| 20186139 | Jiang | 2010 | rs2228145 | fibrinogen level | Asian | healthy individuals | A | C | 859 | 894 | 238 | 297.00 | 149.53 | 288.00 | 76.28 | 289.00 | 39.36 |
| 18852330 | Qi | 2009 | rs2228145 | CRP level | Caucasian | Healthy females | A | C | 234 | 342 | 101 | 0.32 | 0.02 | 0.26 | 0.02 | 0.24 | 0.03 |
| 18852330 | Qi | 2009 | rs2228145 | CRP level | Caucasian | diabetes patients | A | C | 227 | 308 | 88 | 0.63 | 0.03 | 0.48 | 0.03 | 0.43 | 0.05 |
| 18852330 | Qi | 2009 | rs2228145 | IL-6 level | Caucasian | Healthy females | A | C | 234 | 342 | 101 | 1.84 | 1.97 | 1.99 | 2.07 | 2.14 | 1.82 |
| 18852330 | Qi | 2009 | rs2228145 | IL-6 level | Caucasian | Healthy females | A | C | 234 | 342 | 101 | 26.20 | 6.60 | 26.30 | 5.60 | 27.60 | 6.60 |
| 18852330 | Qi | 2009 | rs2228145 | IL-6 level | Caucasian | Healthy females | A | C | 234 | 342 | 101 | 12.90 | 15.40 | 16.30 | 19.40 | 14.80 | 13.20 |
| 18852330 | Qi | 2009 | rs2228145 | IL-6 level | Caucasian | Healthy females | A | C | 234 | 342 | 101 | 6.34 | 11.00 | 5.54 | 9.62 | 5.31 | 7.87 |
| 18852330 | Qi | 2009 | rs6684439 | CRP level | Caucasian | Healthy females | C | T | 238 | 234 | 107 | 0.30 | 0.02 | 0.27 | 0.02 | 0.24 | 0.03 |
| 18852330 | Qi | 2009 | rs6684439 | CRP level | Caucasian | diabetes patients | C | T | 235 | 309 | 87 | 0.60 | 0.03 | 0.49 | 0.03 | 0.41 | 0.05 |
| 18852330 | Qi | 2009 | rs4845622 | CRP level | Caucasian | Healthy females | T | G | 225 | 355 | 105 | 0.31 | 0.02 | 0.27 | 0.02 | 0.24 | 0.03 |
| 18852330 | Qi | 2009 | rs4845622 | CRP level | Caucasian | diabetes patients | T | G | 225 | 312 | 96 | 0.61 | 0.03 | 0.49 | 0.03 | 0.41 | 0.05 |
| 18852330 | Qi | 2009 | rs4845618 | CRP level | Caucasian | Healthy females | A | C | 215 | 345 | 114 | 0.27 | 0.02 | 0.28 | 0.02 | 0.28 | 0.03 |
| 18852330 | Qi | 2009 | rs4845618 | CRP level | Caucasian | diabetes patients | A | C | 176 | 325 | 119 | 0.49 | 0.04 | 0.52 | 0.03 | 0.59 | 0.05 |
| 18852330 | Qi | 2009 | rs4845617 | CRP level | Caucasian | Healthy females | G | A | 246 | 317 | 114 | 0.27 | 0.02 | 0.26 | 0.02 | 0.30 | 0.03 |
| 18852330 | Qi | 2009 | rs4845617 | CRP level | Caucasian | diabetes patients | A | G | 223 | 299 | 99 | 0.52 | 0.03 | 0.51 | 0.03 | 0.57 | 0.05 |
| 18852330 | Qi | 2009 | rs4329505 | CRP level | Caucasian | Healthy females | T | C | 476 | 197 | 17 | 0.27 | 0.01 | 0.32 | 0.02 | 0.27 | 0.07 |
| 18852330 | Qi | 2009 | rs4329505 | CRP level | Caucasian | diabetes patients | T | C | 448 | 171 | 12 | 0.49 | 0.02 | 0.57 | 0.04 | 0.81 | 0.16 |
| 18852330 | Qi | 2009 | rs4240872 | CRP level | Caucasian | Healthy females | A | G | 405 | 253 | 27 | 0.27 | 0.01 | 0.29 | 0.02 | 0.27 | 0.06 |
| 18852330 | Qi | 2009 | rs4240872 | CRP level | Caucasian | diabetes patients | A | G | 362 | 237 | 32 | 0.51 | 0.03 | 0.53 | 0.03 | 0.61 | 0.09 |
| 18852330 | Qi | 2009 | rs4075015 | CRP level | Caucasian | Healthy females | A | T | 222 | 357 | 112 | 0.31 | 0.03 | 0.27 | 0.02 | 0.28 | 0.02 |
| 18852330 | Qi | 2009 | rs4075015 | CRP level | Caucasian | diabetes patients | A | T | 215 | 303 | 110 | 0.51 | 0.05 | 0.53 | 0.03 | 0.49 | 0.03 |
| 18852330 | Qi | 2009 | rs2229238 | CRP level | Caucasian | Healthy females | A | G | 462 | 218 | 17 | 0.27 | 0.01 | 0.30 | 0.02 | 0.32 | 0.08 |
| 18852330 | Qi | 2009 | rs2229238 | CRP level | Caucasian | diabetes patients | G | A | 412 | 198 | 19 | 0.50 | 0.02 | 0.56 | 0.04 | 0.54 | 0.11 |
| 18852330 | Qi | 2009 | rs12083537 | CRP level | Caucasian | Healthy females | T | C | 409 | 243 | 37 | 0.27 | 0.01 | 0.28 | 0.02 | 0.29 | 0.05 |
| 18852330 | Qi | 2009 | rs12083537 | CRP level | Caucasian | diabetes patients | C | T | 394 | 208 | 25 | 0.54 | 0.03 | 0.49 | 0.04 | 0.51 | 0.10 |
| 18464913 | Melzer | 2008 | rs4129267 | sIL-6R level | Caucasian | fasting individuals | C | T |  |  |  | 69.92 | 0.00 | 69.92 | 0.00 | 138.13 | 0.00 |
| 17984249 | Bustamante | 2007 | rs2228145 | BMI | Caucasian | postmenopausal women | A | C | 162 | 232 | 91 | 26.60 | 0.30 | 26.30 | 0.30 | 26.20 | 0.40 |
| 17984249 | Bustamante | 2007 | rs2228145 | BMD at FN | Caucasian | postmenopausal women | A | C | 162 | 232 | 91 | 0.66 | 0.01 | 0.68 | 0.01 | 0.69 | 0.02 |
| 17984249 | Bustamante | 2007 | rs2228145 | BMD at LS | Caucasian | postmenopausal women | A | C | 162 | 232 | 91 | 0.85 | 0.01 | 0.85 | 0.01 | 0.88 | 0.01 |
| 17984249 | Bustamante | 2007 | rs4845617 | BMD at LS | Caucasian | postmenopausal women |  | A | 179 | 214 | 71 | 0.85 | 0.01 | 0.85 | 0.01 | 0.85 | 0.02 |
| 17984249 | Bustamante | 2007 | rs4845617 | BMD at FN | Caucasian | postmenopausal women |  | A | 179 | 214 | 71 | 0.67 | 0.01 | 0.67 | 0.01 | 0.71 | 0.02 |
| 17898129 | Qi | 2007 | rs2228145 | IL-6 level | Caucasian | Healthy females | A | C | 239 | 347 | 105 | 0.51 | 0.04 | 0.69 | 0.04 | 0.76 | 0.07 |
| 17898129 | Qi | 2007 | rs2228145 | IL-6 level | Caucasian | diabetic women | A | C | 227 | 308 | 88 | 0.79 | 0.04 | 0.91 | 0.04 | 1.11 | 0.07 |
| 17898129 | Qi | 2007 | rs6684439 | IL-6 level | Caucasian | Healthy females | C | T | 238 | 234 | 107 | 0.76 | 0.07 | 0.67 | 0.04 | 0.87 | 0.06 |
| 17898129 | Qi | 2007 | rs6684439 | IL-6 level | Caucasian | diabetic women | C | T | 235 | 309 | 87 | 1.10 | 0.07 | 0.90 | 0.04 | 0.81 | 0.04 |
| 17898129 | Qi | 2007 | rs4845622 | IL-6 level | Caucasian | Healthy females | T | G | 225 | 355 | 105 | 0.51 | 0.05 | 0.68 | 0.04 | 0.75 | 0.07 |
| 17898129 | Qi | 2007 | rs4845622 | IL-6 level | Caucasian | diabetic women | T | G | 225 | 312 | 96 | 0.80 | 0.04 | 0.91 | 0.04 | 1.08 | 0.07 |
| 17898129 | Qi | 2007 | rs4845618 | IL-6 level | Caucasian | Healthy females | A | C | 215 | 345 | 114 | 0.50 | 0.06 | 0.63 | 0.04 | 0.72 | 0.05 |
| 17898129 | Qi | 2007 | rs4845618 | IL-6 level | Caucasian | diabetic women | A | C | 176 | 325 | 119 | 0.77 | 0.06 | 0.90 | 0.04 | 0.99 | 0.05 |
| 17898129 | Qi | 2007 | rs4845617 | IL-6 level | Caucasian | Healthy females | G | A | 246 | 317 | 114 | 0.66 | 0.04 | 0.61 | 0.04 | 0.68 | 0.06 |
| 17898129 | Qi | 2007 | rs4845617 | IL-6 level | Caucasian | diabetic women | G | A | 223 | 299 | 99 | 0.85 | 0.04 | 0.92 | 0.04 | 0.86 | 0.07 |
| 17898129 | Qi | 2007 | rs4329505 | IL-6 level | Caucasian | Healthy females | T | C | 476 | 197 | 17 | 0.65 | 0.03 | 0.64 | 0.05 | 0.45 | 0.17 |
| 17898129 | Qi | 2007 | rs4329505 | IL-6 level | Caucasian | diabetic women | T | C | 448 | 171 | 12 | 0.92 | 0.03 | 0.80 | 0.05 | 1.19 | 0.18 |
| 17898129 | Qi | 2007 | rs4240872 | IL-6 level | Caucasian | Healthy females | A | G | 405 | 253 | 27 | 0.69 | 0.03 | 0.57 | 0.04 | 0.53 | 0.13 |
| 17898129 | Qi | 2007 | rs4240872 | IL-6 level | Caucasian | diabetic women | A | G | 362 | 237 | 32 | 0.96 | 0.03 | 0.82 | 0.04 | 0.74 | 0.11 |
| 17898129 | Qi | 2007 | rs4075015 | IL-6 level | Caucasian | Healthy females | T | A | 222 | 357 | 112 | 0.61 | 0.05 | 0.66 | 0.04 | 0.68 | 0.07 |
| 17898129 | Qi | 2007 | rs4075015 | IL-6 level | Caucasian | diabetic women | T | A | 215 | 303 | 110 | 0.88 | 0.04 | 0.91 | 0.04 | 0.87 | 0.06 |
| 17898129 | Qi | 2007 | rs2229238 | IL-6 level | Caucasian | Healthy females | G | A | 462 | 218 | 17 | 0.64 | 0.17 | 0.55 | 0.05 | 0.68 | 0.03 |
| 17898129 | Qi | 2007 | rs2229238 | IL-6 level | Caucasian | diabetic women | G | A | 412 | 198 | 19 | 0.65 | 0.15 | 0.86 | 0.05 | 0.92 | 0.03 |
| 17898129 | Qi | 2007 | rs12083537 | IL-6 level | Caucasian | Healthy females | T | C | 409 | 243 | 37 | 0.64 | 0.03 | 0.62 | 0.04 | 0.76 | 0.11 |
| 17898129 | Qi | 2007 | rs12083537 | IL-6 level | Caucasian | diabetic women | T | C | 394 | 208 | 25 | 0.92 | 0.03 | 0.85 | 0.05 | 0.99 | 0.14 |
| 17671508 | Rafiq | 2007 | rs4537545 | Gp-130 (ng/ml) | Caucasian | healthy individuals | C | T | 454 | 617 | 202 | 307.24 | 59.25 | 308.52 | 65.52 | 301.03 | 81.80 |
| 17671508 | Rafiq | 2007 | rs4537545 | WBC count (n k/ml) | Caucasian | healthy individuals | C | T | 454 | 617 | 202 | 6.10 | 1.41 | 5.95 | 1.52 | 6.14 | 1.52 |
| 17671508 | Rafiq | 2007 | rs4537545 | Neutrophils count (n k/ml) | Caucasian | healthy individuals | C | T | 454 | 617 | 202 | 3.63 | 1.20 | 3.55 | 1.14 | 3.70 | 1.60 |
| 17671508 | Rafiq | 2007 | rs4537545 | fibrinogen level | Caucasian | healthy individuals | C | T | 454 | 617 | 202 | 344.96 | 121.65 | 350.92 | 201.00 | 330.97 | 82.74 |
| 17671508 | Rafiq | 2007 | rs4537545 | Erythrocyte | Caucasian | healthy individuals | C | T | 454 | 617 | 202 | 14.89 | 12.94 | 13.91 | 11.28 | 15.40 | 11.75 |
| 17671508 | Rafiq | 2007 | rs4537545 | sedimentation rate CRP | Caucasian | healthy individuals | C | T | 454 | 617 | 202 | 2.61 | 2.83 | 2.44 | 2.66 | 2.40 | 2.61 |
| 17671508 | Rafiq | 2007 | rs4537545 | IL-1RA level | Caucasian | healthy individuals | C | T | 454 | 617 | 202 | 127.92 | 71.42 | 132.45 | 78.83 | 132.06 | 69.83 |
| 17671508 | Rafiq | 2007 | rs4537545 | a-2 macroglobulin (mg/dl) | Caucasian | healthy individuals | C | T | 454 | 617 | 202 | 200.76 | 59.90 | 197.98 | 58.04 | 199.43 | 53.15 |
| 17671508 | Rafiq | 2007 | rs4537545 | Fasting insulina level | Caucasian | healthy individuals | C | T | 454 | 617 | 202 | 68.33 | 34.03 | 69.03 | 35.87 | 61.05 | 32.99 |
| 17671508 | Rafiq | 2007 | rs4537545 | Fasting glucose level | Caucasian | healthy individuals | C | T | 454 | 617 | 202 | 5.19 | 0.98 | 5.07 | 0.89 | 4.98 | 0.58 |
| 17671508 | Rafiq | 2007 | rs4537545 | HDL cholesterol level | Caucasian | healthy individuals | C | T | 454 | 617 | 202 | 54.24 | 14.57 | 53.79 | 13.81 | 52.90 | 13.78 |
| 17671508 | Rafiq | 2007 | rs4537545 | LDL cholesterol level | Caucasian | healthy individuals | C | T | 454 | 617 | 202 | 134.57 | 35.55 | 132.88 | 35.23 | 135.38 | 35.39 |
| 17671508 | Rafiq | 2007 | rs4537545 | Total cholesterol level | Caucasian | healthy individuals | C | T | 454 | 617 | 202 | 216.84 | 40.66 | 212.98 | 41.57 | 214.62 | 41.48 |
| 17671508 | Rafiq | 2007 | rs4537545 | Triglycerides level | Caucasian | healthy individuals | C | T | 454 | 617 | 202 | 113.93 | 54.68 | 108.88 | 50.44 | 108.76 | 51.92 |
| 17671508 | Rafiq | 2007 | rs4537545 | Adiponectin level | Caucasian | healthy individuals | C | T | 454 | 617 | 202 | 11.05 | 8.15 | 10.95 | 8.24 | 9.29 | 7.18 |
| 17671508 | Rafiq | 2007 | rs4537545 | BMI | Caucasian | healthy individuals | C | T | 454 | 617 | 202 | 27.17 | 4.02 | 27.14 | 4.31 | 27.26 | 4.06 |
| 17671508 | Rafiq | 2007 | rs4537545 | Waist/hip ratio | Caucasian | healthy individuals | C | T | 454 | 617 | 202 | 0.90 | 0.08 | 0.91 | 0.08 | 0.92 | 0.08 |
| 17671508 | Rafiq | 2007 | rs4537545 | IL6R level | Caucasian | healthy individuals | C | T | 454 | 617 | 202 | 68.31 | 32.72 | 98.58 | 46.64 | 132.48 | 53.52 |
| 17671508 | Rafiq | 2007 | rs4537545 | IL-6 level | Caucasian | healthy individuals | C | T | 454 | 617 | 202 | 1.22 | 0.98 | 1.32 | 1.01 | 1.57 | 1.38 |
| 17357077 | Reich | 2007 | rs2228145 | IL-6 level | African American | healthy males | A | C | 419 | 157 | 16 | 2.55 | 2.14 | 2.71 | 1.84 | 3.39 | 2.29 |
| 17357077 | Reich | 2007 | rs2228145 | IL-6 level | Caucasian | healthy males | A | C | 278 | 400 | 127 | 2.16 | 1.87 | 2.25 | 1.74 | 2.53 | 1.83 |
| 17357077 | Reich | 2007 | rs2228145 | sIL-6R level | African American | healthy males | A | C | 419 | 157 | 16 | 29665 | 5245.0 | 38894 | 8735.0 | 46543 | 7286.00 |
| 17357077 | Reich | 2007 | rs2228145 | sIL-6R level | Caucasian | healthy males | A | C | 278 | 400 | 127 | 31476. | 5188 | 40790 | 6323 | 49371 | 8129.00 |
| 15561970 | Hamid | 2004 | rs2228145 | BMI | Caucasian | glucose tolerant individuals | A | C | 1495 | 2013 | 743 | 25.60 | 4.10 | 25.50 | 4.00 | 25.40 | 4.00 |
| 15561970 | Hamid | 2004 | rs2228145 | HDL cholesterol level | Caucasian | glucose tolerant individuals | A | C | 1495 | 2013 | 743 | 1.45 | 0.40 | 1.47 | 0.40 | 1.47 | 0.40 |
| 15561970 | Hamid | 2004 | rs2228145 | Total cholesterol level | Caucasian | glucose tolerant individuals | A | C | 1495 | 2013 | 743 | 5.40 | 1.00 | 5.40 | 1.00 | 5.40 | 1.00 |
| 15561970 | Hamid | 2004 | rs2228145 | Triglycerides level | Caucasian | glucose tolerant individuals | A | C | 1495 | 2013 | 743 | 1.16 | 0.70 | 1.18 | 0.80 | 1.21 | 1.53 |
| 15561970 | Hamid | 2004 | rs2228145 | glucose at 0 min (mmol/l) | Caucasian | glucose tolerant individuals | A | C | 1495 | 2013 | 743 | 5.30 | 0.40 | 5.30 | 0.40 | 5.30 | 0.40 |
| 15561970 | Hamid | 2004 | rs2228145 | glucose at 120 min (mmol/l) | Caucasian | glucose tolerant individuals | A | C | 1495 | 2013 | 743 | 5.50 | 1.10 | 5.50 | 1.10 | 5.40 | 1.10 |
| 15561970 | Hamid | 2004 | rs2228145 | HOMA-IR | Caucasian | glucose tolerant individuals | A | C | 1495 | 2013 | 743 | 9.10 | 5.70 | 8.80 | 5.60 | 8.80 | 5.70 |
| 15561970 | Hamid | 2004 | rs2228145 | Incremental AUC glucose | Caucasian | glucose tolerant individuals | A | C | 1495 | 2013 | 743 | 181.00 | 104.00 | 184.00 | 99.00 | 179.00 | 98.00 |
| 15561970 | Hamid | 2004 | rs2228145 | Incremental AUC insulin | Caucasian | glucose tolerant individuals | A | C | 1495 | 2013 | 743 | 21.15 | 13.36 | 20.88 | 13.29 | 20.78 | 12.81 |
| 15561970 | Hamid | 2004 | rs2228145 | insulin at 0 min (pmol/l) | Caucasian | glucose tolerant individuals | A | C | 1495 | 2013 | 743 | 38.00 | 24.00 | 37.00 | 23.00 | 37.00 | 23.00 |
| 15561970 | Hamid | 2004 | rs2228145 | insulin at 120 min (pmol/l) | Caucasian | glucose tolerant individuals | A | C | 1495 | 2013 | 743 | 167.00 | 121.00 | 167.00 | 133.00 | 169.00 | 139.00 |
| 15561970 | Hamid | 2004 | rs2228145 | Insulinogenic index insulin | Caucasian | glucose tolerant individuals | A | C | 1495 | 2013 | 743 | 31.00 | 21.00 | 31.00 | 19.00 | 31.00 | 18.00 |
| 15561970 | Hamid | 2004 | rs2228145 | Waist/hip ratio | Caucasian | glucose tolerant individuals | A | C | 1495 | 2013 | 743 | 0.84 | 0.09 | 0.84 | 0.08 | 0.84 | 0.08 |
| 15306846 | Galicia | 2004 | rs2228145 | sIL-6R level | Asian | healthy individuals | A | C | 44 | 47 | 27 | 23.80 | 71.00 | 29.70 | 71.40 | 39.70 | 72.80 |
| 14680981 | Wolford | 2003 | rs2228145 | BMI | Other | T2DM patients | A | C | 375 | 338 | 73 | 35.80 | 7.60 | 36.80 | 7.50 | 38.10 | 7.10 |
| 14680981 | Wolford | 2003 | rs4845623 | BMI | Other | T2DM patients | T | C | 67 | 320 | 322 | 38.20 | 7.00 | 36.70 | 7.50 | 35.80 | 7.60 |
| 14680981 | Wolford | 2003 | rs2229238 | BMI | Other | T2DM patients | C | T | 473 | 277 | 34 | 36.00 | 7.60 | 36.90 | 7.50 | 37.80 | 6.70 |

**Supplementary Table 4**. **Polymorphism rs2228145 associated with continuous quantitative phenotypes in meta-analysis**

| **Phenotypes** | **Datasets** | **Genotype** | | **Mean difference of**  **quantitative phenotypes** | | **Heterogeneity** | |
| --- | --- | --- | --- | --- | --- | --- | --- |
|  |  | **AA** | **CC** | **SMD (95%CI)** | ***P*** | ***І*^2^** | ***P*** |
| CRP level | 4 | 2,496 | 1,028 | -2.250 (-3.740, -0.759) | 0.003 | 99.6% | <0.001 |
| IL-6 level | 10 | 3,432 | 1,276 | 1.551 (0.610, 2.491) | 0.001 | 99.4% | <0.001 |
| sIL-6R level | 6 | 5,009 | 5,918 | 1.856 (-0.228, 3.940) | 0.081 | 99.5% | <0.001 |
| Glucose | 4 | 1,011 | 306 | -0.097 (-0.223, 0.029) | 0.133 | 0.0% | 0.829 |
| HDL cholesterol level | 7 | 2,815 | 1,181 | 0.058 (-0.044, 0.161) | 0.263 | 28.0% | 0.215 |
| LDL cholesterol level | 4 | 1,292 | 425 | 0.118 (-0.052, 0.287) | 0.173 | 46.0% | 0.135 |
| Total cholesterol level | 5 | 2,787 | 1,168 | -0.003 (-0.072, 0.066) | 0.93 | 0.0% | 0.478 |
| Triglycerides level | 7 | 2,815 | 1,181 | -0.003 (-0.173, 0.166) | 0.97 | 69.7% | 0.003 |
| systolic blood pressure | 3 | 887 | 251 | 0.446 (-0.240, 1.132) | 0.202 | 82.0% | 0.004 |
| diastolic blood pressure | 3 | 887 | 251 | 0.144 (-0.283, 0.572) | 0.508 | 58.0% | 0.092 |
| waist circumference | 3 | 887 | 251 | 0.402 (-0.233, 1.038) | 0.215 | 79.3% | 0.008 |
| BMI | 6 | 3,037 | 1,227 | -0.227 (-0.539, 0.085) | 0.153 | 92.9% | <0.001 |
|  |  | **AA** | **AC** |  |  |  |  |
| CRP level | 4 | 2,496 | 3,165 | -2.010 (-3.360, -0.660) | 0.004 | 99.8% | <0.001 |
| IL-6 level | 10 | 3,432 | 4,069 | 0.883 (0.310, 1.456) | 0.003 | 99.4% | <0.001 |
| sIL-6R level | 6 | 5,009 | 3,790 | 0.842 (-0.400, 2.084) | 0.184 | 99.5% | <0.001 |
| Glucose | 4 | 1,011 | 1,035 | -0.010 (-0.176, 0.155) | 0.905 | 35.2% | 0.201 |
| HDL cholesterol level | 7 | 2,815 | 3,466 | 0.025 (-0.024, 0.075) | 0.32 | 0.0% | 0.692 |
| LDL cholesterol level | 4 | 1,292 | 1,417 | 0.015 (-0.061, 0.090) | 0.706 | 0.0% | 0.716 |
| Total cholesterol level | 5 | 2,787 | 3,430 | -0.021 (-0.071, 0.030) | 0.423 | 0.0% | 0.488 |
| Triglycerides level | 7 | 2,815 | 3,466 | 0.048 (-0.098, 0.195) | 0.518 | 79.3% | <0.001 |
| systolic blood pressure | 3 | 887 | 930 | 0.220 (-0.183, 0.623) | 0.284 | 72.5% | 0.026 |
| diastolic blood pressure | 3 | 887 | 930 | 0.155 (-0.203, 0.513) | 0.396 | 66.0% | 0.053 |
| waist circumference | 3 | 887 | 930 | 0.424 (-0.201, 1.050) | 0.184 | 88.1% | <0.001 |
| BMI | 6 | 3,037 | 3,678 | -0.196 (-0.433, 0.041) | 0.105 | 94.0% | <0.001 |

CRP: C reactive protein, IL-6: interleukin 6, IL-6R: interleukin 6 receptor, sIL-6R: soluble IL-6R, BMI: body mass index, SMD: standardized mean difference

**Supplementary Table 5. Details of protection from bias for genetic variants significantly associated with risk of diseases and phenotypes.**

| **Variant** | **Diseases** | **Protection from bias** | **Reason for bias exemption** | **Reason for bias** | **Initial study influence** | | **Deviati-on from HWE** | **OR<1.15 or OR>0.87** | ***P* value for small study bias** | ***P* value for public-ation bias** |
| --- | --- | --- | --- | --- | --- | --- | --- | --- | --- | --- |
|  |  |  |  |  | **OR (95%CI)** | ***P*** |  |  |  |  |
| **rs2228145** | aneurysms | A | Replicated across studies from large collaborative consortia | Low OR | 0.892 (0.862, 0.922) | 1.20×10^-11^ | No | Yes | 0.126 | 0.221 |
|  | atherosclerosis | A | Replicated across studies from large collaborative consortia | Low OR, Small study | 0.930 (0.904, 0.957) | 7.05×10^-07^ | No | Yes | 0.033 | 0.089 |
|  | coronary heart disease | A | Identified by GWAS | Low OR | 0.955 (0.943, 0.966) | 4.70×10^-14^ | No | Yes | 0.644 | 0.452 |
|  | rheumatoid arthritis | A | Identified by GWAS | NA | 0.568 (0.379, 0.852) | 0.006 | No | No | 0.100 | 0.133 |
|  | type 1 diabetes | A | Replicated across studies from large collaborative consortia | Low OR, Small study | 0.950 (0.921, 0.980) | 0.001 | No | Yes | 0.003 | 0.089 |
|  | Crohn’s disease | A | Identified by GWAS | Low OR | 0.946 (0.927, 0.964) | 2.27×10^-08^ | No | Yes | 0.905 | 1.000 |
|  | ulcerative colitis | A | Identified by GWAS | Low OR | 0.976 (0.957, 0.996) | 0.018 | No | Yes | 0.190 | 0.296 |
|  | dermatitis | A | Replicated across studies from large collaborative consortia | Low OR, Small study | 1.035 (1.025, 1.045) | 2.95×10^-12^ | No | Yes | 0.018 | 0.043 |
|  | asthma | A | Replicated across studies from large collaborative consortia | Low OR | 1.056 (1.019, 1.095) | 0.003 | No | Yes | 0.611 | 1.000 |
|  | type 2 diabetes | A | Replicated across studies from large collaborative consortia | Low OR | 0.980 (0.971, 0.990) | 6.07×10^-05^ | No | Yes | 0.492 | 0.806 |
|  | cardiovascular disease | C | NA | Initial study, Small study | 0.806 (0.616, 1.054) | 0.115 | No | No | 0.076 | 0.308 |
|  | CRP level | A | Replicated across studies from large collaborative consortia | Low OR, Small study | 0.910 (0.893, 0.927) | 3.67×10^-24^ | No | Yes | 0.023 | 0.547 |
|  | fibrinogen level | A | Identified by GWAS | Small study | 0.964 (0.932, 0.997) | 0.032 | No | Yes | 0.055 | 1.000 |
|  | sIL-6R level | A | NA | NA | 1.338 (1.315, 1.360) | 2.65×10^-255^ | No | No | 0.395 | 0.707 |
|  | IL-6 level | A | Replicated across studies from large collaborative consortia | Low OR, Small study | 1.124 (1.077, 1.173) | 7.87×10^-08^ | No | Yes | 0.018 | 0.130 |
| **rs28638007** | atrial fibrillation | C | NA | Low OR | NA | NA | NA | Yes | NA | NA |
| **rs4129267** | abdominal aortic aneurysms | A | Identified by GWAS | Small study | 1.171 (1.116, 1.230) | 1.77×10^-10^ | No | No | 0.024 | 0.368 |
|  | asthma | A | Identified by GWAS | Low OR | 1.062 (1.025, 1.102) | 0.001 | No | Yes | 0.502 | 0.452 |
|  | CRP level | A | Replicated across studies from large collaborative consortia | Low OR | 0.915 (0.903, 0.927) | 2.06×10^-38^ | No | Yes | 0.386 | 1.000 |
|  | fibrinogen level | A | Identified by GWAS | Low OR | 0.955 (0.938, 0.973) | 8.22×10^-07^ | No | Yes | NA | 1.000 |
|  | IL-6 level | A | Replicated across studies from large collaborative consortia | Low OR | 1.083 (1.062, 1.105) | 1.24×10^-15^ | No | Yes | 0.511 | 0.540 |
|  | IL6R level | C | NA | Initial study | 1.314 (0.956, 1.806) | 0.092 | No | No | 0.360 | 0.296 |
| **rs4537545** | CRP level | A | Identified by GWAS | Low OR | 0.920 (0.880, 0.962) | 2.33×10^-04^ | No | Yes | 0.626 | 0.805 |
|  | fibrinogen level | A | Identified by GWAS | Low OR | 0.952 (0.935, 0.970) | 1.99×10^-07^ | No | Yes | NA | 1.000 |
|  | IL-6 level | A | Replicated across studies from large collaborative consortia | Low OR | 1.098 (1.071, 1.126) | 3.10×10^-13^ | No | Yes | 0.525 | 0.540 |
|  | coronary heart disease | C | NA | Low OR | 0.944 (0.903, 0.987) | 0.012 | No | Yes | 0.239 | 0.386 |
|  | atrial fibrillation | C | NA | Low OR | NA | NA | NA | Yes | NA | NA |
| **rs4553185** | IL-6 level | A | Replicated across studies from large collaborative consortia | Low OR | 0.942 (0.923, 0.960) | 1.97×10^-09^ | No | Yes | 0.484 | 0.540 |
| **rs4845371** | CRP level | A | Replicated across studies from large collaborative consortia | Low OR | 0.923 (0.898, 0.948) | 5.54×10^-09^ | No | Yes | 0.140 | 0.497 |
| **rs4845618** | IL-6 level | A | Replicated across studies from large collaborative consortia | Low OR | 0.946 (0.933, 0.960) | 7.25×10^-15^ | No | Yes | 0.272 | 0.540 |
| **rs4845625** | coronary artery disease | A | Identified by GWAS | Low OR | 1.061 (1.042, 1.080) | 1.20×10^-10^ | No | Yes | 0.279 | 0.223 |
|  | atrial fibrillation | A | Identified by GWAS | NA | 1.265 (1.050, 1.524) | 0.014 | No | No | 0.340 | 0.296 |
|  | CRP level | C | NA | Low OR | 1.114 (1.019, 1.219) | 0.018 | No | Yes | NA | 0.602 |
| **rs6667434** | CRP level | A | Replicated across studies from large collaborative consortia | Low OR | 0.923 (0.898, 0.948) | 5.54×10^-09^ | No | Yes | 0.140 | 0.497 |
| **rs7518199** | fibrinogen level | A | Identified by GWAS | Low OR | NA | NA | No | Yes | NA | NA |
|  | IL-6 level | A | Replicated across studies from large collaborative consortia | Low OR | 1.083 (1.040, 1.127) | 1.00×10^-04^ | No | Yes | 0.704 | 1.000 |
| **rs7529229** | coronary heart disease | A | Replicated across studies from large collaborative consortia | Low OR | 0.954 (0.930, 0.977) | 1.51×10^-04^ | No | Yes | 0.746 | 0.693 |
|  | atrial fibrillation | C | NA | Low OR | NA | NA | No | Yes | NA | NA |
|  | abdominal aortic aneurysms | A | Replicated across studies from large collaborative consortia | Small study | 0.851 (0.806, 0.899) | 7.47×10^-09^ | No | No | 0.077 | 0.221 |
|  | CRP level | A | Replicated across studies from large collaborative consortia | Low OR | 0.913 (0.905, 0.922) | 2.50×10^-80^ | No | Yes | 0.625 | 1.000 |
|  | IL-6 level | A | Replicated across studies from large collaborative consortia | Low OR | 1.083 (1.062, 1.105) | 1.24×10^-15^ | No | Yes | 0.566 | 0.540 |

Note: HWE: Hardy-Weinberg equilibrium, IL-6: interleukin 6, IL-6R: interleukin 6 receptor, sIL-6R: soluble IL-6R, CRP: C reactive protein. NA: Not available.

**Supplementary Table 6. Correlations (r2) among the ten variants showing strong evidence using data from the 1000 Genomes Project.**

| **European** |  |  |  |  |  |  |  |  |  |  |
| --- | --- | --- | --- | --- | --- | --- | --- | --- | --- | --- |
| Variants | rs4845618 | rs7518199 | rs4845371 | rs6667434 | rs4553185 | rs4537545 | rs7529229 | rs4845625 | rs4129267 | rs2228145 |
| rs4845618 | 1 |  |  |  |  |  |  |  |  |  |
| rs7518199 | 0.465 | 1 |  |  |  |  |  |  |  |  |
| rs4845371 | 0.93 | 0.458 | 1 |  |  |  |  |  |  |  |
| rs6667434 | 0.93 | 0.458 | 1 | 1 |  |  |  |  |  |  |
| rs4553185 | 0.98 | 0.482 | 0.949 | 0.949 | 1 |  |  |  |  |  |
| rs4537545 | 0.391 | 0.876 | 0.453 | 0.453 | 0.406 | 1 |  |  |  |  |
| rs7529229 | 0.396 | 0.884 | 0.453 | 0.453 | 0.412 | 0.992 | 1 |  |  |  |
| rs4845625 | 0.865 | 0.423 | 0.925 | 0.925 | 0.883 | 0.485 | 0.491 | 1 |  |  |
| rs4129267 | 0.433 | 0.933 | 0.426 | 0.426 | 0.449 | 0.934 | 0.942 | 0.462 | 1 |  |
| rs2228145 | 0.433 | 0.933 | 0.426 | 0.426 | 0.449 | 0.934 | 0.942 | 0.462 | 1 | 1 |
| **Asian** |  |  |  |  |  |  |  |  |  |  |
| Variants | rs4845618 | rs7518199 | rs4845371 | rs6667434 | rs4553185 | rs4537545 | rs7529229 | rs4845625 | rs4129267 | rs2228145 |
| rs4845618 | 1 |  |  |  |  |  |  |  |  |  |
| rs7518199 | 0.643 | 1 |  |  |  |  |  |  |  |  |
| rs4845371 | 0.964 | 0.647 | 1 |  |  |  |  |  |  |  |
| rs6667434 | 0.964 | 0.647 | 1 | 1 |  |  |  |  |  |  |
| rs4553185 | 0.964 | 0.647 | 1 | 1 | 1 |  |  |  |  |  |
| rs4537545 | 0.594 | 0.926 | 0.611 | 0.611 | 0.611 | 1 |  |  |  |  |
| rs7529229 | 0.582 | 0.912 | 0.599 | 0.599 | 0.599 | 0.959 | 1 |  |  |  |
| rs4845625 | 0.89 | 0.575 | 0.917 | 0.917 | 0.917 | 0.605 | 0.634 | 1 |  |  |
| rs4129267 | 0.588 | 0.904 | 0.605 | 0.605 | 0.605 | 0.951 | 0.991 | 0.64 | 1 |  |
| rs2228145 | 0.584 | 0.9 | 0.601 | 0.601 | 0.601 | 0.946 | 0.986 | 0.636 | 0.995 | 1 |
| **African** |  |  |  |  |  |  |  |  |  |  |
| Variants | rs4845618 | rs7518199 | rs4845371 | rs6667434 | rs4553185 | rs4537545 | rs7529229 | rs4845625 | rs4129267 | rs2228145 |
| rs4845618 | 1 |  |  |  |  |  |  |  |  |  |
| rs7518199 | 0.264 | 1 |  |  |  |  |  |  |  |  |
| rs4845371 | 0.419 | 0.121 | 1 |  |  |  |  |  |  |  |
| rs6667434 | 0.419 | 0.121 | 1 | 1 |  |  |  |  |  |  |
| rs4553185 | 0.429 | 0.003 | 0.381 | 0.381 | 1 |  |  |  |  |  |
| rs4537545 | 0.2 | 0.112 | 0.625 | 0.625 | 0.172 | 1 |  |  |  |  |
| rs7529229 | 0.205 | 0.11 | 0.638 | 0.638 | 0.181 | 0.977 | 1 |  |  |  |
| rs4845625 | 0.336 | 0.095 | 0.814 | 0.814 | 0.311 | 0.788 | 0.808 | 1 |  |  |
| rs4129267 | 0.093 | 0.344 | 0.042 | 0.042 | 0.118 | 0.052 | 0.051 | 0.041 | 1 |  |
| rs2228145 | 0.098 | 0.361 | 0.041 | 0.041 | 0.113 | 0.361 | 0.049 | 0.04 | 0.963 | 1 |

**Supplementary Table 7.**  Analyses of expression quantitative trait locus (eQTL) in the two independent loci.

|  | **GTEx** | | | | **Blood eQTL browser** | | |
| --- | --- | --- | --- | --- | --- | --- | --- |
| **Variants** | **Gene Symbol** | **Tissue** | **Effect size** | **P-Value** | **Gene Symbol** | **Tissue** | **P-Value** |
| rs2228145 | IL6R | Artery | -0.19 | 1.30×10^-16^ | IL6R | Whole_Blood | 3.155×10^-27^ |
|  | IL6R | Colon | -0.31 | 1.00×10^-12^ | UBE2Q1 | Whole_Blood | 9.746×10^-08^ |
|  | IL6R | Whole Blood | -0.087 | 5.80×10^-09^ |  |  |  |
|  | IL6R | Esophagus | -0.17 | 1.50×10^-05^ |  |  |  |
|  | SHE | Testis | -0.19 | 1.00×10^-06^ |  |  |  |
|  | TDRD10 | Visceral Adipose | 0.17 | 1.40×10^-09^ |  |  |  |
|  | TDRD10 | Esophagus | 0.15 | 1.00×10^-07^ |  |  |  |
|  | TDRD10 | Thyroid | 0.14 | 7.20×10^-06^ |  |  |  |
|  | TDRD10 | Lung | 0.13 | 1.60×10^-04^ |  |  |  |
| rs4845625 | IL6R | Testis | -0.45 | 4.30×10^-20^ | IL6R | Whole_Blood | 3.216×10^-22^ |
|  | IL6R | Whole Blood | -0.11 | 9.40×10^-15^ | UBAP2L | Whole_Blood | 0.002 |
|  | IL6R | Colon | -0.29 | 3.30×10^-12^ | AQP10 | Whole_Blood | 6.72×10^-05^ |
|  | IL6R | Artery | -0.15 | 9.10×10^-11^ |  |  |  |
|  | IL6R | Heart | -0.21 | 1.70×10^-05^ |  |  |  |
|  | IL6R | Small Intestine | -0.25 | 2.10×10^-05^ |  |  |  |
|  | PSMD8P1 | Whole Blood | -0.16 | 1.30×10^-04^ |  |  |  |
|  | TDRD10 | Lung | 0.25 | 3.30×10^-15^ |  |  |  |
|  | TDRD10 | Thyroid | 0.18 | 9.50×10^-11^ |  |  |  |
|  | TDRD10 | Esophagus | 0.17 | 2.10×10^-09^ |  |  |  |
|  | TDRD10 | Testis | -0.15 | 7.80×10^-09^ |  |  |  |
|  | TDRD10 | Visceral Adipose | 0.15 | 1.40×10^-07^ |  |  |  |
|  | TDRD10 | Adrenal Gland | 0.21 | 6.90×10^-06^ |  |  |  |
|  | TDRD10 | Pituitary | 0.23 | 8.10×10^-06^ |  |  |  |
|  | TDRD10 | Skin | 0.11 | 5.20×10^-05^ |  |  |  |
|  | TDRD10 | Breast | 0.11 | 7.60×10^-05^ |  |  |  |
|  | TDRD10 | Artery | 0.097 | 1.10×10^-04^ |  |  |  |
